# 2025 update to European Stroke Organisation (ESO) guideline on blood pressure management in acute ischaemic stroke and intracerebral haemorrhage

**DOI:** 10.1093/esj/aakag004

**Published:** 2026-05-07

**Authors:** Else Charlotte Sandset, Lina Palaiodimou, Silje Holt Jahr, Leonard Ho, Urs Fischer, Aristeidis H Katsanos, Kailash Krishnan, Benjamin Maïer, Eva A Mistry, Simona Sacco, Silvia Schönenberger, Thorsten Steiner, Georgios Tsivgoulis

**Affiliations:** Department of Neurology, Institute of Clinical Medicine, University of Oslo, Oslo, Norway; Second Department of Neurology, School of Medicine, National and Kapodistrian University of Athens, “Attikon” University Hospital, Athens, Greece; Department of Neurology, Akershus University Hospital, Lørenskog, Norway; Institute of Clinical Medicine, Faculty of Medicine, University of Oslo, Oslo, Norway; European Stroke Organisation (ESO), Basel, Switzerland; Stroke/Neurology, University Hospital Bern, Bern, Switzerland; Deparment of Medicine (Neurology), McMaster University & Population Health Research Institute, Hamilton, Canada; Stroke, Department of Acute Medicine, Queen’s Medical Centre, Nottingham University Hospitals NHS Trust, Nottingham, United Kingdom; Neurology Department, Hôpital Paris Saint-Joseph, Université Paris Cité, Paris, France; Interventional Neuroradiology Department, Hôpital Fondation A. de Rothschild, Paris, France; Department of Neurology and Rehabilitation Medicine, University of Cincinnati, Cincinnati, United States; Department of Biotechnological and Applied Clinical Sciences, University of L'Aquila, L'Aquila, Italy; Neurology and Stroke Unit, ASL 1 Avezzano-Sulmona-L’Aquila, L’Aquila, Italy; Department of Neurology, Heidelberg University Hospital, Heidelberg, Germany; Department of Neurology, Heidelberg University Hospital, Heidelberg, Germany; Neurology / Neurointensive Care, Department of Neurology, Varisano Klinikum Frankfurt, Frankfurt, Germany; Second Department of Neurology, School of Medicine, National and Kapodistrian University of Athens, “Attikon” University Hospital, Athens, Greece

**Keywords:** blood pressure, guideline, stroke, systematic review

## Abstract

Optimal blood pressure (BP) management in acute ischaemic stroke (AIS) and acute ICH remains uncertain. In light of new data published since the previous ESO guidelines, this update provides revised, evidence-based recommendations across 8 key clinical questions to support BP management in acute stroke. The guidelines were developed using the ESO standard operating procedure and Grading of Recommendations, Assessment, Development and Evaluation (GRADE) methodology, including literature searches, systematic reviews and meta-analyses of relevant RCTs, assessment of evidence quality and formulation of specific recommendations. We advise against routine pre-hospital BP lowering in suspected stroke (moderate-certainty evidence). In AIS patients undergoing reperfusion therapy, we recommend maintaining BP < 185/110 mmHg before the bolus of intravenous thrombolysis and < 180/105 mmHg during and for 24 h after intravenous thrombolysis (low-certainty evidence) and/or mechanical thrombectomy (moderate-certainty evidence). We recommend against intensively lowering systolic BP < 140 mmHg in the first 24 h after successful mechanical thrombectomy (high-certainty evidence). Routine use of vasopressors to raise BP in AIS patients with neurological deterioration who are not treated with acute reperfusion therapies is discouraged (low-certainty evidence). In acute ICH, the net clinical benefit of intensive BP lowering remains uncertain; however, expert consensus supports early systolic BP reduction to < 140 mmHg in patients with small-to-moderate haematomas to limit haematoma expansion. Overall, the updated recommendations reaffirm the core principles of current clinical practice while providing more nuanced guidance for specific scenarios. However, the quality of evidence remains moderate to very low, limited by a lack of high-quality RCTs, methodological issues, inconsistent results and study heterogeneity. Consequently, most recommendations are weak and supported by expert consensus. These guidelines provide specific recommendations on BP thresholds and management strategies tailored to distinct acute stroke subgroups. They also highlight the ongoing uncertainty and emphasise the need for future RCTs to define optimal BP targets, timing, treatment strategies and ideal antihypertensive agents across different clinical contexts.

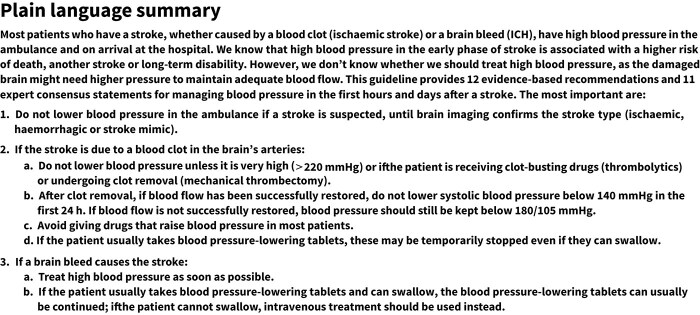

## Graphical Abstract

**Graphical Abstract:**
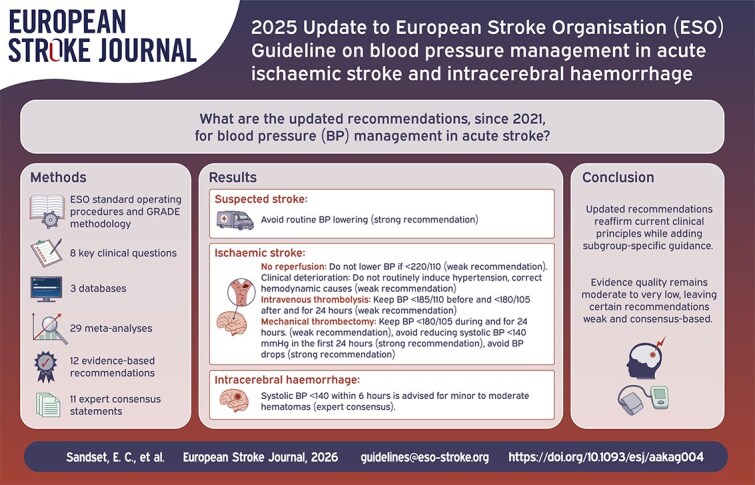

## Table of Contents

Abstract 1

Plain language summary 2

Graphical Abstract 2

Introduction 3

Methods 3

 Composition and approval of the Module Working Group 3

 Development and approval of clinical questions 5

Results 6

 PICO 1

  Analysis of current evidence 6

  Additional information 8

 PICO 2

  Analysis of current evidence 10

  Additional information 12

 PICO 3

  Analysis of current evidence 15

  Additional information 17

 PICO 4

  Analysis of current evidence 19

  Additional information 21

 PICO 5

  Analysis of current evidence 25

  Additional information 25

 PICO 6

  Analysis of current evidence 28

  Additional information 29

 PICO 7

   of current evidence 31

  Additional information 40

 PICO 8

  Analysis of current evidence 43

  Additional information 43

Discussion 44

References 49

## Introduction

Elevated blood pressure (BP) levels (systolic BP [SBP ≥ 140 mmHg and/or diastolic BP [DBP] ≥ 90 mmHg) are common (up to 80%) upon hospital admission in patients with acute ischaemic stroke (AIS) or acute ICH.^1^ However, the pathophysiology of acute post-stroke hypertension is poorly understood.^2^^,^^3^ Despite considerable research effort, the optimal management of BP in these conditions remains controversial and unresolved. Because RCTs on this topic are limited and methodologically challenging, clinical decisions are often based on numerous observational studies, which are prone to bias, confounding and random error.^4–7^

Theoretical concepts and pathophysiological arguments have been used to support both lowering and maintaining elevated BP in the acute stroke setting to improve clinical outcomes. These include: (1) reducing the risk of stroke recurrence, cerebral oedema and reperfusion haemorrhage in AIS patients treated with intravenous thrombolysis (IVT) and/or mechanical thrombectomy (MT); (2) limiting haematoma expansion and cerebral oedema in ICH; (3) avoiding compromise of cerebral perfusion to viable ischaemic tissue in the presence of altered autoregulation.^2^^,^^8–10^ Whilst most attention has focused on avoiding hypertension, drug-induced hypertension has been proposed as a potential therapeutic strategy to increase cerebral perfusion in selected AIS patients undergoing reperfusion therapies.^11^^,^^12^

Although numerous observational studies have shown that both extremely low and high BP levels are associated with worse outcomes in AIS and ICH patients,^13–16^ results from multiple RCTs investigating different antihypertensive strategies across acute stroke subtypes have been inconsistent. In addition, randomised evidence on BP lowering in patients with LVO, successfully treated with MT appears to contradict observational data suggesting that elevated BP levels adversely affect outcomes following successful reperfusion.^17^^,^^18^

Since the publication of the 2021 ESO guidelines on acute BP management in AIS and ICH,^19^ several new RCTs and high-quality observational studies have been published, expanding to the evidence base. In light of these developments, we undertook an update of the 2021 ESO guideline on acute BP management in patients with AIS and ICH.^19^

**Table 1:** Disclosures of the module working group (MWG) members.

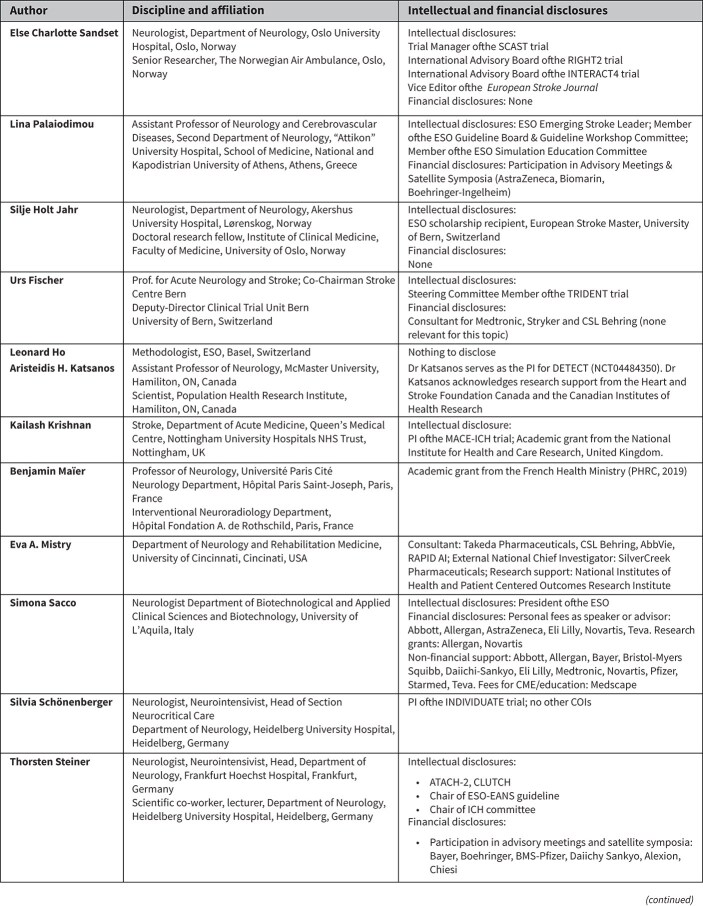
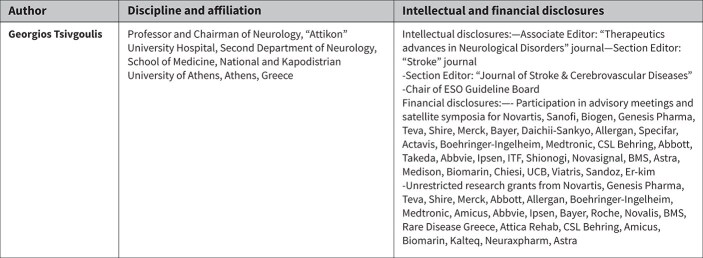

## Methods

This update prioritises the inclusion of the most reliable evidence from RCTs, or, where such evidence is lacking, from systematic reviews of high-quality observational studies.

### Composition and approval of the module working group

This guideline update was initiated by the ESO and prepared according to ESO standard operating procedures, which are based on the Grading of Recommendations, Assessment, Development and Evaluations (GRADE) system. The ESO Guidelines Board and Executive Committee reviewed the intellectual and financial disclosures of the module working group (MWG) members (Table 1) and approved the group’s composition. The MWG was chaired by the first (E.C.S.) and senior (G.T.) authors.

### Development and approval of clinical questions

The MWG followed these steps:

1. Identified and agreed upon a list of clinically relevant topics for guideline users, excluding those related to BP management for secondary stroke prevention, TIA or pediatric acute stroke.
2. Formulated a set of Population, Intervention, Comparator, Outcome (PICO) questions, which were reviewed and approved by the ESO Guidelines Board and Executive Committee.
3. Produced a list of relevant outcomes, which were rated for importance using the Delphi method. Each outcome was scored from 1 to 9 for each PICO question (based on mean score from 10 respondents). The complete list of outcomes and their corresponding Delphi ratings for each PICO question is presented in Table S1. Based on voting scores, functional outcome and mortality were assigned the highest and second highest priorities, respectively, across both AIS- and ICH-related PICO questions. Symptomatic ICH (sICH) ranked as the third most important outcome in most AIS-related PICOs, while haematoma expansion held this position in the ICH-related PICOs. Excellent functional outcome was defined as 90-day mRS scores of 0–1.^20^^,^^21^ Good functional outcome was defined as 90-day mRS scores of 0–2.^20^^,^^21^ Reduced disability was defined as ≥ 1-point reduction in mRS score at 90 days (ordinal shift analysis).^20^^,^^21^
4. The main recommendations were derived from systematic reviews of RCTs evaluating different BP management strategies in AIS and ICH. The literature search was conducted in MEDLINE (Ovid), Embase (Ovid) and the Cochrane Library and was completed on 1 June 2025. To this aim, separate systematic reviews were conducted for each PICO question, resulting in 29 analyses. Details of the search strategy are provided in the Supplement.
5. The authors independently screened titles and abstracts identified through the electronic search and reviewed the full text of potentially eligible RCTs.
6. For each PICO question, a subgroup of 3–4 MWG members was formed. Where no RCT data were available, targeted literature searches identified relevant systematic reviews of non-randomised studies or key observational studies.
7. Where appropriate, random-effects meta-analyses were conducted using Revman online (2024; developed by the Cochrane Collaboration), with results presented as odds ratios (ORs) or common ORs (cORs) with corresponding 95% CIs. Heterogeneity was assessed using the *I*^2^ statistic and classified as moderate (*I*^2^ ≥ 30%), substantial (*I*^2^ ≥ 0%) or considerable (*I*^2^ ≥ 75%).^22^ All analyses were conducted by the ESO methodologist (L.H.) and independently verified by members of the guideline (G.T., E.C.S., L.P., S.J.H.).
8. The results of data analysis were imported into the GRADEPro Guideline Development Tool (2015; developed by Evidence Prime, Inc.). For each PICO question and outcome, risk of bias was assessed using the Cochrane Risk of Bias 2 (RoB 2) tool for RCTs.^23^ The overall quality of evidence was rated as high, moderate, low or very low, based on the study design and factors such as inconsistency, indirectness, imprecision and risk of bias.^20^^,^^24^^,^^25^ Grading of Recommendations, Assessment, Development and Evaluations evidence profiles and summary of findings tables were generated accordingly.
9. Each PICO group addressed its assigned question by drafting up to 3 paragraphs. The first paragraph, entitled “Analysis of current evidence,” summarised relevant pathophysiological considerations and existing recommendations from other scientific societies related to the specific PICO, followed by a synthesis and discussion of results from the relevant RCTs. Where no RCTs were available, findings from systematic reviews of non-randomised studies were presented instead. The second paragraph, “Additional information,” was optional and used to provide more details on RCTs discussed, summarise key observational studies or highlight ongoing or planned RCTs. The third paragraph, “Evidence-based recommendation,” presented a recommendation formulated using the GRADE methodology. The direction, strength and wording of each recommendation were determined according to the GRADE evidence profiles and aligned with the ESO standard operating procedure (Table 2).^20^^,^^24–26^ Where the group judged that evidence was insufficient to support a formal recommendation, an “Expert consensus statement” was included. In such cases, a pragmatic suggestion was provided for clinical practice, along with the voting results from all MWG members (excluding L.H. [methodologist], L.P. and S.H.J. [fellows involved in literature review, data extraction, bias assessment and drafting]). Importantly, the suggestions are not evidence-based recommendations but represent the collective expert opinion of the MWG.

**Table 2:** Formatting based on strength of recommendations.

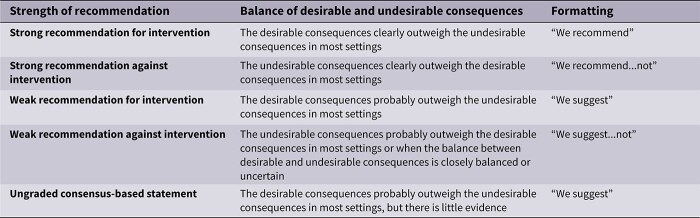

**Figure 1:**
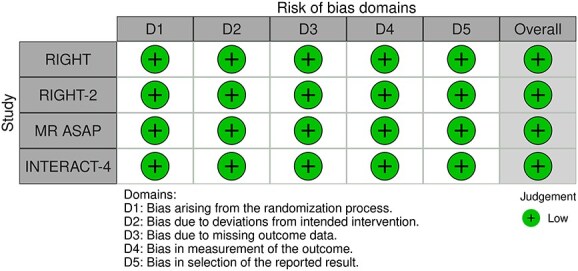
Risk of bias in each randomised controlled trial comparing blood pressure-lowering therapies to control in patients with suspected stroke.

**Figure 2:**
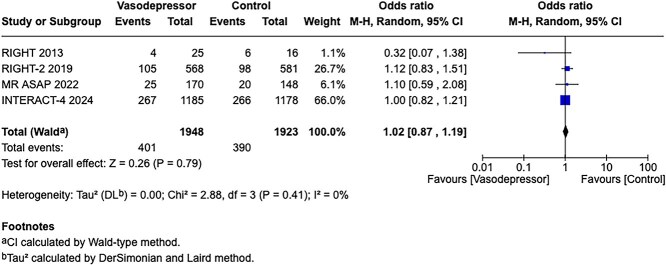
Effect of pre-hospital blood pressure lowering by any vasodepressor drug compared with no drug on mortality in the overall suspected stroke population at 3 months following symptom onset.

All data analyzed or generated in this study are included in this article and its supplementary information files. The full guideline underwent several rounds of review and revision by all MWG members until consensus was reached. Finally, it was reviewed and approved by external reviewers and the ESO Guidelines Board and Executive Committee.

## Results

### PICO 1

In patients with suspected acute stroke, does pre-hospital BP lowering with any drug, compared to no drug, improve outcome?

### Analysis of current evidence

Patients with suspected stroke in the pre-hospital phase commonly present with high BP, which may vary depending on stroke subtype or the presence of stroke mimics.^27^ High BP has been associated with unfavourable short- and long-term functional outcomes in both AIS and ICH.^16^^,^^28^

Lowering BP in the pre-hospital setting has been proposed as a strategy to provide a critical window for early intervention in acute stroke. In ICH, early control of elevated BP aims at improving outcomes by minimising haematoma expansion.^29^^,^^30^ In AIS, however, the optimal approach remains much less clear. Timely management of severe hypertension is nevertheless essential, as BP levels exceeding 185/110 mmHg constitute a contraindication to IVT.^31^

This meta-analysis includes 4 RCTs comparing BP lowering with control (standard care alone or with sham/gauze dressing) initiated in patients with suspected stroke in the ambulance before hospital-based neuroimaging.^32–35^ Collectively, the trials enrolled 3871 patients, with a median time to randomisation ranging from 55 to 71 min after symptom onset. Glyceryl trinitrate (GTN) was the antihypertensive agent used in all trials^32^^,^^33^^,^^35^ except one, which used urapidil.^34^ Together, these trials demonstrated the feasibility of initiating BP-lowering therapy in the pre-hospital setting.

The Rapid Intervention with Glyceryl Trinitrate in Hypertensive stroke (RIGHT) trial was a pilot, single-blind, paramedic-delivered feasibility study conducted in Nottingham, UK.^33^ Of the planned 80 participants, 41 patients with suspected stroke were enrolled. Eligibility criteria included SBP ≥ 140 mmHg within 4 h from symptom onset, a FAST score of 2 or 3, age > 40 years for men or > 55 for women and no contraindications to GTN. Exclusion criteria included reduced consciousness (GCS ≤ 8), hypoglycaemia or pre-stroke non-ambulatory status. Participants received either transdermal GTN (5 mg/24 h) or no GTN (control) for 7 days, starting in the ambulance. The primary outcome, SBP at 2 h, was significantly lower in the GTN group (153 mmHg vs 174 mmHg; *P* = .030). Secondary outcomes included haemodynamic measures, feasibility and 90-day functional outcomes. Glyceryl trinitrate was associated with better mRS scores (*P* = .040), without significant differences in death or serious adverse events.

The pre-hospital transdermal GTN in patients with ultra-acute presumed stroke (RIGHT-2) trial was a large, multicentre study conducted across 8 UK ambulance services, using a paramedic-delivered, sham-controlled, blinded end point design.^32^ A total of 1149 patients with presumed stroke, SBP ≥ 120 mmHg and a FAST score of 2 or 3 within 4 h of symptom onset were enrolled. Key exclusions were reduced consciousness (GCS < 8), hypoglycaemia, seizures or nursing-home residence. Participants were randomised to receive either transdermal GTN (5 mg daily for 4 days) or a sham dressing, initiated in the ambulance. The primary outcome was mRS at 90 days. While GTN modestly reduced BP, no significant difference in mRS was found between the GTN and sham groups, either in the confirmed stroke or TIA (adjusted common OR 1.25; 95%CI, 0.97–1.60; *P* = .083) or in the full intention-to-treat population (OR 1.04; 95%CI, 0.84–1.29; *P* = .69). Secondary outcomes, including death, neurological deterioration and quality of life, were similar between groups. Notably, there was a signal of potential harm in patients with ICH, with a trend towards worse 90-day functional outcome in the GTN group (adjusted OR 1.87; 95%CI, 0.98–3.57; *P* = .058) and higher in-hospital mortality (adjusted OR 2.26; 95%CI, 1.03–4.95), although overall 90-day mortality did not differ significantly.^36^ In RIGHT-2, GTN-treated patients with ICH exhibited a greater frequency of absolute haematoma expansion (46% vs 27%; OR 2.27; 95%CI, 0.90–5.74), providing a plausible mechanism for the trend towards poorer functional outcome.^36^

The pre-hospital transdermal GTN in patients with presumed acute stroke (Multicentre Randomised trial of Acute Stroke treatment in the Ambulance with a nitroglycerin Patch [MR ASAP]) trial was a phase 3, open-label study with blinded end point assessment, conducted by paramedics across 6 regional ambulance services in the Netherlands.^35^ Inclusion criteria were probable stroke, FAST score 2 or 3, SBP ≥ 140 mmHg and symptom onset within 3 h. Participants were excluded if they had significant pre-stroke dependency in activities of daily living, reduced consciousness (GCS < 8) or known contraindication or hypersensitivity to GTN. Participants were randomised in the ambulance to receive either transdermal GTN (5 mg/24 h) plus standard care or standard care alone. A total of 325 patients were included in the modified intention-to-treat analysis before the trial was terminated early. As MR ASAP used a deferred-consent design, patients who did not provide consent were not included in the analysed cohort; among participants in the modified intention-to-treat population, loss to follow-up was < 5%, minimising the risk of bias from missing outcome data. The primary outcome, 90-day functional outcomes (mRS), showed no significant difference between groups (adjusted common OR 0.97; 95%CI, 0.65–1.47). Among patients with ICH, however, early (7-day) mortality was notably higher in the GTN group (34% vs 10%, adjusted OR 5.91; 95%CI, 0.78–44.81), raising concerns about potential harm. Although this did not translate into a significant difference in 90-day mortality, the safety signal led to premature trial termination.

The INTEnsive ambulance-delivered blood pressure Reduction in hyper-ACute stroke Trial (INTERACT4) was an open-label trial with blinded end-point assessment, conducted across 51 sites in China.^34^ The study enrolled 2303 hypertensive patients (SBP ≥ 150 mmHg) with suspected acute stroke, defined by a FAST score of 2 or 3 and an arm motor deficit, within 2 h of last known well. Participants were randomised to receive either intensive BP lowering, with intravenous urapidil (25 mg, repeated once after 5 min if necessary) to achieve a target SBP of 130–140 mmHg within 30 min and maintained for 7 days or until discharge, or to standard BP management according to local guidelines. Exclusion criteria included coma (GCS < 5), severe comorbidities, epilepsy, recent head injury or hypoglycaemia. The primary outcome, 90-day functional outcomes (mRS), showed no difference between groups in the overall population (common OR 1.00; 95%CI, 0.87–1.15). However, subgroup analyses indicated a potential benefit of intensive BP lowering in patients with ICH (common OR 0.75; 95%CI, 0.60–0.92) and a possible harm in those with ischaemic stroke (common OR 1.30; 95%CI, 1.06–1.60). Additionally, haematoma expansion within 24 h was reduced in the intensive treatment group compared with standard care (absolute haematoma expansion 11.5% vs 17.3%; OR 0.65; 95%CI, 0.42–1.00 and relative haematoma expansion 15.1% vs 20.5%; OR 0.64; 95%CI, 0.43–0.96). The incidence of serious adverse events was similar between groups.

The Paramedic Initiated Lisinopril for Acute Stroke Treatment (PIL-FAST) trial randomised 14 patients with unilateral arm weakness and SBP ≥ 160 mmHg within 3 h of symptom onset to sublingual lisinopril or control.^37^ Admission BP was lower in the lisinopril group, but 1-week mortality was similar (17% vs 13%). As mortality beyond 1 week was not reported, the trial was excluded from the meta-analysis.

The MWG’s assessment of risk of bias for each RCT, based on the Cochrane RoB 2 tool, is shown in Figure 1. Overall, the included studies were judged to have a low risk of bias. Table 3 presents the detailed assessment of the quality of evidence for all outcomes evaluated in PICO 1.

**Table 3:** GRADE evidence profile for PICO 1: In patients with suspected acute stroke, does pre-hospital blood pressure lowering with any drug compared to no drug improve outcome?

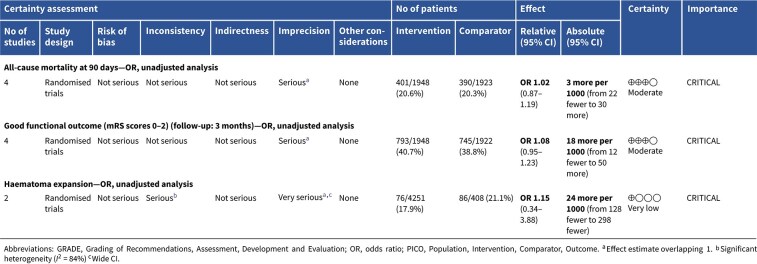

**Figure 3:**
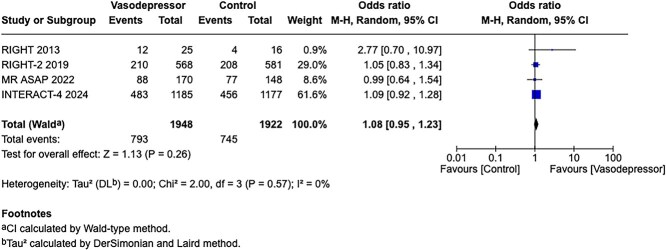
Effect of pre-hospital blood pressure lowering by any vasodepressor drug compared with no treatment on good functional outcome (mRS scores 0–2) in the overall suspected stroke population at 3 months following symptom onset.

Among the overall population of patients with suspected stroke, pre-hospital BP lowering with vasodepressor agents, compared with control, was not associated with a significant difference in 3-month mortality (OR 1.02; 95%CI, 0.87–1.19; *P* = .79; *I*^2^ = 0%; Figure 2). Similarly, no improvement in functional outcome at 3 months was observed (OR 1.08; 95%CI, 0.95–1.23; *P* = .26; *I*^2^ = 0%; Figure 3).

**Figure 4:**
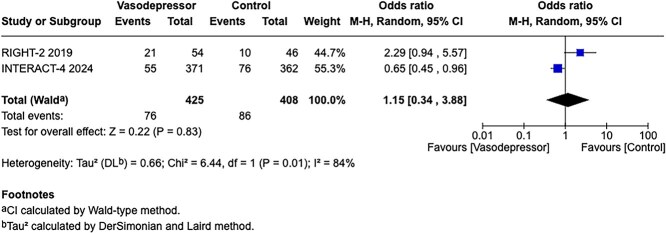
Effect of pre-hospital blood pressure lowering by any vasodepressor drug compared with no treatment on haematoma expansion in patients with intracerebral haematoma.

In patients with ICH, pre-hospital BP lowering was not associated with a significant difference in haematoma expansion (OR 1.15; 95%CI, 0.34–3.88; *P* = .83; Figure 4). The contrasting results between the 2 included RCTs may be explained by differences in the interventions tested: RIGHT-2 used a fixed-dose GTN patch with non-titrated BP reduction,^32^ while INTERACT4 implemented a controlled BP-target strategy achieved with titrated urapidil.^34^

### Additional information

Although pre-hospital BP lowering showed no effect in the overall population with suspected stroke, subgroup analyses suggest differential effects depending on stroke subtype, particularly between AIS and ICH.

In ischaemic stroke, the INTERACT4 trial demonstrated an association between pre-hospital BP lowering and worse 90-day functional outcomes (adjusted common OR 1.30; 95%CI, 1.06–1.60).^34^ Similarly, RIGHT-2 reported a non-significant trend towards poorer outcomes among treated ischaemic stroke patients (adjusted common OR 1.15; 95%CI, 0.85–1.54; *P* = .36).^32^ Multicentre Randomised trial of Acute Stroke treatment in the Ambulance with a nitroglycerin Patch provided weak but suggestive evidence of potential harm from GTN in ischaemic stroke (OR 0.67; 95%CI, 0.39–1.13; *P* for interaction = .06).^35^

In contrast, INTERACT4 suggested potential benefit in ICH, showing reduced haematoma expansion (OR 0.65; 95%CI, 0.45–0.96) and improved 90-day functional outcome (common OR for disability worsening 0.75; 95%CI, 0.60–0.92).^34^ However, RIGHT-2 and MR ASAP raised concerns regarding transdermal GTN use in ICH. In RIGHT-2, GTN was associated with significantly larger haematoma volumes (*P* = .030), increased mass effect (*P* = .0083) and a trend towards worse 90-day functional outcomes (adjusted OR 1.87; 95%CI, 0.98–3.57; *P* = .057).^32^^,^^36^ In MR ASAP, higher early (7-day) mortality was observed in the GTN group (OR 5.91; 95%CI, 0.78–44.81), although no difference in 90-day functional outcome was detected, and no significant differences in haematoma volumes were reported.^35^ These findings primarily reflect 2 drug classes: nitric-oxide donors delivered as transdermal GTN in RIGHT,^33^ RIGHT-2^32^ and MR ASAP,^35^ and the α1-adrenergic antagonist urapidil used in INTERACT4^34^; no other antihypertensive agent has been studied in pre-hospital ICH RCTs. Given this limited evidence base, agents with predictable, titratable haemodynamic effects and extensive in-hospital use, such as urapidil and labetalol, could be favoured when pre-hospital BP lowering is considered necessary. A summary of the antihypertensive medications and BP-lowering strategies employed across all included studies can be found in Table S2.

### PICO 2

In hospitalised patients with AIS not treated with reperfusion therapies (IVT or MT), does BP lowering with any vasodepressor drug, compared to no drug, improve outcome?

### Analysis of current evidence

Only a minority of patients with AIS receive reperfusion therapy in Europe, with the proportion ranging from 1% to 20% (mean 7.3%) for intravenous alteplase and even fewer (mean 1.9%) for MT.^38^ From a pathophysiological perspective, optimal BP management may differ in patients ineligible for reperfusion therapies such as IVT or MT. While BP lowering could reduce the risk of haemorrhagic transformation and cerebral oedema, elevated BP may help preserve cerebral perfusion in the context of impaired autoregulation.

Since the publication of the 2021 ESO Guideline, no new clinical trials have been identified. Therefore, this analysis of BP-lowering treatment on death and/or disability in patients with AIS not treated with reperfusion therapies is based on data from 19 existing RCTs.^32^^,^^39–57^ Seven studies included both AIS and ICH but reported results separately for the 2 subgroups.^32^^,^^40^^,^^43^^,^^51–54^^,^^57^ One trial did not distinguish between AIS and ICH but was included under the assumption that AIS comprised the majority of cases.^41^ Three trials included patients treated with IVT, although the proportions were low; subgroup results based on IVT status were available from the main publication or post hoc analyses.^32^^,^^40^^,^^53^ Most studies excluded patients with extremely high SBP (>220 mmHg), and many included patients up to 72 h after symptom onset.

Most participants were recruited from the 3 large RCTs described below. The Scandinavian Candesartan Acute Stroke Trial (SCAST) recruited 2029 patients with AIS (*n* = 1733) and ICH (*n* = 274) with SBP ≥ 140 mmHg within 30 h of onset, comparing candesartan for 7 days with placebo on co-primary end points at 6 months.^53^ Eight percent of patients with AIS received IVT. At day 7, BP was lower in the treatment group (−5/−2 mmHg difference in SBP and DBP). There were neutral effects on the 2 co-primary endpoints: (1) poor functional outcome at 6 months (ordinal shift on mRS) and (2) composite outcome of vascular death, myocardial infarction or stroke during the 6-month follow-up period. There were no significant differences between the candesartan and placebo groups in mortality, functional or recurrent ischaemic stroke in the subgroup of 1733 patients with AIS.^54^

**Figure 5:**
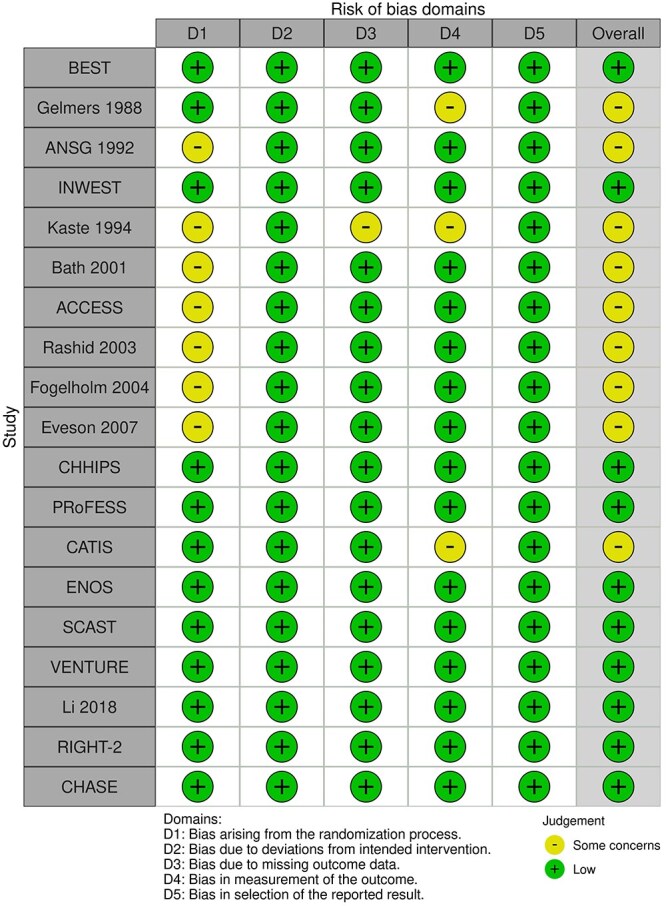
Risk of bias in each RCT of blood pressure-lowering therapies compared to control in hospitalised patients with acute ischaemic stroke not treated with reperfusion therapies.

The Efficacy of Nitric Oxide in Stroke trial (ENOS) randomised 4011 patients with AIS (*n* = 3342; 225 received IVT), or ICH (*n* = 629) and SBP 140–220 mmHg to transdermal GTN (5 mg) or placebo within 48 h of onset for 7 days.^40^ In a partial factorial design, patients on pre-existing antihypertensive therapy were randomised to stop or continue their usual medication. Blood pressure was significantly lower in the GTN treatment group at 24 h (−7/−3 mmHg difference), but not after day 3. In the overall population (AIS and ICH combined), the primary outcome (worse outcome on mRS scores at 90 days, shift analysis) was neutral (adjusted common OR 1.01; 95%CI, 0.91–1.13; *P* = .83). There was no significant interaction between stroke type (AIS vs ICH) and treatment effect on outcome in a pre-specified subgroup analysis.

The China Antihypertensive Trial in Acute Ischaemic Stroke (CATIS) enrolled 4071 patients with AIS (not treated with IVT) and SBP 140–220 mmHg within 48 h of onset.^47^ The trial compared targeted BP lowering (10%–25% SBP reduction within 24 h) using intravenous angiotensin inhibitors (ACEi) (first line), oral calcium-channel antagonists (or blockers) (CCBs) (second line) or oral diuretics, vs control. Mean SBP was lower (9.1 mmHg at 24 h) in the treatment group. There was no significant difference in functional outcome (mRS ≥ 3) at either 14 days or at 90 days.

All the other included studies reported similarly neutral results. The MWG’s assessment of risk of bias for each RCT, based on the Cochrane RoB 2 tool, is shown in Figure 5. Overall, the included studies were judged to have a low risk of bias. Table 4 presents a detailed assessment of the quality of evidence for all outcomes evaluated in PICO 2.

In analyses of RIGHT-2 limited to 580 patients with AIS, there was no evidence of an effect of GTN on 90-day functional outcome compared with sham (worse outcome on mRS scores at 90 days, shift analysis; adjusted common OR 1.15; 95%CI, 0.85–1.54; *P* = .36).^32^

A post hoc subgroup analysis of the Prevention Regimen for Effectively Avoiding Second Strokes (PRoFESS) trial examined the effect of adding the angiotensin receptor blocker (ARB) telmisartan to standard antihypertensive treatment vs placebo in 1360 patients with mild AIS within 72 h of onset.^42^ Telmisartan produced a modest reduction in BP without an increase in adverse events, but did not affect functional outcome, mortality, stroke recurrence or cardiovascular events. This cohort had mild neurological severity [median NIHSS score 3], and late treatment initiation (mean 58 h after onset).

In a comparison of amlodipine or irbesartan vs control in 320 patients with AIS (<48 h of onset), results for poor functional outcome (mRS ≥ 3) favoured treatment (32.1% in the treatment group vs 45.0% in the control group, *P* = .018).^49^ Conversely, the Intravenous Nimodipine West European Stroke Trial (INWEST), comparing the effect of nimodipine (1 or 2 mg IV for 5 days followed by 120 mg orally) with placebo in 265 patients with AIS, reported poorer functional outcomes (Barthel Index) and neurological scores (Orgogozo scales) in the 2 mg treatment group vs placebo at 21 days (primary efficacy time point) and 24 weeks.^56^

**Figure 6:**
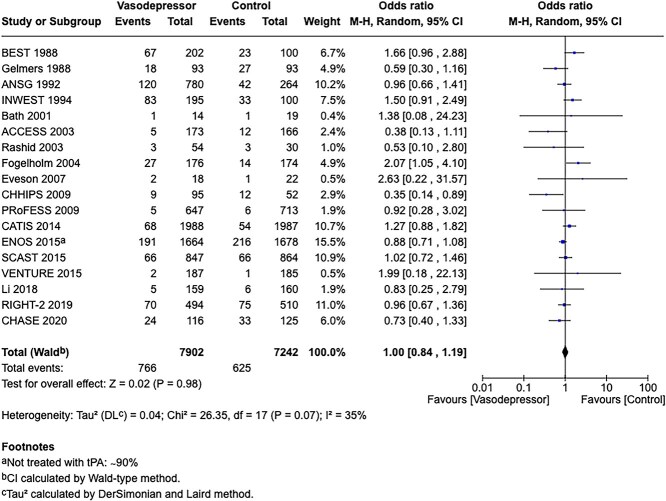
The effect of blood pressure lowering with any vasodepressor drug compared with no drug on mortality at 3–6 months following symptom onset in patients with acute ischaemic stroke not treated with acute reperfusion therapies.

In total, 18 RCTs were included in the meta-analysis assessing the effect of BP reduction on mortality 3–6 months after symptom onset.^32^^,^^39–44^^,^^46–57^ No statistically significant effect was observed (OR 1.01; 95%CI, 0.86–1.18; *P* = .93; Figure 6).

**Figure 7:**
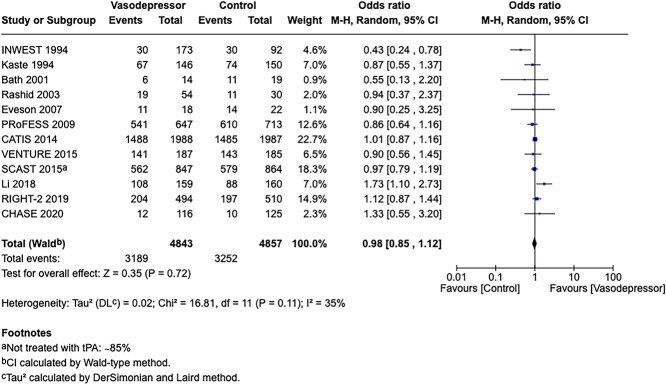
The effect of blood pressure lowering with any vasodepressor drug compared with no drug on good functional outcome (mRS scores 0–2) at 3–6 months following symptom onset in patients with acute ischaemic stroke not treated with acute reperfusion therapies.

Twelve studies evaluated the impact of BP lowering on good functional outcome, defined as an mRS score of 0–2 at 3–6 months post-onset.^32^^,^^42–44^^,^^47–50^^,^^52^^,^^53^^,^^57^^,^^58^ Similarly, no significant difference was found between treatment with any vasodepressor agent and control (OR 0.96; 95%CI, 0.85–1.08; *P* = .49; Figure 7). Only 1 RCT (the Valsartan Efficacy oN modesT blood pressure Reduction in acute ischaemic stroke [VENTURE]) specifically evaluated the impact of BP reduction on sICH.^50^ However, the study was underpowered, and the number of sICH events was too small to allow for any definitive conclusions. Table 4 provides details regarding the GRADE assessment of the quality of evidence for all outcomes evaluated in PICO 2.

### Additional information

We hypothesise that factors other than BP itself may influence the treatment effect on clinical outcomes. These include drug class, BP target, timing of treatment, underlying stroke aetiology, specific comorbid conditions (eg, myocardial ischaemia, acute heart failure or other hypertensive emergencies), duration and severity of baseline hypertension and the magnitude as well as rate of BP lowering. Data from the included RCTs regarding these variables are inconsistent. When considering drug class: ACEis appear safe but have not influenced outcomes^47^^,^^51^; RCTs using ARBs for BP reduction report conflicting results.^50^^,^^53^^,^^55^ Candesartan showed promising effects in a pilot trial,^55^ but SCAST was neutral and, if anything, favoured placebo.^53^ The VENTURE trial was neutral for functional outcome but reported significantly more early neurological deterioration among patients in the valsartan group.^50^ The low-dose BEta blockade in acute Stroke Trial (BEST) reported an increased early mortality in those randomised to beta-blocker vs control (although this was minimal and did not reach significance in adjusted analyses).^41^ In contrast, labetalol was found safe in the Controlling Hypertension and Hypotension Immediately Post Stroke (CHHIPS) trial.^51^ Results from small RCTs of CCBs have been mixed: some favoured placebo,^48^^,^^56^ whereas others were neutral.^39^ Trials of nitric oxide (NO) donors have reported safety with GTN but no significant effect on functional outcome.^36^^,^^40^^,^^43^^,^^52^

**Table 4:** GRADE evidence profile for PICO 2: In hospitalised patients with acute ischaemic stroke not treated with reperfusion therapies (intravenous thrombolysis or mechanical thrombectomy), does blood pressure lowering with any vasodepressor drug, compared to no drug, improve outcome?

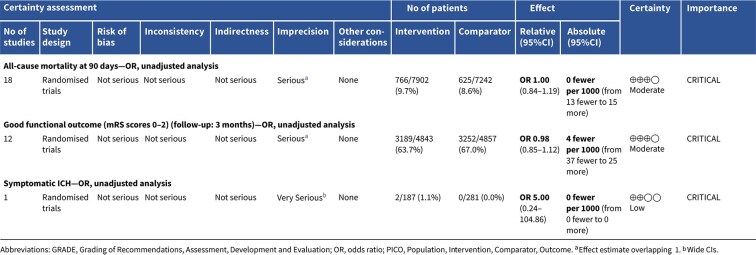
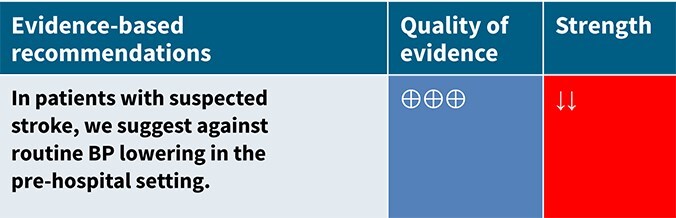
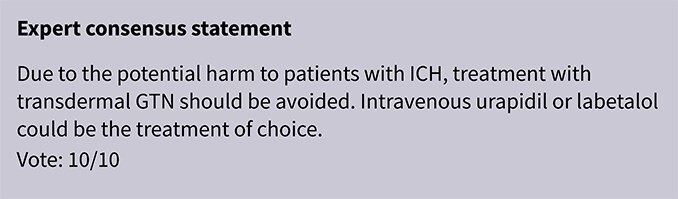

Premorbid and initial BP levels, as well as the chosen BP target, may be of importance.^3^ Most RCTs excluded patients with extremely elevated SBP (>220 mmHg), so the effects of BP lowering in this group remain unknown. Because of heterogeneity in BP targets across studies, no robust conclusions can be drawn regarding the optimal BP target. However, avoiding large reductions in BP within the first 24 h seems reasonable, given the negative effects reported in trials of intravenous CCBs associated with marked BP drops (>20%).^48^^,^^55^^,^^56^^,^^58^ The interval from stroke onset until treatment varied considerably across RCTs (<4 h to 5 days) and may modify treatment effects. This variation is clinically relevant because BP is typically highest and most unstable in the early hours after stroke onset, with spontaneous decline occurring over the subsequent 24–72 h.^28^^,^^59^ As such, any potential effect of BP lowering (whether beneficial or harmful) is most biologically plausible during this early window, whereas later intervention, when haemodynamic parameters have already begun to stabilise, is less likely to influence outcome. This physiological pattern may partly explain the heterogeneity observed across trial subgroups treated at different times. In the ENOS subgroup treated within 6 h, GTN was associated with significantly improved functional outcomes,^40^ while in SCAST, there was a non-significant benefit on the composite vascular endpoint in those treated within 6 h.^60^ In CATIS, subgroup analyses showed a reduction in poor functional outcome among those randomised later (>24 h) after stroke onset.^47^ In RIGHT-2, where treatment was initiated within 4 h, GTN was associated with a non-significant trend towards worse functional outcome.^2^ This tendency towards harm was more evident in patients with very early stroke (<1 h) and severe stroke (Glasgow Coma Scale < 12, NIHSS > 12).^32^

**Table:** 

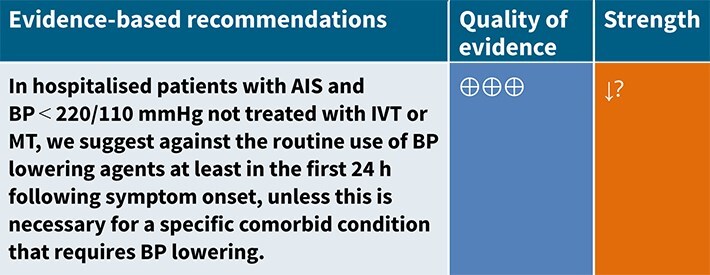

**Table:** 

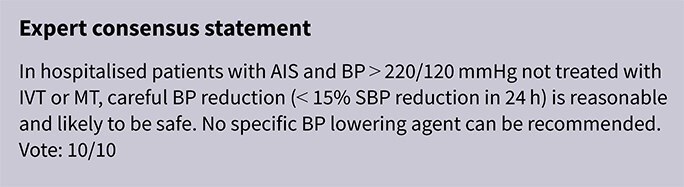

Neurological severity and AIS subtype may modify treatment effects. In a secondary subgroup analysis of SCAST, candesartan treatment showed benefit in larger (total and partial anterior circulation) but not smaller (lacunar) infarcts,^54^ as shown in another study.^58^ The presence of LVO or significant carotid artery stenosis may also influence treatment effect, although data are partly conflicting. In a pre-specified SCAST subgroup with carotid imaging (*n* = 993), patients with severe stenosis (≥70%) treated with candesartan had a trend towards increased stroke progression and worse functional outcome (ordinal shift on the mRS).^61^ Conversely, in ENOS, GTN was shown to be safe across all levels of ipsilateral carotid stenosis in participants with carotid imaging data (*n* = 2038).^62^

We considered a 2014 Cochrane review relevant to this topic.^9^ In the subgroup of 11,015 patients with AIS, there was no benefit of any vasodepressor drug compared with control for death or dependency (as reported in the individual trials): OR = 1.00 (95%CI, 0.92–1.08). No differences in treatment effect were observed across subgroups defined by drug class, stroke location (cortical vs subcortical) or BP target used. Calcium-channel antagonists (blockers), ACEi, ARB, beta-blockers, and NO donors all effectively reduced BP.^9^

### PICO 3

In hospitalised patients with AIS and undergoing IVT (with or without MT), do BP lowering therapies compared to control improve outcome?

### Analysis of current evidence

The recommended BP thresholds for administering IVT in AIS patients—specifically, SBP < 185 mmHg and DBP < 110 mmHg before, and SBP < 180 mmHg and DBP < 105 mmHg during and after administration, respectively—originate from early, non-randomised pilot studies and the original National Institute of Neurological Diseases and Stroke (NINDS) rt-PA trial protocol.^63–66^ These thresholds have been adopted in current American Heart Association/American Stroke Organization (AHA/ASA) and ESO guidelines despite the absence of RCT data supporting these specific thresholds.^19^^,^^31^^,^^67^

The Enhanced Control of Hypertension and Thrombolysis Stroke Study (ENCHANTED) is the most relevant RCT in this context.^68^ This trial compared intensive BP lowering (target SBP 130–140 mmHg within 1 h) with standard management (<180 mmHg) in 2196 AIS patients eligible for IVT.^68^ Although no significant difference was observed in 90-day functional outcomes (unadjusted common OR per 1-point improvement across all mRS scores: 1.01; 95%CI, 0.87–1.17; *P* = .87), the intensive arm showed a modest reduction in any ICH (14.8% vs 18.7%; OR 0.75; 95%CI, 0.60–0.94; *P* = .01).^68^ Type 2 parenchymal haemorrhage was also less frequent, although not statistically significantly (OR 0.71; 95%CI, 0.50–1.01; *P* = .05).^68^ Quality of life scores did not differ between groups.^68^ However, ENCHANTED has limitations: it was open-label with blinded endpoint adjudication; the actual mean BP difference between groups was smaller than planned (<7 mmHg); most participants had pre-randomisation BP < 185/110 mmHg; and the majority were from Asia, potentially limiting generalisability.^69^

A subgroup analysis of the ENOS trial evaluated BP lowering within 48 h of stroke onset among 425 AIS patients with elevated SBP (140–220 mmHg) who received intravenous alteplase.^40^ Patients were randomised to GTN (*n* = 204) or placebo (*n* = 221). Glyceryl trinitrate treatment showed no significant benefit in 3-month functional outcomes compared to placebo (unadjusted common OR for mRS score worsening: 0.93; 95%CI, 0.66–1.30).^40^

An individual patient data meta-analysis (IPDM) by Bath et al., which included IVT-treated patients from the ENOS and RIGHT trials,^33^^,^^40^ evaluated the efficacy of GTN for BP management in acute stroke.^70^ Among 98 AIS patients who received IVT, GTN was associated with significantly reduced BP and improved functional outcomes at 3 months compared to placebo (unadjusted common OR for mRS score worsening: 0.32; 95%CI, 0.15–0.69).^70^ However, these findings were not replicated in the RIGHT-2 trial, although the GTN group showed a non-significant trend towards reduced haemorrhagic transformation (3% vs 8%; OR 0.38, 95%CI, 0.13–1.13; *P* = .082).^32^

The Second Enhanced Control of Hypertension and Thrombectomy Stroke Study (ENCHANTED2-MT) extended the investigation of BP management to patients undergoing MT, many of whom also received IVT.^71^ In this trial, patients were randomised to receive either intensive SBP lowering (target 100–129 mmHg) or a less-intensive approach (target 130–149 mmHg) within 3 h of successful recanalisation).^71^ Among those who received IVT, the adjusted common OR for worse functional outcome (ordinal shift in mRS scores at 90 days; primary outcome of the trial) was 1.24 (95%CI, 0.93–1.67) with intensive SBP lowering.^71^ These results align with previous findings from ENCHANTED, suggesting that intensive BP lowering after reperfusion may reduce haemorrhagic complications without translating into functional benefit, at least in unselected populations.^68^ Limitations such as open-label design and inclusion of primarily Asian participants again raise questions regarding generalisability.^71^

The Blood Pressure Target in Acute Stroke to Reduce haemorrhaGe After Endovascular Therapy (BP-TARGET) trial assessed the effect of intensive vs standard SBP lowering (target 100–129 mmHg vs 130–185 mmHg) within 60 min of successful MT in AIS patients, including a subgroup who also received IVT.^72^ The trial’s primary outcome was the rate of any intracranial haemorrhage on follow-up imaging.^72^ Although subgroup data for the primary outcome were reported for IVT-treated patients (adjusted OR 1.03; 95%CI, 0.55–1.91), the findings primarily relate to the overall incidence of radiographic ICH, representing a surrogate marker and not being considered a guideline-relevant clinical endpoint in this context.^72^ As such, while BP-TARGET contributes to data regarding intensive BP management post-reperfusion, its limited focus on functional outcomes or sICH constrains its applicability to current BP management recommendations in IVT-treated patients.^72^

Among all trials enrolling patients receiving IVT and applying randomised BP management strategies, only ENCHANTED and ENCHANTED2-MT reported extractable data for guideline-relevant clinical outcomes such as functional outcomes.^68^^,^^71^ These 2 trials, therefore, represent the core evidence base for recommendations on BP lowering strategies in AIS patients treated with IVT and are the only RCTs appropriate for meta-analysis.

**Figure 8:**
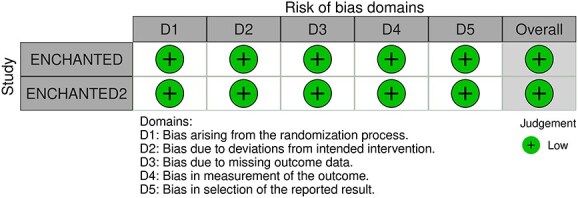
Risk of bias in each RCT of blood pressure-lowering therapies compared to control in hospitalised patients with acute ischaemic stroke and undergoing intravenous thrombolysis (with or without mechanical thrombectomy).

Module working group assessment of the risk of bias in each RCT according to the Cochrane RoB-2 tool is presented in Figure 8. All studies were considered to be at overall low risk of bias.

**Figure 9:**
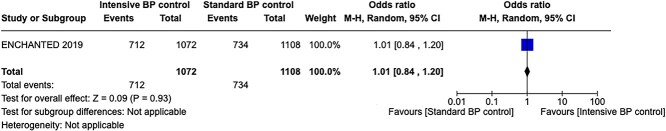
Good functional outcome (mRS 0–2 at 90 days) in patients receiving intensive BP control vs standard BP control (unadjusted pooled odds ratio, random-effects meta-analysis). Abbreviations: BP = blood pressure; M-H = Mantel–Haenszel.

**Figure 10:**
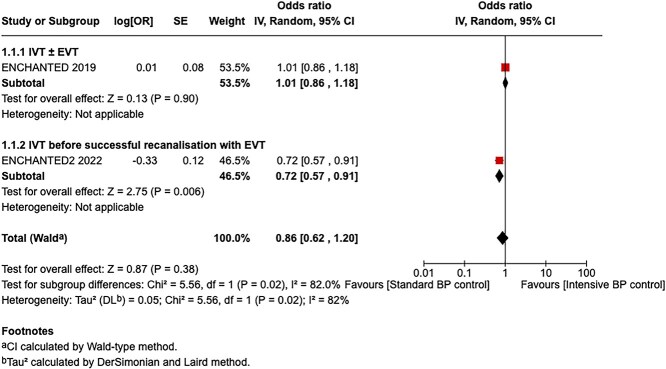
Pooled unadjusted common odds ratio for reduced disability (improvement of a least 1 point on the mRS at 90 days) in patients receiving intensive BP control vs standard BP control (unadjusted pooled common odds ratio, random-effects meta-analysis). Abbreviations: BP = blood pressure; IV = inverse variance.

**Figure 11:**
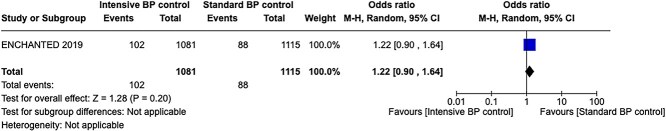
All-cause mortality at 3 months in patients receiving intensive BP control vs standard BP control (unadjusted pooled odds ratio, random-effects meta-analysis). Abbreviations: BP = blood pressure; M-H = Mantel–Haenszel.

**Figure 12:**
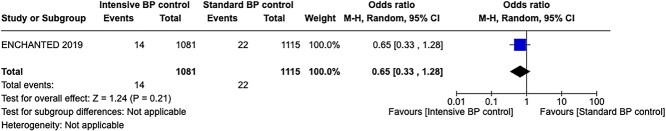
Symptomatic ICH in patients receiving intensive BP control vs standard BP control (unadjusted pooled odds ratio, random-effects meta-analysis). Abbreviations: BP = blood pressure; M-H: Mantel–Haenszel.

Compared to patients randomised to standard BP lowering, the pooled unadjusted OR for good functional outcome in patients randomised to intensive BP control was 1.01 (95%CI, 0.84–1.20; *P* = .93; Figure 9). The unadjusted common OR for reduced disability with intensive BP control was 0.86 (95%CI, 0.62–1.20; *P* = .38; Figure 10). All-cause mortality at 3 months did not differ between the 2 groups (OR 1.22; 95%CI, 0.90–1.64; *P* = .20; Figure 11). Finally, the odds of sICH did not differ between standard vs intensive BP control (OR 0.65; 95%CI, 0.33–1.28; *P* = .21; Figure 12). Table 5 provides details regarding the assessment of the quality of evidence for all outcomes evaluated in PICO 3.

### Additional information

Elevated BP is common in AIS and may lead to delays or exclusions from IVT when antihypertensive agents fail to achieve these thresholds.^1^^,^^73^ Importantly, overly aggressive BP reduction may compromise perfusion in the ischaemic penumbra, potentially exacerbating infarct size and worsening outcomes.^59^^,^^73^^,^^74^ On the other hand, observational studies and meta-analyses suggest that higher BP levels before or during IVT are associated with an increased risk of sICH, reduced likelihood of successful recanalisation and poorer functional outcomes.^75–80^ Post hoc analyses of the second European Cooperative Acute Stroke Study (ECASS II) and the third International Stroke Trial (IST-3) indicate that each 10 mmHg rise in baseline SBP is linked to a greater risk of haemorrhagic complications.^81^^,^^82^ These findings support a linear relationship between elevated BP and both haemorrhage and disability or death.^81^^,^^82^ Further real-world data also suggest that maintaining BP below the conventional thresholds (ie, < 180/105 mmHg) during or after IVT may reduce the incidence of sICH and improve outcomes.^75^^,^^83^^,^^84^ However, no randomised data exist for managing patients whose BP exceeds these thresholds, and evidence for the optimal antihypertensive regimen remains unclear. Importantly, study heterogeneity (AIS subtype, comorbidities and antihypertensive class used) and lack of standardisation in BP management limit definitive conclusions.^2^ No single class of antihypertensive agent has proven superior for managing elevated BP around IVT administration.

Observational studies report that BP protocol violations are frequent—up to 12% of IVT-treated AIS patients have pre-treatment BP above 185/110 mmHg—and these violations correlate with worse outcomes, including higher sICH risk.^75^^,^^85–87^ The Safe Implementation of Treatments in Stroke (SITS) registry analysis further supports that among various off-label IVT criteria, elevated pre-treatment BP is the only one independently associated with increased sICH risk.^85^ Similarly, a phase 3 RCT on sonothrombolysis demonstrated that BP excursions beyond protocol thresholds were linked to worse neurological and imaging outcomes.^86^ In addition, SITS registry data indicated that elevated admission DBP levels were associated with worse functional outcomes among AIS patients with sICH complicating IVT^88^; however, no randomised data exist to guide the management of isolated DBP elevation in this setting.

In patients otherwise eligible for IVT whose BP exceeds the recommended treatment thresholds, active BP lowering is routinely performed in clinical practice to allow IVT administration rather than excluding patients from reperfusion therapy. This approach is supported by observational data and widespread clinical experience,^80^ although there is currently no randomised evidence demonstrating that BP lowering in this specific context independently improves clinical outcomes. As such, BP reduction prior to IVT should be viewed as a pragmatic strategy to enable reperfusion therapy, rather than as an intervention with proven benefit in its own right. The balance of potential benefit and harm from BP lowering in this setting remains uncertain, underscoring the need for further randomised trials.

**Table 5:** GRADE evidence profile for PICO 3: In hospitalised patients with acute ischaemic stroke and undergoing intravenous thrombolysis (with or without mechanical thrombectomy), do blood pressure-lowering therapies compared to control improve outcome?

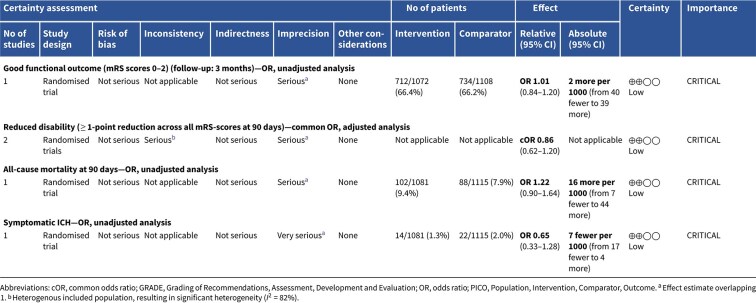

More recently, the Thrombolysis and Uncontrolled Hypertension (TRUTH) study evaluated real-world outcomes of BP management strategies in AIS patients presenting with BP above the thrombolysis threshold of 185/110 mmHg.^89^ This prospective, observational, cluster-based study across 37 Dutch stroke centres compared an active BP-lowering strategy using intravenous antihypertensives with a non-lowering strategy that deferred treatment.^89^ Among 1052 patients (853 in the active group, 199 in the non-lowering group), the adjusted OR for worse 90-day functional outcome with BP lowering was 1.27 (95%CI, 0.96–1.68), indicating no statistically significant benefit.^89^ sICH occurred in 5% of patients in the BP-lowering group vs 3% in the non-lowering group (aOR 1.28, 95%CI, 0.62–2.62).^89^ Importantly, thrombolysis rates (94% vs 52%) and door-to-needle times were more favourable in the BP-lowering group.^89^ However, this study was prematurely terminated due to slow recruitment and limited funding.^89^ Furthermore, its non-randomised design, centre-level treatment allocation and potential residual confounding limit causal interpretation.^89^ Nevertheless, TRUTH highlights the ongoing uncertainty around BP lowering in this context and supports the need for RCTs.

**Table:** 

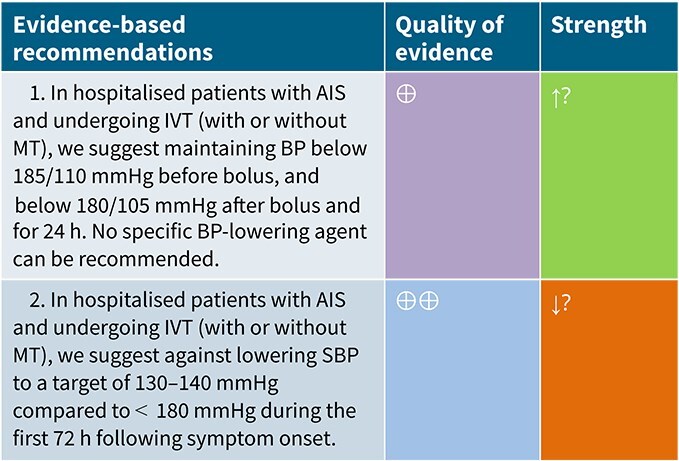

Subgroup analyses from the ENCHANTED trial found no significant heterogeneity in the treatment effect on 3-month functional outcomes (mRS shift) across various patient characteristics, including age, sex, ethnicity, pre-treatment with antiplatelets, alteplase dose (standard vs low), baseline stroke severity (NIHSS) and stroke subtypes (large vessel atherosclerosis, cardioembolism or lacunar stroke).^68^^,^^90^ In a pre-specified subgroup of patients with severe stroke—defined by LVO on angiography, receipt of MT, large artery atherosclerosis or NIHSS > 10—there was no difference in the primary outcome of death or disability between treatment arms.^91^ Notably, however, intensive BP lowering was associated with a significantly higher 3-month mortality (OR 1.52; 95%CI, 1.09–2.13; *P* = .014) compared with guideline-based management, despite a lower rate of clinician-reported ICH (OR 0.63; 95%CI, 0.43–0.92; *P* = .016).^91^ These post hoc findings are hypothesis-generating and highlight the need for further validation in future RCTs.

In addition to absolute BP levels, increasing attention has been directed towards BP variability (BPV) as a potential determinant of outcomes in IVT-treated patients.^2^^,^^92^^,^^93^ Although observational data and meta-analyses have linked higher BPV to worse functional outcomes, greater infarct growth and increased risk of sICH, findings remain inconsistent.^92^ Notably, no RCTs to date have evaluated BPV as a prespecified endpoint. Given its potential prognostic relevance, future trials should consider incorporating BPV analyses to refine individualised BP management strategies in the thrombolysis setting.

### PICO 4

In patients with AIS caused by LVO and undergoing MT (with or without IVT), does BP lowering with any vasodepressor drug compared to no drug improve functional outcome?

### Analysis of current evidence

Although MT has become the standard of care for AIS caused by LVO,^94–97^ nearly 46% of patients with successful reperfusion still experience death or significant disability.^98^ Post-procedural BP management has emerged as a potentially modifiable factor influencing outcomes in this population. However, the optimal BP targets before, during or after MT remain uncertain.^99^ In most RCTs enrolling LVO patients within 6 h of onset, IVT was administered before MT and BP was managed per local protocols targeting ≤ 180/105 mmHg during and 24 h post-procedure.

Current AHA/ASA guidelines recommend maintaining SBP ≤ 180 mmHg and DBP ≤ 105 mmHg during and for 24 h after MT.^67^ These thresholds are largely extrapolated from IVT protocols and aim to reduce the risk of reperfusion-related haemorrhage. Similarly, the joint guidelines from the ESO and the European Society for Minimally Invasive Neurological Therapy (ESMINT) advise keeping BP below 180/105 mmHg during and after MT, while also stressing the importance of avoiding intra-procedural hypotension.^100^ Neither set of guidelines recommends specific antihypertensive agents. Notably, both American^67^ and European^100^ recommendations assign a weak strength of recommendation based on low to very low-quality evidence. In addition, the Society for Neuroscience in Anesthesiology and Critical Care (SNACC) Expert Consensus Statement suggests maintaining SBP between 140 and 180 mmHg and DBP below 105 mmHg during endovascular treatment, regardless of IVT status, using fluids or vasopressors as needed (Class IIa, Level of Evidence B).^101^

In a single-centre pilot RCT, 51 patients with AIS undergoing endovascular thrombectomy were randomised to either a standard intraoperative SBP target of 130–150 mmHg or an augmented target of 160–180 mmHg.^102^ The study demonstrated the feasibility of implementing SBP targets during the procedure, with good adherence and no major safety concerns. However, there were no significant differences in neurological or functional outcomes between the groups. Key limitations include the small sample size, short follow-up duration, lack of blinding and single-centre design, all of which limit statistical power and generalisability. The findings support the feasibility of future larger trials but provide limited guidance on clinical efficacy.

The Individualized Blood Pressure Management During Endovascular Stroke Treatment (INDIVIDUATE) trial was a single-centre, prospective, randomised, open-label, blinded-endpoint (PROBE) study conducted in Germany, investigating whether individualised SBP management during MT for anterior circulation AIS improves clinical outcomes compared to standardised BP targets.^103^ In this trial, 250 patients undergoing MT under procedural sedation were randomised to either individualised BP management or standard management. The intervention group was the individualised approach, which was maintaining the intraprocedural SBP at the level on presentation (±10 mmHg). In the control group, intraprocedural SBP target range was 140–180 mmHg.The primary outcome—good functional outcome at 90 days (mRS scores of 0–2)—was similar between groups (25% individualised vs 24% standard; adjusted OR 0.81; 95%CI, 0.41–1.61; *P* = .56). Although the study was rigorously designed, it was limited by its single-centre nature and limited power to detect modest treatment effects.

The Effect of Individualized Versus Standard BP Management During MT for Anterior Ischaemic Stroke (DETERMINE) trial was a multicentre RCT with PROBE design conducted in France, evaluating individualised BP management during MT.^104^ In the intervention arm, patients received individualised treatment aiming to maintain mean arterial pressure (MAP) within 10% of their initial pre-procedural MAP. The control group targeted an intraprocedural SBP range of 140–180 mmHg. The primary outcome was good functional outcome at 90 days, defined as mRS scores of 0–2. Secondary outcomes include early neurological improvement, excellent functional outcome (defined as mRS score of 0–1), infarct volume, systemic physiological parameters, rates of sICH and 3-month mortality. The results of this trial were presented at the ESO Conference (ESOC) 2025. In total, 433 patients were randomised: 218 in the standard BP group and 215 patients in the individualised BP group. The primary outcome was similar between the groups (44.2% in the individualised BP group vs 48.8% in the standard BP group; adjusted OR 0.82; 95%CI, 0.54–1.24). There was also no difference in any of the secondary outcomes.

Since the publication of the ESO guidelines on BP management in acute stroke in 2021,^19^ 5 RCTs evaluating different BP targets below the pre-specified cut-off of 180/105 mmHg after the end of MT have been published.

The BP-TARGET trial was a multicentre, prospective, open-label RCT with blinded endpoint assessment conducted in France.^72^ This study enrolled AIS patients with anterior circulation LVO who achieved successful reperfusion (modified treatment in cerebral infarction [mTICI] score of 2b-3) following MT. Participants were randomly assigned (1:1) to either an intensive SBP target (100–129 mmHg) or a conservative target (130–185 mmHg) during the first 24 h post-procedure. The primary efficacy outcome was the incidence of radiographic intraparenchymal haemorrhage at 24–36 h, while the primary safety outcome was hypotension. Secondary outcomes included sICH, 90-day mRS distribution, rates of good functional outcome (90-day mRS scores of 0–2), functional improvement (90-day decrease by 1 point across all 90-day mRS grades), infarct volume on follow-up CT, NIHSS change at 24 h and 90-day all-cause mortality. A total of 318 patients were randomised (158 intensive, 160 conservative arm), with similar rates of any ICH (42% vs 43% in the intesnive and conservative arms, respectively) and hypotension (8% vs 3%) across groups. No significant differences were observed in secondary outcomes, including functional improvement (common OR 0.86; 95%CI, 0.57–1.28), good functional outcome (44% vs 45%) or mortality (19% vs 14%). Methodological limitations of the trial included a modest between-group BP difference (~10 mmHg), a substantial proportion of conservative group patients with SBP < 130 mmHg, moderate sample size, use of an imaging rather than clinical primary endpoint and reliance on non-invasive BP measurements at infrequent intervals (every 15 min for 2 h, 30 min for 6 h and hourly for the remaining 16 h).

The ENCHANTED2-MT trial was an open-label, blinded-endpoint, multicentre RCT conducted across 44 hospitals in China to evaluate BP targets following successful MT for AIS.^71^ A total of 821 patients with persistent SBP ≥ 140 mmHg after reperfusion were randomised to receive either intensive BP lowering (<120 mmHg) or less intensive treatment (140–180 mmHg), initiated within 1 h following successful reperfusion after MT and sustained for 72 h. The trial was terminated early due to safety concerns. Patients in the intensive treatment group had significantly worse functional outcomes at 90 days (common OR 1.37; 95%CI, 1.07–1.76), along with higher rates of early neurological deterioration (common OR 1.53; 95%CI, 1.18–1.97) and major disability (mRS scores of 3–5; OR 2.07; 95%CI, 1.47–2.93), without differences in sICH or mortality. Second Enhanced Control of Hypertension and Thrombectomy Stroke Study was limited by the early termination of the trial, which may have inflated the observed treatment effect and limits definitive conclusions.

The Blood Pressure After Endovascular Stroke Therapy-II (BEST-II) trial was a phase 2, multicentre, randomised, open-label, blinded-endpoint futility trial conducted at 3 US comprehensive stroke centres.^105^ It enrolled 120 AIS patients who achieved successful recanalisation following MT. Within 60 min of recanalisation, patients were randomised to 1 of 3 SBP targets to be maintained for 24 h: < 140 mmHg, < 160 mmHg or ≤ 180 mmHg (guideline-based control). The trial had 2 pre-specified primary outcomes for futility analysis: follow-up infarct volume at 36 ± 12 h and utility-weighted mRS (uw-mRS) score at 90 ± 14 days. No significant benefit was observed with lower BP targets. Mean infarct volumes were 32.4, 50.7 and 46.4 mL in the < 140, < 160 and ≤ 180 mmHg groups, respectively, while mean uw-mRS scores were 0.51, 0.47 and 0.58, respectively. The slope of infarct volume change per mmHg SBP decrease was –0.29 (95%CI –0.81 to ∞; futility *P* = .99), and the slope for uw-mRS score was –0.0019 (95%CI –∞ to 0.0017; futility *P* = .93). Predictive modeling showed a low probability of success for future phase 3 superiority trials, with predicted success rates of 25% and 14% for the < 140 mmHg and < 160 mmHg groups, respectively. While BEST-II did not formally meet futility criteria, the results suggest a low likelihood of clinical benefit from lower BP targets post-MT and support maintaining current guideline thresholds. Blood Pressure After Endovascular Stroke Therapy-II was limited by its small sample size, open-label design and futility-focused phase 2 framework, which precluded definitive conclusions about efficacy. Additionally, the use of infarct volume as a co-primary surrogate outcome and the enrollment of patients from only 3 centres may limit generalisability and clinical applicability.

The Blood Pressure Managementin Stroke Following Endovascular Treatment (DETECT) trial was a pilot RCT designed to assess the feasibility of post-thrombectomy BP management strategies in AIS patients.^106^ Thirty patients with persistent hypertension after successful MT were randomised within 1 h to either an intensive SBP target (<140 mmHg) or a standard target (<180 mmHg) for 48 h. Feasibility outcomes were met, with good protocol adherence in both arms (mean SBP: 131 ± 18 mmHg in the intensive group vs 139 ± 18 mmHg in the standard group). No significant differences were observed in exploratory endpoints, including neurological deterioration, functional outcomes, ICH or cerebral blood flow. However, this trial was limited by the small sample size and recruitment challenges mainly due to spontaneous BP normalisation after MT.

The Outcome in Patients Treated with Intraarterial Thrombectomy—OptiMAL Blood Pressure Control (OPTIMAL-BP) trial was a multicentre, randomised, open-label trial with blinded endpoint assessment conducted across 19 stroke centres in South Korea to evaluate the impact of post-MT BP targets in patients with successful reperfusion (mTICI ≥ 2b).^107^ A total of 306 patients were randomised to either intensive SBP management (<140 mmHg) or conventional management (140–180 mmHg) for 24 h. The trial was terminated early due to safety concerns. Good functional outcome at 90 days was significantly lower in the intensive group (39.4%) compared with the conventional group (54.4%) (adjusted OR 0.56; 95%CI, 0.33–0.96; *P* = .03). Rates of sICH and stroke-related mortality did not differ significantly between groups. Limitations of the OPTIMAL-BP trial include its early termination, which reduced statistical power and may have introduced bias in effect estimates.

**Figure 13:**
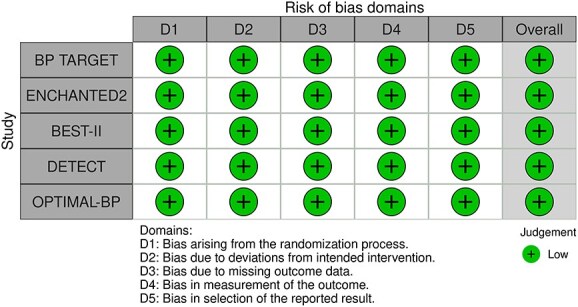
Risk of bias in each RCT of intensive blood lowering compared to conventional blood pressure lowering in patients with acute ischaemic stroke caused by LVO and following successful mechanical thrombectomy (with or without intravenous thrombolysis).

Module working group assessment of the risk of bias in each RCT according to the Cochrane RoB-2 tool is presented in Figure 13. All studies were considered to be at overall low risk of bias.

**Figure 14:**
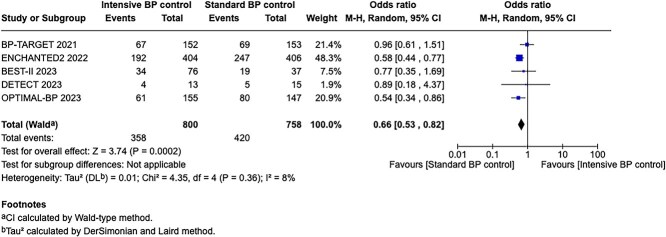
Good functional outcome (mRS 0–2 at 90 days) in patients receiving intensive BP control vs standard BP control (unadjusted pooled odds ratio, random-effects meta-analysis). Abbreviations: BP = blood pressure; M-H = Mantel–Haenszel.

**Figure 15:**
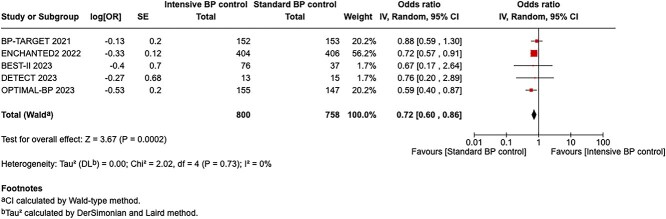
Pooled unadjusted common odds ratio for reduced disability (improvement of a least 1 point on the mRS at 90 days) in patients receiving intensive BP control vs standard BP control (unadjusted pooled common odds ratio, random-effects meta-analysis). Abbreviations: BP = blood pressure; IV = inverse variance.

**Figure 16:**
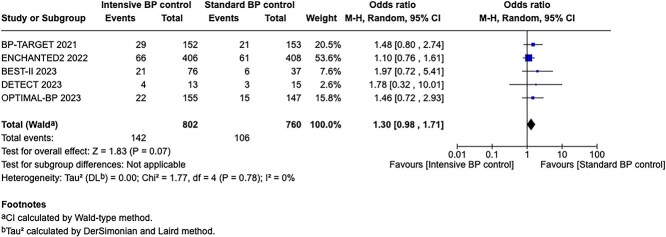
All-cause mortality at 3 months in patients receiving intensive BP control vs standard BP control (unadjusted pooled odds ratio, random-effects meta-analysis). Abbreviations: BP = blood pressure; M-H = Mantel–Haenszel.

**Figure 17:**
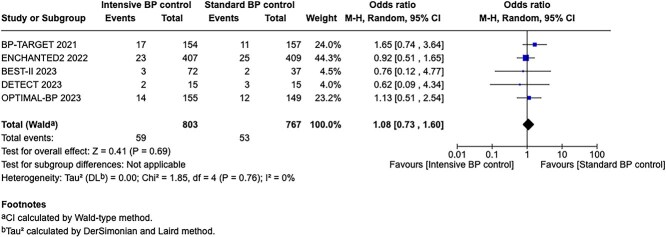
Symptomatic ICH in patients receiving intensive BP control vs standard BP control (unadjusted pooled odds ratio, random-effects meta-analysis). Abbreviations: BP = blood pressure; M-H = Mantel–Haenszel.

Compared to patients randomised to standard BP lowering, the pooled unadjusted OR for good functional outcome in patients randomised to intensive BP control was 0.66 (95%CI, 0.53–0.82; *P* < .001; Figure 14). The unadjusted common OR for reduced disability with intensive BP control was 0.72 (95%CI, 0.60–0.86; *P* < .001; Figure 15). All-cause mortality at 3 months was numerically higher in the intensive group but did not statistically differ between the 2 groups (OR 1.30; 95%CI, 0.98–1.71; *P* = .07; Figure 16). Finally, the odds of sICH did not differ between standard vs intensive BP control (OR 1.08; 95%CI, 0.73–1.60; *P* = .69; Figure 17). Table 6 provides details regarding the assessment of the quality of evidence for all outcomes evaluated in PICO 4.

### Additional information

Observational studies have consistently shown that extremely elevated BP values (particularly SBP and/or DBP levels exceeding 220/120 mmHg prior to MT as well as persistently elevated BP levels following MT) for LVO are associated with poorer clinical and radiological outcomes, including increased rates of sICH, mortality and functional dependence.^108–116^ As with IVT, markedly elevated BP is therefore commonly lowered in clinical practice prior to MT to remain within recommended thresholds, although there is currently no randomised evidence demonstrating that active BP reduction in this setting independently improves clinical outcomes.

In parallel, hypotension during MT is also a common occurrence, often linked to the choice of sedation, especially general anesthesia. Hypotension has been associated with infarct growth and worse functional outcomes at 3 months.^117^ Analysis of pooled individual patient data from 3 RCTs (SAGA collaborators) found that both low (<70 mmHg) and high (>90 mmHg) MAP during MT were associated with less favourable outcomes.^118^ Additionally, a ≥ 10% drop in MAP from baseline during MT has been correlated with poorer functional outcomes, regardless of sedation method.^119^ Further observational studies and meta-analyses have reinforced these findings, reporting that MAP reductions ≥ 10% during the procedure, sustained MAP < 70 mmHg for ≥ 10 min or MAP < 100 mmHg prior to recanalisation are all linked with adverse clinical outcomes.^120–122^ These data support the hypothesis that systemic BP drops may lead to hypoperfusion of the ischaemic penumbra, contributing to larger final infarct volumes.^121^^,^^122^

Furthermore, elevated SBP variability after MT appears to independently predict worse outcomes, irrespective of absolute BP levels.^92^^,^^123^ Recently, a pooled individual patient-data analysis of 2640 AIS patients across 5 studies found that increased BPV during the first 24 h after endovascular thrombectomy was independently associated with worse clinical outcomes.^124^ Specifically, patients in the highest tertile of SBP standard deviation or coefficient of variation had significantly higher odds of 90-day mortality, disability and functional impairment, even after adjusting for mean BP and other key confounders. These findings suggest that BPV—beyond absolute BP levels—may be a modifiable target to improve post-MT outcomes.^124^

Nonetheless, caution is warranted when interpreting these associations, as study heterogeneity remains substantial across patient characteristics (eg, comorbid hypertension or cardiac disease), recanalisation status (complete vs incomplete), BP measurement parameters (SBP/DBP vs MAP), reperfusion strategies (direct MT vs bridging therapy with IVT) and the classes of antihypertensive agents used (eg, β-blockers, CCBs or centrally acting drugs).

Two RCT protocols evaluating MT vs standard therapy in anterior LVO have also outlined recommendations for BP management. The Endovascular Treatment for Small Core and Proximal Occlusion Ischaemic Stroke (ESCAPE) trial protocol suggested maintaining SBP ≥ 150 mmHg prior to reperfusion to support collateral flow and advocated normalisation of BP post-recanalisation, with agents such as labetalol or low-dose IV beta-blockers like metoprolol for control.^125^ The Clinical Mismatch in the Triage of Wake Up and Late Presenting Strokes Undergoing Neurointervention With Trevo(DAWN) trial recommended maintaining SBP < 140 mmHg in the first 24 h after successful reperfusion.^126^ Additionally, 3 RCTs comparing anesthetic strategies for MT (Sedation vs Intubation for Endovascular Stroke TreAtment [SIESTA], Anesthesia During Stroke [ANSTROKE] and General Or Local Anesthesia in Intra Arterial Therapy [GOLIATH]) proposed intraprocedural SBP targets: 140–160 mmHg in SIESTA,^127^ 140–180 mmHg in ANSTROKE^128^ and > 140 mmHg in GOLIATH.^129^

Observational studies consistently show that patients with successful reperfusion (mTICI ≥ 2b) often experience spontaneous BP reduction post-MT, likely reflecting haemodynamic normalisation.^130^^,^^131^ Elevated post-reperfusion SBP has been inversely associated with good 3-month functional outcomes, with levels < 140 mmHg linked to improved prognosis.^132–134^ A single-centre study also found that spontaneous BP drop post-MT predicted dramatic neurological recovery (defined as 8-point-reduction in baseline NIHSS score or an overall NIHSS ≤ 2) at 24 h.^135^ In line with this observation, previous ESO guidelines advise that SBP drops during MT should be avoided^19^; however, no dedicated studies provide evidence on what degree of BP reduction is clinically relevant.

There is currently no randomised evidence supporting the use of induced hypertension in patients with successful recanalisation following MT. Concerns persist, especially in cases with complete reperfusion (mTICI 3), as transcranial Doppler studies suggest that elevated flow velocities post-MT may cause hyperperfusion injury in infarcted tissue.^136–138^ The MAnagement of Systolic blood pressure during Thrombectomy by Endovascular Route for acute ischaemic STROKE (MASTERSTROKE) trial is a randomised, multicentre trial, currently evaluating whether induced hypertension during MT—by targeting SBP at 170 ± 10 mmHg from anesthesia induction until recanalisation—can improve functional outcomes, based on the rationale that impaired autoregulation in ischaemic stroke may render cerebral perfusion pressure-dependent.^139^ The control group follows a standard target of 140 ± 10 mmHg, allowing the study to directly compare whether augmented peri-procedural pressures enhance collateral flow and neurological recovery.^139^

**Table 6:** GRADE evidence profile for PICO 4: In patients with acute ischaemic stroke caused by LVO and undergoing mechanical thrombectomy (with or without intravenous thrombolysis), does blood pressure lowering with any vasodepressor drug compared to no drug improve outcome?

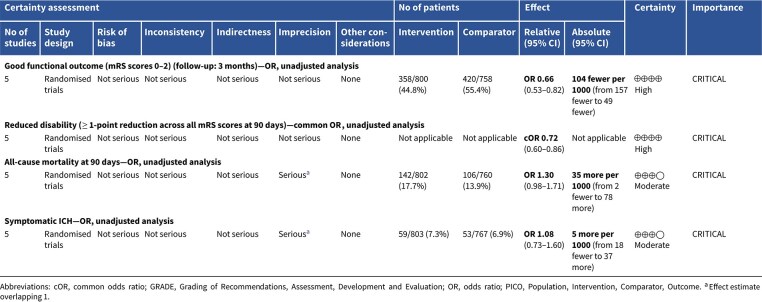

Finally, available randomised evidence to date does not support individualised BP management based on predefined patient-specific thresholds during MT.^103^^,^^104^ However, more dynamic, physiology-guided approaches that account for real-time cerebral haemodynamics may offer a more promising avenue for personalised care. A small observational study using near-infrared spectroscopy demonstrated that personalised autoregulatory thresholds could be identified in real-time, potentially guiding safer haemodynamic management post-MT.^140^ A recent prospective, non-randomised study found that transcranial Doppler-guided BP management after MT significantly improved 90-day functional outcomes and reduced mortality compared to standard guideline-based BP control, highlighting the promise of real-time cerebral haemodynamic monitoring for individualising post-MT care.^141^ The Haemodynamic Optimization of Cerebral Perfusion After Endovascular Therapy in Patients With Acute Ischaemic Stroke (HOPE) trial is an ongoing, phase 4, single-centre study in Spain, evaluating whether tailoring post-MT BP targets based on the degree of recanalisation improves outcomes compared to standard BP management. In the interventional arm, patients with partial reperfusion (TICI 2b) are maintained at SBP 140–160 mmHg, while those with near-complete or complete reperfusion (TICI 2c–3) are targeted to < 140 mmHg, aiming to balance perfusion support with the risk of hyperperfusion injury. The results of those ongoing RCTs will further inform clinical decisions in BP management among AIS patients receiving MT.

**Table:** 

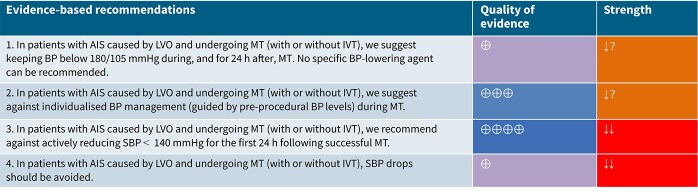

**Table:** 

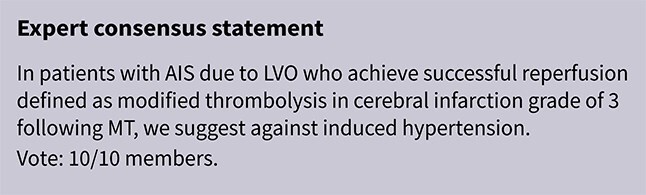

### PICO 5

In patients with AIS not treated with reperfusion therapies (IVT or MT) and with clinical deterioration, does induced hypertension by any vasopressor drug compared to no drug improve outcome?

### Analysis of current evidence

The hypertensive response observed in AIS is often considered a compensatory mechanism aimed at preserving cerebral perfusion in the setting of impaired cerebral circulation.^7^ Pharmacologically induced hypertension has been proposed to enhance cerebral perfusion, particularly in patients with LVO, fluctuating neurological deficits and low baseline BP who are either ineligible for or have not responded to acute reperfusion therapies.

The Safety and Efficacy of Therapeutic Induced HYPERTENSION (SETIN-HYPERTENSION) trial was a multicentre, prospective, randomised, open-label, blinded-endpoint trial, conducted in Korea.^142^ SETIN-HYPERTENSION evaluated induced hypertension by intravenous phenylephrine and enrolled 153 patients with significant neurological deficits (NIHSS 4–18), non-cardioembolic stroke etiology and either ineligibility for reperfusion or signs of stroke progression– defined as a ≥ 2-point NIHSS increase, including worsening limb motor function, and evidence of new or expanding infarction on diffusion-weighted imaging within 24 h. Patients with baseline SBP > 170 mmHg were excluded. The intervention group (*n* = 76) received intravenous phenylephrine to achieve a 20% increase in SBP from baseline. While induced hypertension was associated with greater neurological improvement at 7 days (OR 2.49; 95%CI, 1.25–4.96; *P* = .010), this did not lead to improved functional outcomes at 90 days (common OR 1.27; 95%CI, 0.72–2.22; *P* = .422). Mortality at 90 days was similar between groups (1.3% vs 0%; *P* = .313), and although the proportion of patients achieving good functional outcome at 90 days was higher in the intervention arm (75.0% vs 63.2%), the difference was not statistically significant (*P* = .114). Intracerebral haemorrhage (ICH) on follow-up MRI was more frequent in the induced hypertension group (6.6% vs 0%; *P* = .022), although sICH rates were comparable (1.3% vs 0%; *P* = .313). Several limitations temper the findings, including baseline imbalances between the intervention and the control group (younger age, more patients presenting with stroke progression included, higher baseline NIHSS scores, greater LVO prevalence in the intervention group), small sample size and limited generalisability given the single-country (Korea) study setting.

**Figure 18:**
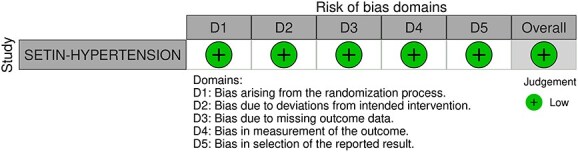
Risk of bias in the RCT of induced hypertension by any vasopressor drug compared to no drug in patients with acute ischaemic stroke not treated with reperfusion therapies (intravenous thrombolysis or mechanical thrombectomy) and with clinical deterioration.

Module working group assessment of the risk of bias in each RCT according to the Cochrane RoB-2 tool is presented in Figure 18. The included study was considered to be at overall low risk of bias.

**Figure 19:**
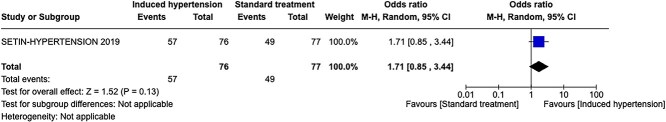
Good functional outcome (mRS 0–2 at 90 days) in patients receiving induced hypertension by any vasopressor drug compared to no drug (unadjusted pooled odds ratio, random-effects meta-analysis). Abbreviation: M-H = Mantel–Haenszel.

**Figure 20:**
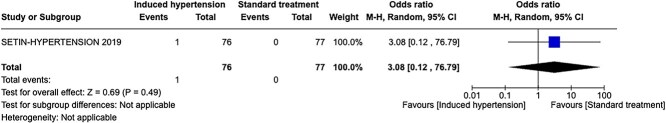
All-cause mortality at 3 months in patientsreceiving induced hypertension by any vasopressor drug compared to no drug (unadjusted pooled odds ratio, random-effects meta-analysis). Abbreviation: M-H = Mantel–Haenszel.

**Figure 21:**
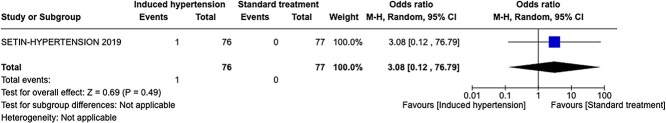
Symptomatic ICH in patients receiving induced hypertension by any vasopressor drug compared to no drug (unadjusted pooled odds ratio, random-effects meta-analysis). Abbreviation: M-H = Mantel–Haenszel.

Compared to patients randomised to standard BP management, the pooled unadjusted OR for good functional outcome in patients randomised to pharmacologically induced hypertension was 1.71 (95%CI, 0.85–3.44; *P* = .13; Figure 19). All-cause mortality at 3 months was similar between the 2 groups (OR 3.08; 95%CI, 0.12–76.79; *P* = .49; Figure 20). Finally, the odds of sICH did not differ between induced hypertension vs control (OR 3.08; 95%CI, 0.12–76.79; *P* = .49; Figure 21). Table 7 provides details regarding the assessment of the quality of evidence for all outcomes evaluated in PICO 5.

### Additional information

The Early Manipulation of Arterial Blood Pressure in Acute Ischaemic Stroke (MAPAS) trial was a randomised single-centre, prospective, open-label, blinded outcome assessment trial conducted in Brazil.^143^ The MAPAS trial randomised 218 AIS patients within 12 h of symptom onset to one of 3 SBP target groups maintained for 24 h: 140–160 mmHg (group 1), 161–180 mmHg (group 2) and 181–200 mmHg (group 3).^143^ Systolic BP was actively increased in 41% of participants overall, with norepinephrine administered to 17%, 48% and 62% of patients in groups 1, 2 and 3, respectively. No significant differences were seen in 90-day functional outcomes among groups. However, adverse events—including acute coronary syndrome and bradycardia—occurred in 4% of group 2 and 7.6% of group 3, both associated with norepinephrine use. Notably, Group 3 also had a significantly higher rate of sICH. However, the MAPAS trial did not specifically enroll patients with clinical deterioration. Therefore, it could not be analysed together with SETIN-HYPERTENSION, as it only partially aligns with the PICO question.

In a single-centre, small, pilot study, 15 patients within 7 days of AIS onset who exhibited a > 20% diffusion–perfusion mismatch on MRI and had measurable, stable or worsening deficits in aphasia, hemispatial neglect or hemiparesis were randomly assigned to receive phenylephrine-induced hypertension or no intervention.^144^ Greater improvements in NIHSS scores were observed in the induced hypertension group on both day 3 and day 90. However, functional outcome using the mRS at 90 days, all-cause mortality at 90 days or sICH were not assessed, thus the study was excluded from our meta-analysis.

Moreover, small observational studies suggest that phenylephrine-induced hypertension may be associated with neurological improvement in AIS patients with large artery atherosclerotic or small vessel disease.^145–149^

**Table 7:** GRADE evidence profile for PICO 5: In patients with acute ischaemic stroke not treated with reperfusion therapies (intravenous thrombolysis or mechanical thrombectomy) and with clinical deterioration, does induced hypertension by any vasopressor drug compared to no drug improve outcome?

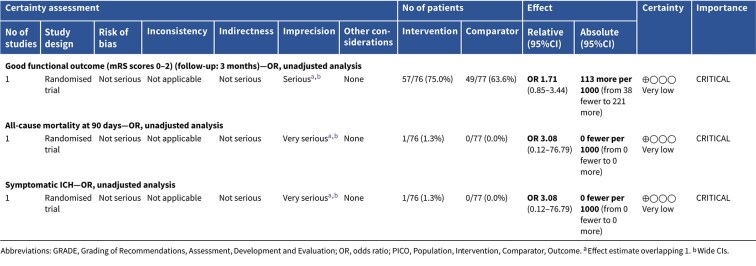

**Table:** 

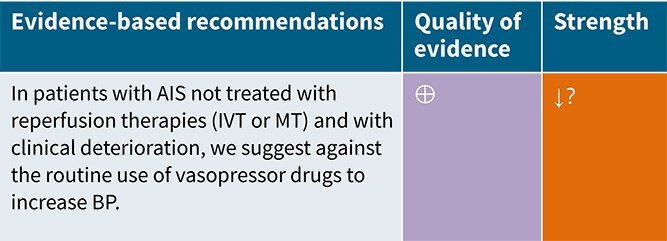

**Table:** 

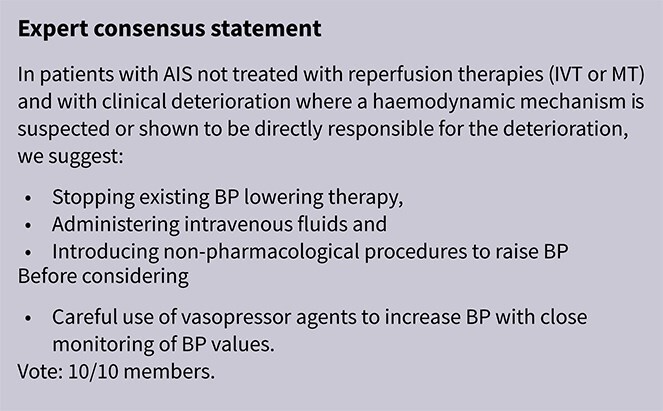

Norepinephrine was used as the primary vasopressor agent (when needed) in the INDIVIDUATE trial.^103^ This trial investigated whether targeting SBP to within ± 10 mmHg of the patient’s admission value during MT would improve outcomes compared to a standardised range of 140–180 mmHg. The trial showed no significant difference in good functional outcome at 90 days between the individualised and standard BP management arms. Importantly, the intervention was restricted to the intraprocedural period in patients undergoing MT for anterior circulation AIS, limiting its applicability to this PICO, which focuses on patients not receiving reperfusion therapy and experiencing clinical deterioration.

Norepinephrine-induced hypertension was also employed in the intervention arm of the DETERMINE trial, to maintain MAP within 10% of their initial pre-procedural MAP.^104^ According to the trial’s results, there was no difference in good functional outcome at 3 months, all-cause mortality at 3 months or sICH. However, as this trial specifically targeted the peri-procedural phase of MT in AIS patients, its relevance to the clinical context of this PICO is limited.

Finally, non-pharmacological measures, including head-of-bed positioning, volume optimisation, avoidance of early mobilisation and correction of reversible contributors to hypotension, may be considered in this setting with the goal of supporting cerebral perfusion rather than actively raising systemic BP. A recent phase 2 RCT showed that 0° head positioning for AIS patients with acute LVO and viable brain parenchyma was protective, maintaining clinical stability before MT. Moreover, the 0° head positioning was not associated with any excess risk (eg, aspiration pneumonia) compared to 30° head positioning.^150^

### PICO 6

In patients with AIS, does continuing vs temporarily stopping previous oral BP lowering therapy improve outcome?

### Analysis of current evidence

Blood pressure-lowering therapy is fundamental to the primary and secondary prevention of AIS. However, the optimal management of pre-existing antihypertensive medication during the acute phase of AIS remains uncertain. While continuation may help stabilise BP, it also raises concerns about reducing cerebral perfusion in already hypoperfused brain tissue.^2^

The Continue Or Stop post-Stroke Antihypertensives Collaborative Study (COSSACS) was a UK-based, multicentre, open-label, RCT with blinded outcome assessment that enrolled 763 stroke patients on prior antihypertensive therapy within 48 h of onset; 444 of them had AIS.^151^ In the predefined AIS subgroup, continuing antihypertensive therapy was associated with reduced death or dependency at 2 weeks (mRS ≥ 3) compared to stopping (19.1% vs 27.1%; relative risk reduction 0.70; 95%CI, 0.51–0.99; *P* = .045). A key limitation of the COSSACS trial was that it was terminated early due to slow recruitment and insufficient funding, resulting in a smaller-than-planned sample size, which may have limited the statistical power to detect modest treatment effects.

The ENOS trial was a large, international, multicentre, partial-factorial RCT, enrolling 4011 patients with AIS or ICH and raised SBP (140–220 mmHg) within 48 h of stroke onset to assess the effects of transdermal GTN and continuation vs temporary cessation of pre-stroke antihypertensive therapy.^40^ No significant differences regarding functional outcome at 90 days, all-cause mortality at 90 days, recurrent stroke at 7 days or sICH at 7 days were shown among the 2097 all-stroke patients included in the continue vs stop analysis. Among these, 1832 patients with AIS were randomised to either continue (*n* = 928) or stop (*n* = 904) their antihypertensive medication, with no significant difference observed in 90-day functional outcomes between the 2 groups. Interestingly, in a more recent, prespecified subgroup analysis of the ENOS trial, concerning the effect of continuing vs stopping pre-stroke antihypertensive agents within 12 h after stroke among 384 stroke patients (342 AIS patients), it was shown that continuing BP treatment shifted the mRS to a worse outcome by day 90 (adjusted common OR 1.46 [95%CI, 1.01–2.11] for all-stroke patients, and adjusted common OR 1.51 [95%CI, 1.04–2.20] for AIS patients in particular) and was associated with an increased mortality by day 90 (OR 2.77; 95%CI, 1.41–5.46; *P* = .003).^152^

An individual patient data meta-analysis combining COSSACS and ENOS data found no significant associations between continuing vs stopping previous BP lowering therapy and the odds of death or improved functional outcome at 3–6 months in the AIS subgroup (*n* = 2335).^153^

**Figure 22:**
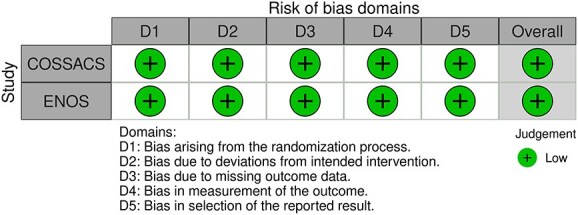
Risk of bias in the RCTs of continuing vs temporarily stopping previous oral blood pressure-lowering therapy in patients with acute ischaemic stroke.

Module working group assessment of the risk of bias in each RCT according to the Cochrane RoB-2 tool is presented in Figure 22. The included studies were considered to be at overall low risk of bias.

**Figure 23:**
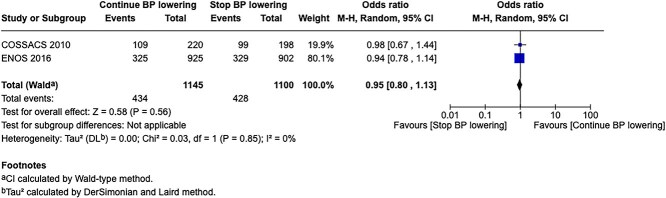
Good functional outcome (mRS 0–2 at 3–6 months) in patients continuing vs temporarily stopping previous oral blood pressure-lowering therapy (unadjusted pooled odds ratio, random-effects meta-analysis). Abbreviations: BP = blood pressure; M-H = Mantel–Haenszel.

**Figure 24:**
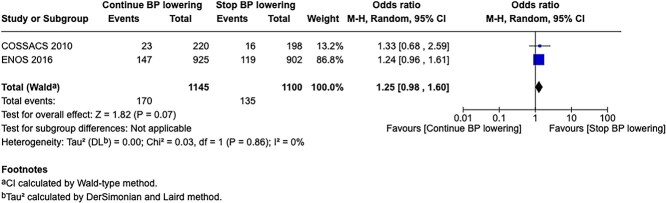
All-cause mortality at 3–6 months in patients continuing vs temporarily stopping previous oral blood pressure-lowering therapy (unadjusted pooled odds ratio, random-effects meta-analysis). Abbreviations: BP = blood pressure; M-H = Mantel–Haenszel.

Compared with patients randomised to temporarily stopping previous oral BP lowering therapy, the pooled unadjusted OR for good functional outcome at 3–6 months in patients randomised to continuing previous oral BP lowering therapy was 0.95 (95%CI, 0.80–1.13; *P* = .56; Figure 23). All-cause mortality at 3–6 months was numerically higher in the continuation group but did not statistically differ between the 2 groups (OR 1.25; 95%CI, 0.98–1.60; *P* = .07; Figure 24). Table 8 provides details regarding the assessment of the quality of evidence for all outcomes evaluated in PICO 6.

**Table 8:** GRADE evidence profile for PICO 6: In patients with acute ischaemic stroke, does continuing vs temporarily stopping previous oral blood pressure-lowering therapy improve outcome?

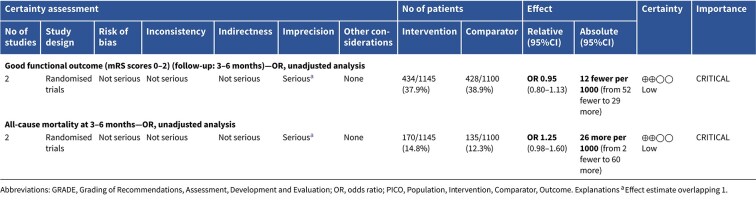

### Additional information

A recent multicentre, open-label RCT conducted in China (China antihypertensive trial in acute ischaemic stroke 2, CATIS-2) enrolled 4810 AIS patients within 24–48 h of symptom onset and elevated SBP (between 140 and < 220 mmHg).^154^ Patients treated with IVT and MT were excluded from this RCT. Patients were randomly assigned to receive antihypertensive treatment immediately after randomisation (aimed at reducing SBP by 10%–20% within the first 24 h and a mean BP < 140/90 mmHg within 7 days) or to discontinue antihypertensive medications for 7 days if they were taking any, and then receive treatment on day 8 (aimed at achieving mean BP < 140/90 mmHg). A total of 2413 patients were assigned to the early treatment group and 2397 were assigned to the delayed treatment group. Mean SBP was reduced by 9.7% (from 162.9 to 146.4 mmHg) in the early treatment group and by 4.9% (from 162.8 to 154.3 mmHg) in the delayed treatment group within 24 h after randomisation (*P* < .001). Mean SBP was 139.1 mmHg in the early treatment group and 150.9 mmHg in the delayed treatment group on day 7 (*P* < .001). At day 90, 289 trial participants (12.0%) in the early treatment group, compared with 250 (10.5%) in the delayed treatment group, had died or experienced dependency (mRS scores 3–6; OR 1.18; 95%CI, 0.98–1.41; *P* = .08). No significant differences in recurrent stroke or adverse events were reported between the 2 groups. In conclusion, early antihypertensive treatment compared to antihypertensive treatment discontinuation did not reduce the odds of dependency or death at 90 days among patients with mild-to-moderate AIS, SBP between 140 mmHg and less than 220 mmHg, and who did not receive acute reperfusion therapies (IVT and/or MT). This RCT was not included in our meta-analysis due to differences in study design (initiation of antihypertensive treatment immediately following randomisation in 1 treatment arm) in comparison to COSSACS^2^ and ENOS.^3^

In a recent, multicentre, observational study, more than half of the included AIS patients had one or more pre-stroke antihypertensive medications discontinued.^155^ In this study, the continuation, discontinuation or change of antihypertensive medication after stroke onset did not significantly affect 90-day mortality. However, this study was limited by its observational design, which introduces potential selection bias and limits causal inference. Additionally, the absence of standardised criteria for continuing, discontinuing or changing antihypertensive medications post-stroke may have influenced the outcomes and introduces variability in clinical decision-making.

In the absence of robust evidence favouring either strategy, decisions regarding continuation or temporary interruption of pre-stroke antihypertensive therapy should be individualised. Relevant considerations include reperfusion status, baseline and evolving BP levels, and whether antihypertensive treatment is required to maintain BP within recommended ranges. These factors are not intended to constitute prescriptive guidance but reflect pragmatic clinical considerations commonly encountered in routine care. Practical issues, such as dysphagia or reduced level of consciousness, may further influence management, as they affect the feasibility and route of antihypertensive administration. In these circumstances, oral antihypertensive therapy should be withheld until swallowing is safe or an alternative route is available.

**Table:** 

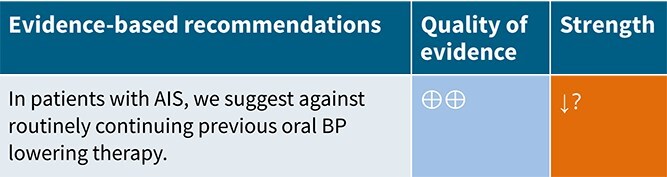

**Table:** 

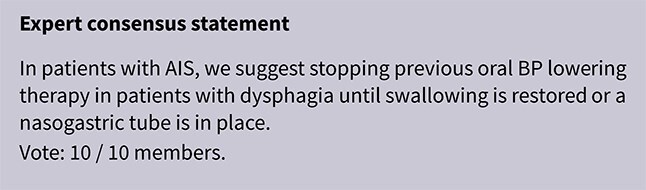

### PICO 7

In patients with acute ICH, does intensive BP lowering with any vasodepressor drug compared to control improve outcome?

## Analysis of current evidence

Elevated BP is common in patients with acute ICH and is associated with increased risk of haematoma expansion, death and dependence.^1^^,^^16^^,^^28^ The primary rationale for lowering BP in acute ICH is to prevent haematoma expansion, and thereby clinical deterioration.

Koch and colleagues conducted a single-centre RCT in the United States, including 42 patients with supratentorial ICH diagnosed within 8 h of symptom onset.^156^ Exclusion criteria were history of head trauma, ICH secondary to other causes, coma with signs of herniation, coagulopathy, MAP < 110 mmHg at presentation, surgical haematoma evacuation and inability to give informed consent. The study compared aggressive BP lowering (target MAP < 110 mmHg) with standard BP treatment (target MAP 110–130 mmHg) over 48 h. Agents used included intermittent labetalol infusions, continuous nicardipine infusion or sodium nitroprusside for severe cases. The primary outcome was neurological deterioration (defined as NIHSS ≥ 2-point increase) within 48 h. Secondary outcomes included 90-day functional outcomes (mRS) and haematoma expansion (≥30% increase in the initial ICH volume). The results showed no differences in neurological deterioration, haematoma expansion or 90-day clinical outcome between the 2 groups.

The INTensive blood pressure reduction in Acute Cerebral Haemorrhage trial (INTERACT) was a multicentre, randomised, pilot trial with blinded outcome assessment.^157^ It included 404 patients from China, Australia and South Korea who presented with ICH and SBP 150–220 mmHg within 6 h of symptom onset. The trial excluded patients with specific indications or contraindications for intensive BP lowering; ICH secondary to other causes, deep coma (GCS 3–5), ischaemic stroke within 30 days; notable pre-stroke disability or medical illness or planned early neurosurgery. INTensive blood pressure reduction in Acute Cerebral Haemorrhage trial compared 2 BP strategies: intensive reduction aiming for SBP < 140 mmHg within 1 h, and standard reduction targeting SBP < 180 mmHg within 6 h, using locally available medications (most frequently urapidil). The primary endpoint was change in proportional haematoma volume at 24 h. Secondary endpoints included clinical and safety outcomes at 90 days. Results indicated a significant reduction in haematoma growth in the intensive group (13.7%) compared with standard management (36.3%, difference = 22.6%, 95%CI, 0.6–44.5; *P* = .04), with no increase in adverse events.

The CHHIPS trial was a placebo-controlled, double-blind pilot trial conducted across 6 centres in the United Kingdom.^51^ A subgroup of 25 patients with ICH and SBP > 160 mmHg was randomised to labetalol or lisinopril, or placebo, within 36 h of symptom onset. The trial excluded patients with SBP > 200 mmHg and/or DBP > 120 mmHg; those on antihypertensive therapy without dysphagia, significant pre-stroke disability, impaired level of consciousness (NIHSS section 1a score ≥ 2), contraindications to trial therapy, premorbid dependence, life expectancy under 6 months, females of child-bearing potential or those with hypertensive encephalopathy and other cardiac or vascular emergencies. The primary outcome was death or dependency at 2 weeks post-stroke and occurred in 14/18 patients with active treatment and 3/7 with placebo. Secondary outcomes were early neurological deterioration, BP control, serious adverse events and 3-month mortality. Results showed no difference in mortality at 3 months.

**Figure 25:**
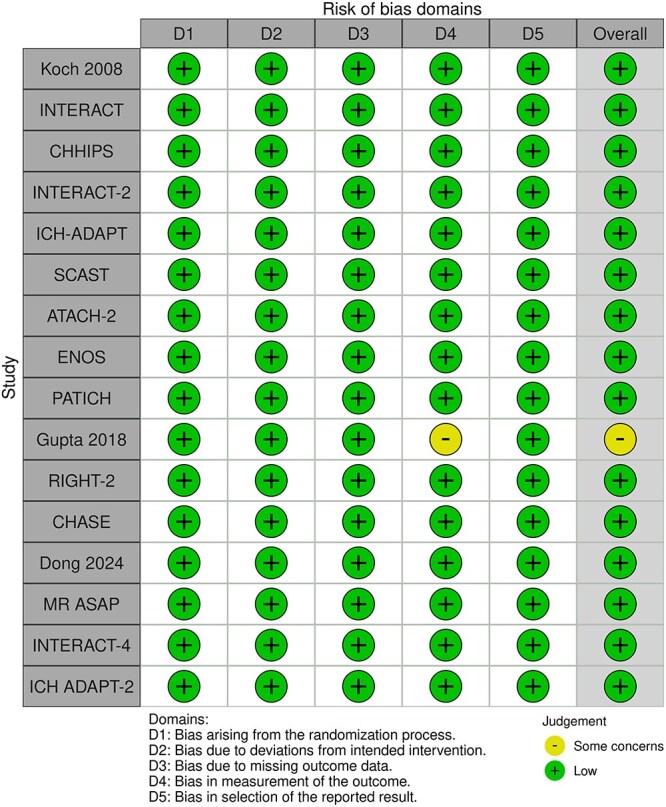
Risk of bias in each RCT of blood pressure-lowering therapies compared to control in patients with acute ICH.

**Figure 26:**
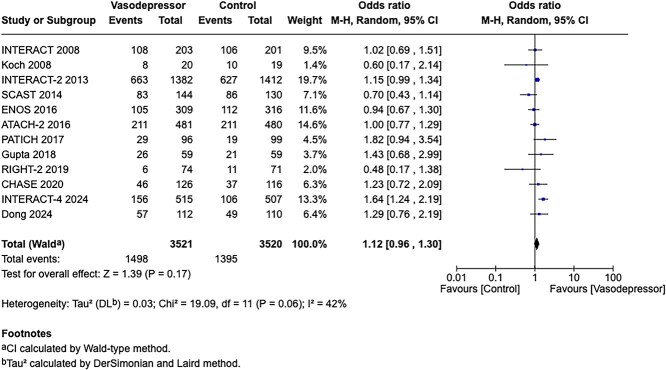
Effect on good functional outcome (mRS 0–2) at 3–6 months after intensive blood pressure lowering with any vasodepressor drug compared with control in adults with acute ICH.

**Figure 27:**
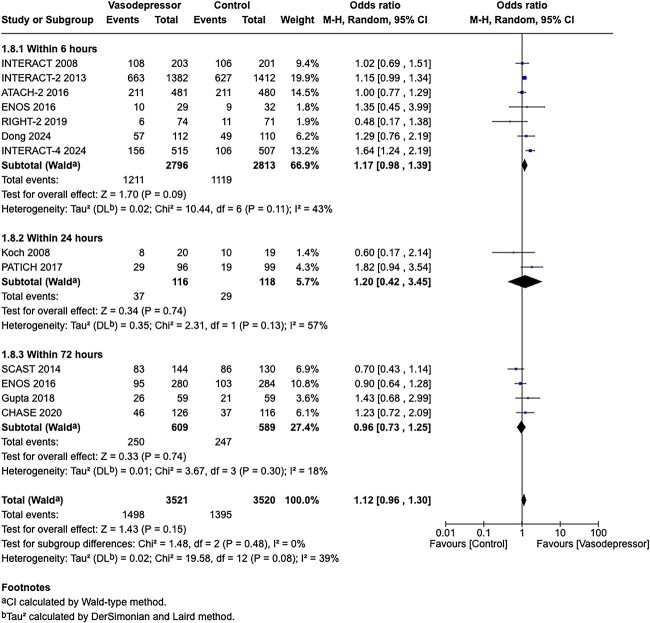
Effect on good functional outcome (mRS 0–2) at 3–6 months of intensive BP lowering with any vasodepressor drug compared with control following symptom onset in subgroups of adults with acute ICH stratified by time to treatment. This included trials enrolling patients within 6 h, those enrolling within 24 h (excluding trials enrolling patients within 6 h) and studies involving treatment within 72 h (excluding trials enrolling within 24 h). Abbreviation: BP = blood pressure.

**Figure 28:**
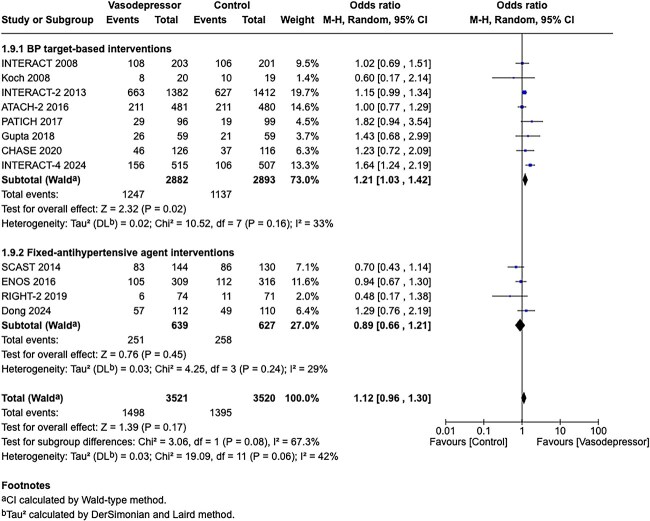
Effect on good functional outcome (mRS 0–2) at 3–6 months of intensive BP lowering with any vasodepressor drug compared with control in adults with acute ICH, stratified by intervention type (trials evaluating BP target–based interventions vs those assessing fixed antihypertensive agent interventions). Abbreviation: BP = blood pressure.

**Figure 29:**
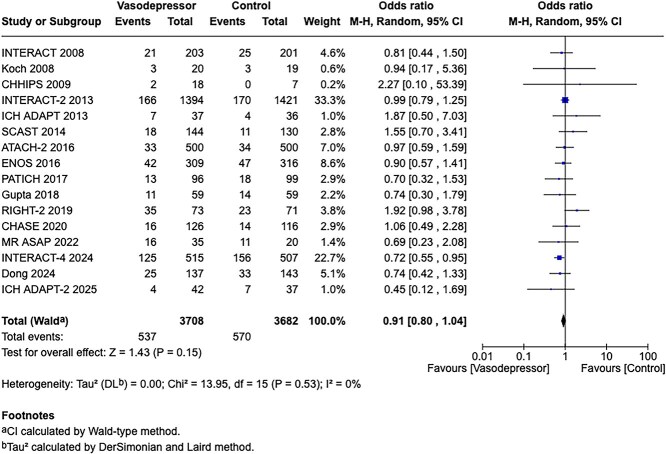
Effect on death within 3–6 months of intensive BP lowering with any vasodepressor drug compared with control in adults with acute ICH. Abbreviation: BP = blood pressure.

The second Intensive Blood Pressure Reduction in Acute Cerebral Haemorrhage Trial (INTERACT2) was a multicentre, randomised, blinded study involving 2839 participants from 144 hospitals in 21 countries.^29^ INTERACT2 enrolled patients with ICH and SBP 150–220 mmHg without a definite indication or contraindication to BP-lowering treatment within 6 h of symptom onset. Exclusions included structural cause for ICH, deep coma (GCS score 3–5), massive haematoma with poor prognosis and planned early surgery. Intensive BP lowering (target SBP < 140 mmHg within 1 h, maintained for 7 days or until discharge) was compared with standard treatment (SBP target < 180 mmHg). The primary outcome, death or dependency (mRS scores of 3–6) at 90 days, showed no difference between the groups. However, a secondary ordinal analysis of 3-month mRS showed better functional outcomes with intensive treatment (common OR for functional worsening 0.87; 95%CI, 0.77–1.00; *P* = .04). Rates of serious adverse events were comparable between groups.

The Intracerebral Haemorrhage Acutely Decreasing Arterial Pressure Trial (ICH-ADAPT) was a multicentre, randomised, open-label, blinded-endpoint trial in Canada involving 75 patients with ICH and SBP ≥ 150 mmHg within 24 h of onset.^158^ Exclusion included ICH secondary to other causes, urgent BP reduction needs, recent ischaemic stroke, planned haematoma surgery and contraindications to CT perfusion. Patients were assigned to SBP targets of < 150 or < 180 mmHg using intravenous labetalol, hydralazine and enalapril, aiming to reach targets within 1 h for at least 24 h. The primary outcome, perihaematomal cerebral blood flow measured by CT perfusion 2 h post-randomisation, showed no difference between groups. Secondary outcomes (BP difference at 1 and 2 h, 24-h haematoma expansion and 30- and 90-day mRS) likewise showed no difference, despite lower mean SBP at 2 h in the < 150 mmHg group.

In a subgroup analysis of the randomised, placebo-controlled, double-blinded ARB candesartan for treatment of acute stroke trial (SCAST), 274 patients with ICH and SBP ≥140 mmHg within 30 h of symptom onset were studied.^159^ Exclusion criteria included contraindications to or current treatment with an ARB, severe consciousness impairment (Scandinavian Stroke Scale consciousness score ≤ 2), a clear indication for antihypertensive treatment or ARB, pre-stroke disability (mRS ≥ 4), life expectancy under 12 months, unavailability for follow-up, pregnancy and breastfeeding. Patients received either candesartan or placebo in a fixed-dose escalation scheme for 7 days. Co-primary outcomes were a composite of vascular death, stroke or myocardial infarction, and 6-month functional outcome (mRS). Candesartan was associated with worse functional outcomes (adjusted common OR 1.61; 95%CI, 1.03–2.50).

The Antihypertensive Treatment of Acute Cerebral Haemorrhage-II (ATACH-2) trial was a multicentre, randomised study of 1000 participants from 6 countries with at least 1 SBP reading ≥ 180 mmHg, haematoma volume < 60 cm^3^ and a GCS ≥ 5.^160^ Participants were assigned to SBP targets of 110–139 mmHg (intensive) or 140–179 mmHg (standard), with intravenous nicardipine initiated within 4.5 h of symptom onset. Patients with intraventricular haemorrhage were excluded. The primary outcome, death or disability (mRS 4–6) at 3 months, showed no significant difference after enrolment was halted for futility at a pre-specified interim analysis. Serious adverse events within 72 h were similar, but the intensive group had more renal adverse events within 7 days (9.0% vs 4.0%, *P* = .002). A post hoc analysis suggested that initiating BP lowering within 2 h of symptom onset was associated with reduced haematoma expansion within 24 h (OR 0.56; 95%CI, 0.34–0.92; *P* = .02) and improved 3-month functional outcomes (OR 1.68; 95%CI, 1.01–2.83; *P* = .048).^161^

In the subgroup analysis of the multicentre, randomised, blinded ENOS trial, 629 participants from 23 countries across 5 continents with ICH and SBP 140–220 mmHg were randomised to 7 days of GTN or no GTN within 48 h from symptom onset.^162^ Previously independent, conscious patients (GCS > 8) with motor deficit were included. Exclusion criteria included contraindications to or definite need for nitrate or acute antihypertensive therapy, SBP < 140 mmHg or > 220 mmHg, planned surgical intervention, conditions precluding 3-month outcome assessment, women of childbearing potential, pregnancy and breastfeeding. The primary outcome at 90 days revealed no difference in functional outcomes or mortality. For those randomised within 6 h, GTN was associated with improved functional outcomes (adjusted common OR 0.19; 95%CI, 0.06–0.59; *P* = .004) and reduced mortality rates (7% vs 38%; *P* = .006) at 3 months, with no difference in serious adverse events.

The Perioperative Antihypertensive Treatment in Patients With Spontaneous Intracerebral Haemorrhage (PATICH) study was a singlecentre, randomised, assessor-blinded trial in China involving 201 patients with ICH, elevated SBP (150–220 mmHg), requiring surgery within 24 h after onset.^163^ Patients were randomised within 1 h of admission to either intensive treatment (aiming for SBP of 140–160 mmHg within the first hour after admission and 120–140 mmHg intra-operatively and post-operatively for 7 days) or conservative treatment (targeting 140–180 mmHg perioperatively and 90–140 mmHg during surgery). Exclusions included definite indications or contraindications to antihypertensives, secondary ICH, GCS 3–5, surgical contraindications, pre-existing advanced dementia or disability and comorbidities affecting outcome evaluation. The primary endpoint, re-haemorrhage within 7 days, defined by change in haematoma volume post-surgery, did not differ between groups, nor did 90-day mortality or serious adverse events.

Gupta and colleagues conducted an open-labelled, intention-to-treat, randomised trial at 3 centres in India, enrolling 118 patients with ICH within 72 h of symptom onset.^164^ The only exclusion criterion was a GCS score of 3. Patients were assigned to tight BP control, initiated within 1 h of admission if MAP ≥ 115 mmHg, or to conventional BP management if MAP ≥ 130 mmHg, with treatment continued for 72 h. The primary outcome was mRS at 90 days; secondary outcomes assessed safety and efficacy. Results showed no difference in the primary outcome (mRS 3–6 at 90 days), and intensive BP lowering was safe.

The RIGHT-2 trial, conducted across multiple UK centres using an ambulance-based, randomised, blinded design, included 145 patients with ICH.^32^ Inclusion required SBP ≥ 120 mmHg and a FAST score of 2 or 3 within 4 h of symptom onset. Exclusion criteria included residence in nursing homes, reduced consciousness (GCS < 8), hypoglycaemia or witnessed seizures. Patients were randomised to receive either transdermal GTN or a sham dressing for 4 days. The primary endpoint was mRS at 90 days. Secondary outcomes included death, serious adverse events, BP changes at hospital admission and other stroke-severity and recovery measures. Results indicated a possible decline in functional outcomes at 3 months with GTN (adjusted OR 1.87; 95%CI, 0.98–3.57) and a higher rate of in-hospital mortality (adjusted OR 2.26; 95%CI, 1.03–4.95), although there was no difference in 90-day mortality rates. As treatment with GTN was initiated in the ambulance before imaging, it remains uncertain whether these results are due to chance, confounding or a true drug effect.

In the Controlling Hypertension After Severe Cerebrovascular Event (CHASE) trial across 26 Chinese centres, 242 patients with ICH were studied.^57^ Participants presented within 72 h of symptom onset, had GCS ≤ 12 or NIHSS ≥ 11 and SBP 150–210 mmHg. Exclusions included ICH secondary to a structural cause, planned decompressive craniectomy, contraindications to antihypertensives, unstable vital signs, pre-existing dementia, pre-stroke disability or any condition affecting outcome evaluation. Patients were randomised to an individualised BP-lowering group, targeting a 10%–15% reduction in SBP to maintain SBP 130–180 mmHg for 1 week, or a standard group, maintaining SBP ≤ 180 mmHg. The primary outcome was poor functional outcome (dependence or death) at 90 days. Secondary outcome included poor outcome at discharge, neurological deficits (NIHSS), consciousness (GCS), activities of daily living (Barthel Index) and serious adverse events. Rates of poor functional outcome at 90 days were similar between groups, with comparable mortality and serious adverse events.

In the MR ASAP trial conducted at multiple centres in the Netherlands, an ambulance-based, open-label study with blinded endpoints, 56 patients with ICH were included.^35^ Inclusion required a probable diagnosis of stroke, FAST score 2 or 3 and SBP ≥ 140 mmHg within 3 h of symptom onset. Exclusions were considerable pre-stroke dependency in activities of daily living, reduced consciousness (GCS < 8), or known contraindication or hypersensitivity to GTN. Patients were randomised to transdermal GTN plus standard care or standard care alone. The primary endpoint was mRS at 90 days. Safety outcomes included death within 7 and 90 days and serious adverse events. Safety concerns, particularly an increased 7-day mortality in the GTN group, led to early trial cessation, although there was no significant impact on 90-day mortality.

The INTERACT4 trial was a multicentre, ambulance-delivered, randomised, open-label, blinded-endpoint trial involving more than 50 sites in China, enrolling hypertensive patients (SBP ≥ 150 mmHg) with suspected acute stroke and a FAST score of 2 or 3 with an arm motor deficit within 2 h of last-seen-well.^34^ A total of 1041 patients with ICH were randomised to intensive BP lowering (target SBP of 130–140 mmHg within 30 min for 7 days or until discharge) or standard BP management per local guidelines. Exclusions included coma (GCS < 5), severe comorbidities, history of epilepsy or recent head injury and hypoglycaemia. The agent used was intravenous urapidil. The primary outcome was 90-day functional recovery (mRS). Secondary outcomes included haematoma expansion over 24 h, 90-day mortality or dependency, neurologic deterioration and health-related quality of life. The pre-hospital BP-reduction group with ICH had a lower risk of a poor functional outcome (common OR 0.75; 95%CI, 0.60–0.92). Absolute haematoma expansion within 24 h was lower in the BP-reduction group than in the usual-care group (11.3% vs 18.2%; OR 0.59; 95%CI, 0.39–0.90). Rates of serious adverse events were similar between groups.

Dong and colleagues conducted a multicentre, parallel-group, single-blind, superiority RCT across 14 tertiary hospitals in China.^165^ They enrolled 338 patients with confirmed ICH and SBP ≥ 150 mmHg within 24 h of symptom onset who were candidates for emergency BP treatment and real-time monitoring. Exclusions included contradictions to BP lowering, recent ischaemic stroke, high immediate mortality risk (GCS 3–5, deep coma or midline shift), significant disability (mRS ≥ 3), dementia, coagulation disorders, opioid allergy, major interfering comorbidities (eg, malignancy), serious arrhythmias, pregnancy or investigator-determined ineligibility. Patients were randomised to a BP-lowering regimen using remifentanil (an opioid analgesic) and dexmedetomidine (an α2-agonist sedative, with sympatholytic effects) alongside guideline-based care, or to standard guideline-based BP management using conventional intravenous antihypertensives. The primary outcome was SBP control rate (<140 mmHg) at 1 h after treatment initiation. Secondary outcomes included BPV, haematoma growth, analgesic and sedative scores, neurologic function, length of ICU stay and mechanical ventilation, major disability (mRS 3–5) and mortality at 28 and 90 days and safety. The intervention group achieved a significantly higher 1-h SBP control rate and reduced BPV during procedures like suctioning and lighter levels of sedation. However, there were no significant differences between groups in haematoma growth, neurological outcomes, ICU/mechanical-ventilation duration or major disability or mortality at 28 and 90 days. Bradycardia and respiratory depression were more frequent in the intervention group, but events were reversible and no treatment-related deaths occurred.

**Figure 30:**
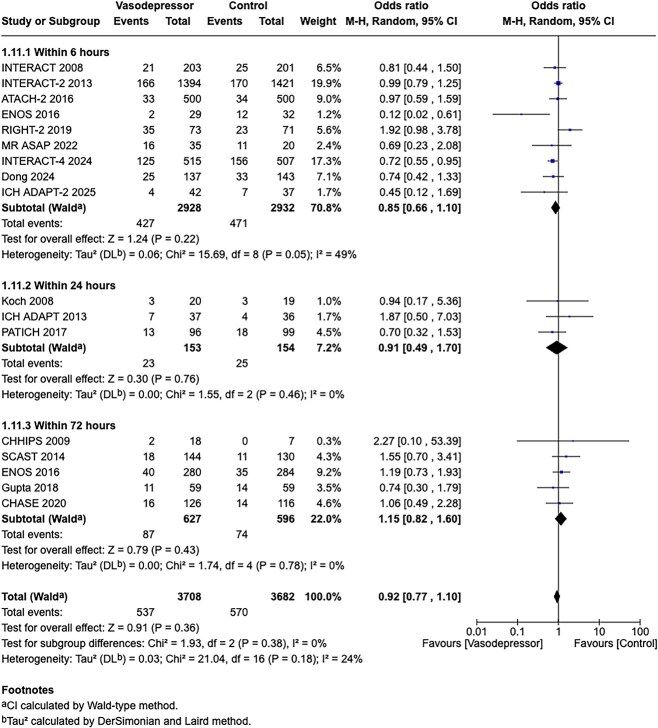
Effect on death within 3–6 months following symptom onset in subgroups of adults with spontaneous ICH stratified by time to treatment of intensive BP lowering with any vasodepressor drug compared with control. This included trials enrolling patients within 6 h, those enrolling within 24 h (excluding trials enrolling patients within 6 h) and studies involving treatment within 72 h (excluding trials enrolling within 24 h). Abbreviation: BP = blood pressure.

**Figure 31:**
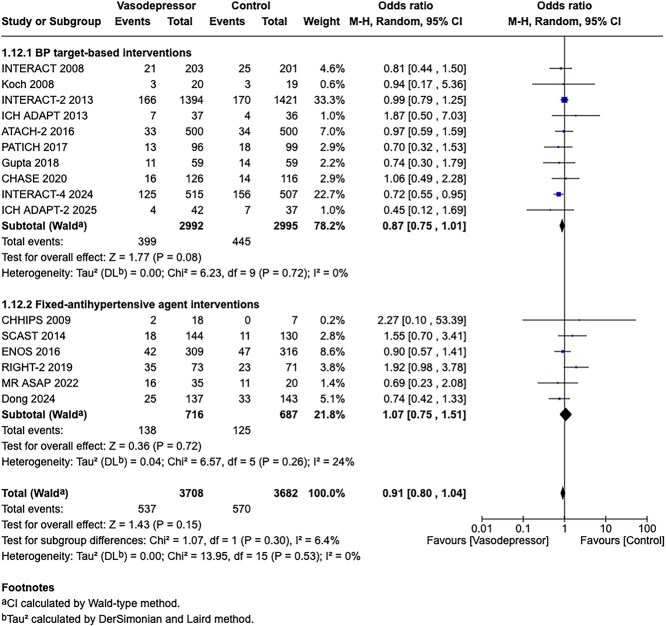
Effect on death within 3–6 months of intensive blood BP lowering with any vasodepressor drug compared with control in adults with acute ICH, stratified by intervention type (trials evaluating BP target–based interventions vs those assessing fixed-antihypertensive agent interventions). Abbreviation: BP = blood pressure.

**Figure 32:**
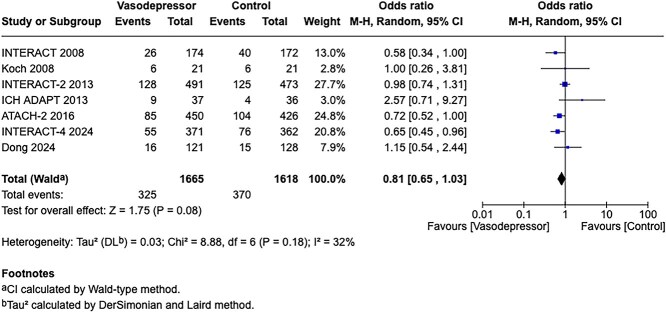
Effect on haematoma expansion of intensive BP lowering with any vasodepressor drug compared with control in adults with acute ICH. Abbreviation: BP = blood pressure.

**Figure 33:**
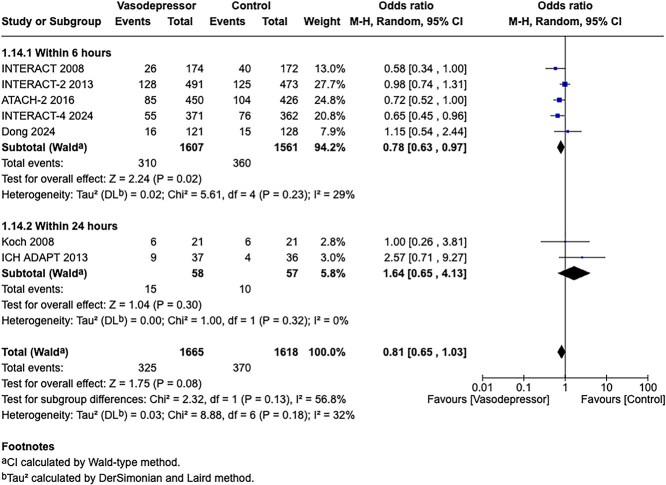
The effect on haematoma expansion in subgroups stratified by time to treatment of intensive BP lowering with any vasodepressor drug compared with control in adults with spontaneous ICH. This included studies enrolling patients within 6 h and those enrolling within 24 h (excluding trials enrolling within 6 h). Abbreviation: BP = blood pressure.

**Figure 34:**
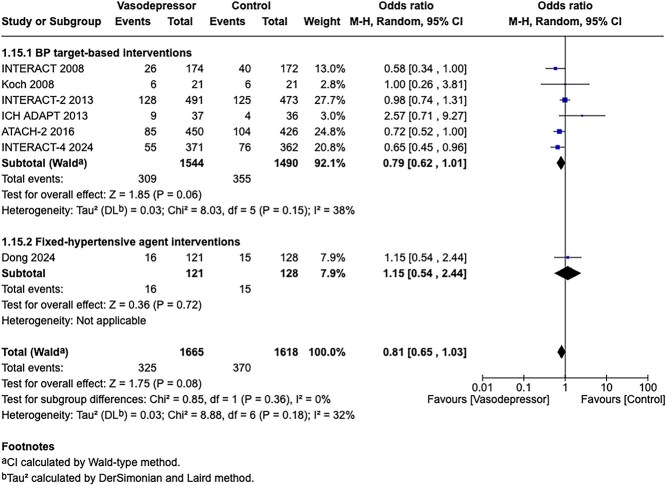
Effect on haematoma expansion of intensive BP lowering with any vasodepressor drug compared with control in adults with acute ICH, stratified by intervention type (trials evaluating BP target–based interventions vs those assessing fixed antihypertensive agent interventions). Abbreviation: BP = blood pressure.

The Intracerebral Haemorrhage Acutely Decreasing Arterial Pressure Trial 2 (ICH ADAPT-2) is a multicentre, prospective, randomised, open-label, blinded-endpoint trial across 3 stroke centres in Canada and Australia.^166^ Adults with ICH within 6 h of onset, SBP ≥ 140 mmHg and feasible for MRI were enrolled. Exclusions included large haematomas (>90 mL), very low GCS (≤5), secondary causes of haemorrhage, recent ischaemic stroke, premorbid disability (mRS ≥ 3), planned surgical evacuation and MRI contraindications. A total of 162 patients were randomised to acute SBP target of < 140 or < 180 mmHg, with BP managed using labetalol and hydralazine. The primary outcome was incidence of acute ischaemic lesions detected on diffusion-weighted MRI at 48 ± 12 h after randomisation. Secondary outcomes included lesion number and volume, DWI lesions at 7 and 30 days, haematoma expansion and 30-day mortality. Among 79 patients with MRI data, DWI-lesion incidence was similar between groups (31% vs 38%; *P* = .32), with no significant differences in lesion volume, number or clinical outcomes. Adverse-event rates were similar, and early intensive SBP reduction to < 140 mmHg did not increase the risk of ischaemic injury, supporting the safety of aggressive BP lowering in acute ICH.

The MWG’s assessment of risk of bias for each RCT, based on the Cochrane RoB 2 tool, is shown in Figure 25. Overall, the included studies were judged to have a low risk of bias. Table 9 presents a detailed assessment of the quality of evidence for all outcomes evaluated in PICO 7.

This meta-analysis includes 16 RCTs comparing BP lowering (either titrated to an intensive BP target or with a fixed antihypertensive drug) with control (contemporaneous guideline-standard BP target or placebo, respectively) in 7390 participants assessed within 2–72 h of symptom onset.^29^^,^^32^^,^^34^^,^^35^^,^^51^^,^^57^^,^^156–160^^,^^162–166^ Good functional outcome (defined as mRS scores of 0–2) and death at 3–6 months after ICH were prioritised as critical outcomes, with haematoma expansion at 6 and 24 h as an important outcome.

Blood pressure-lowering treatment targeting lower BP, compared with control, did not improve good functional outcome at 3–6 months (OR 1.12; 95%CI, 0.96–1.30; *P* = .17; Figure 26). Given that the CI includes both potential harm and benefit and the overall certainty of evidence is very low, this result should be interpreted with caution. The pooled estimate may be anchored by early, small trials with extreme effects and may not reflect findings from larger, more recent studies. Furthermore, the definition of “good functional outcome” (mRS 0–2) may not fully capture clinically meaningful differences across diverse patient populations.

The effect of BP lowering on good functional outcome did not differ across time from symptom onset to treatment/randomisation (6, 24 and 72 h; Figure 27).

Similarly, subgroup analysis stratified by intervention type (BP target-based vs fixed antihypertensive agent interventions) showed no significant difference in good functional outcome (mRS 0–2) at 3–6 months (Figure 28). However, within the subgroup of BP-targeted interventions, BP-lowering treatment was associated with higher odds of a good functional outcome at 3–6 months (OR 1.21; 95%CI, 1.03–1.42; *P* = .02; Figure 28).

Blood pressure-lowering treatment, compared with control, did not affect all-cause mortality at 3–6 months (OR 0.91; 95%CI, 0.80–1.04; *P* = .15; Figure 29). Notably, the low certainty of evidence and the narrow range of effect estimates again raise concerns about insufficient statistical power to detect a modest but clinically relevant benefit.

The effect of BP lowering on death did not differ according to time from symptom onset to treatment/randomisation (6, 24 and 72 h, Figure 30).

Similarly, subgroup analysis stratified by intervention type (BP target-based vs fixed antihypertensive agent interventions) showed no significant difference regarding death within 3–6 months (Figure 31).

Antihypertensive treatment targeting lower BP, regardless of time to treatment, compared with control showed a non-significant reduction in haematoma expansion (OR 0.81; 95%CI, 0.65–1.03; *P* = .08; Figure 32).

However, BP lowering within 6 h of symptom onset was associated with lower odds of haematoma expansion (OR 0.78; 95%CI, 0.63–0.97; *P* = .02; Figure 33).

Similarly, subgroup analysis stratified by intervention type (BP target-based vs fixed antihypertensive agent interventions) showed no significant difference regarding haematoma expansion (Figure 34).

This meta-analysis suggests that early BP lowering, within 6 h of symptom onset, limits haematoma expansion in patients with minor to moderate ICH. However, owing to substantial clinical and methodological heterogeneity across the included studies—such as differences in intervention protocols, patient characteristics, timing and outcome definitions—interpretation of the pooled effect sizes should be approached with caution. Notably, standard random-effects models may inadequately account for heterogeneity, and early small studies can anchor pooled estimates, reducing the influence of larger, later trials. Although there was a tendency towards better outcomes with lower BP targets, no statistically significant effect on functional outcomes or death was observed at 3 months. The quality of evidence is moderate, as detailed in the evidence profile table (Table 9). The evidence mainly applies to conscious patients with SBP < 220 mmHg. The effects on patients with large haematomas, those requiring surgical decompression, severely elevated BP (>220 mmHg) or severe premorbid disabilities remain uncertain.

**Table 9:** In patients with acute ICH, does intensive blood pressure lowering with any vasodepressor drug compared to control improve outcome?

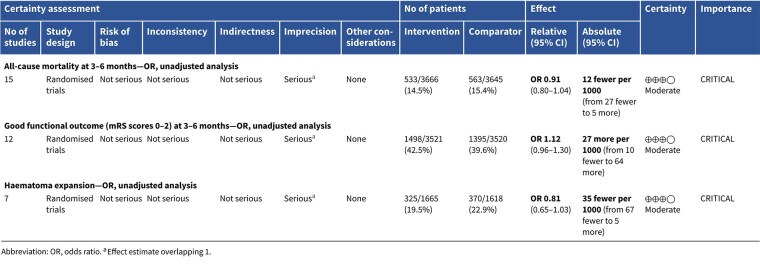

## Additional information

Haematoma expansion occurs predominantly within the first 3 h of stroke onset.^167^ Among the RCTs analysed, only the pre-hospital trials (RIGHT-2, MR ASAP and INTERACT4) recruited patients within an average of 3 h, with INTERACT4 enrolling all patients within 2 h.^32^^,^^34^^,^^35^ However, BP lowering in undifferentiated stroke should be avoided because of the potential for harm in ischaemic stroke.^32^^,^^35^

Additional insights come from the INTERACT IPDM, which pooled 11,312 acute ICH patients across the 4 INTERACT trials.^168^ In this analysis, intensive BP lowering (target SBP < 140 mmHg within 1 h) significantly decreased the odds of poor functional outcome (mRS 3–6) at follow-up (OR 0.85; 95%CI, 0.78–0.91) and was also associated with reduced odds of neurological deterioration, death and any serious adverse events. In the CT substudy (*n* = 2921), intensive treatment did not significantly reduce absolute or relative haematoma growth overall. However, treatment effects on both functional outcome and relative haematoma growth demonstrated strong time-dependence. The benefit of intensive BP lowering diminished progressively with later treatment initiation, with the effect crossing unity at approximately 3 h from symptom onset. These findings indicate that the therapeutic window for maximal benefit is narrow and centred within the first 3 h. Importantly, the benefits of early treatment were consistent across baseline severity strata, and safety outcomes did not differ by haematoma volume or ICH score. The INTERACT IPDM, therefore, strengthens the evidence supporting rapid initiation of BP lowering in acute ICH and highlights time-to-treatment as a key modifier of therapeutic effect.

High SBP variability during the acute phase of ICH is associated with poor outcomes.^169–172^ In addition to initiating treatment as soon as possible after symptom onset, post hoc exploratory analyses and observational studies suggest that sustained reduction in SBP (<140 mmHg) is safe and associated with better functional outcomes.^173–175^ A pooled analysis of INTERACT2 and ATACH-2 demonstrated that BP reductions exceeding 70 mmHg within the first hour were associated with unfavourable functional outcomes.^176^ In a prospective cohort of 448 patients with acute spontaneous ICH, the extent of BP reduction was examined in relation to acute kidney injury among those with and without chronic kidney disease.^177^ Reductions exceeding 90 mmHg from baseline SBP were associated with an increased risk of acute kidney injury.

A post hoc analysis of the ATACH-2 trial suggested that, in patients with mild-to-moderate ICH, targeting an SBP reduction of 55–85 mmHg within the first 2 h may optimise the balance between therapeutic benefit and adverse events.^178^ Conversely, in adults presenting with an initial SBP of ≥ 220 mmHg, another ATACH-2 post hoc analysis found that intensive BP lowering was associated with increased neurological deterioration within 24 h, without reducing haematoma expansion at 24 h or death or severe disability at 90 days.^179^ The safety of intensive BP reduction in adults with moderate to large haematomas remains uncertain due to limited data. However, 1 ATACH-2 analysis suggests a reduced frequency of haematoma expansion in this group, though without a corresponding effect on death or disability at 90 days.^180^ Notably, the majority of adults enrolled in the major trials had mild-to-moderate haematoma volumes (<30 mL)^29^^,^^34^^,^^157^^,^^160^ and the safety and efficacy of intensive BP lowering in those with larger haematomas (>30 mL) remains inadequately established.

**Table:** 

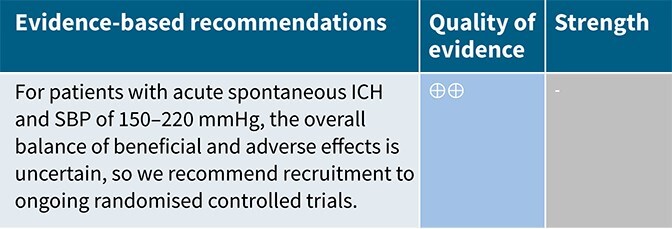

**Table:** 

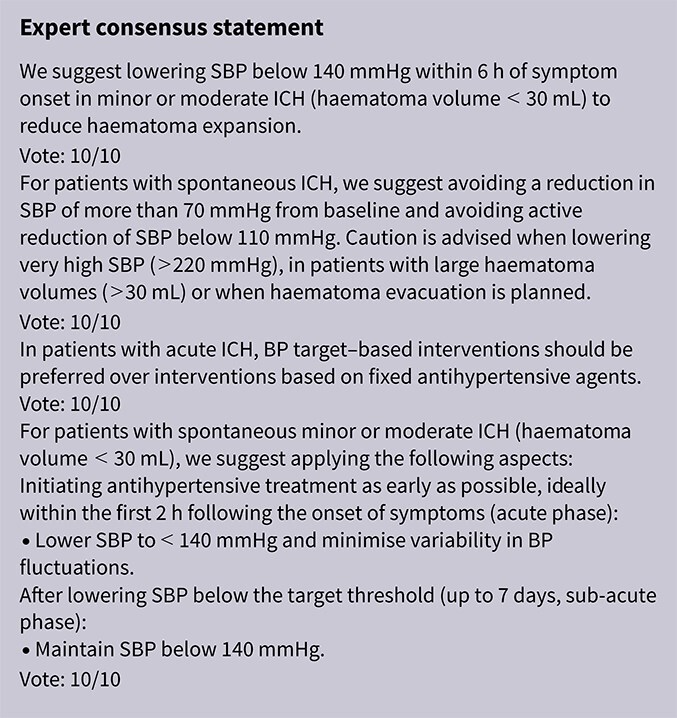

The optimal antihypertensive agent and duration of intensive BP lowering also remain uncertain. To enable rapid yet controlled BP reduction without excessive lowering, agents should be fast-acting with short half-lives. Various antihypertensive drugs meeting these criteria to varying degrees have been tested in the RCTs. These include labetalol, nicardipine, lisinopril, enalapril, candesartan, urapidil, hydralazine, GTN and sodium nitroprusside, with the choice often dictated by local availability. Apart from venous vasodilators (such as nitrates), no major safety concerns have been reported.^36^ The CCB clevidipine, with a 1.5-min half-life, may offer particularly effective BP control.^181^ Comparative evidence from a systematic review of intravenous antihypertensives in acute neurovascular emergencies suggests that clevidipine may achieve target BP more rapidly than nicardipine and may have less BPV compared with labetalol, although overall certainty of evidence remains very low.^182^ Regarding the optimal duration of BP lowering, maintaining achieved BP targets during the sub-acute phase may be clinically important to limit BPV, avoid rebound hypertension and reduce the risk of early recurrent stroke, all of which have been associated with worse outcomes in acute ICH,^92^ even after the period of highest risk for haematoma expansion.

Blood pressure control is a key element of early bundled care in ICH and is associated with reduced disability and death. In the INTERACT3 trial, intensive BP lowering was the most frequently applied and the most influential element of a bundle that also included anticoagulation reversal, glucose control and fever management.^183^ This bundle was associated with a reduced risk of poor functional outcome (mRS 3–6) (common OR 0.86; 95%CI, 0.76–0.97; *P* = .015).^184^ Similarly, the ABC-ICH study found that rapid BP management, together with anticoagulation reversal and timely neurosurgery referral for selected patients, significantly improved treatment times and 30-day mortality, with over half of the mortality benefit attributable to fewer early do-not-resuscitate orders.^185^

Our meta-analysis included 2 additional trials (Dong et al. and ICH ADAPT-2)^165^^,^^166^ compared with the meta-analysis informing the recently published ESO—European Association of Neurosurgical Societies (EANS) guideline on the management of stroke due to ICH.^186^ The 2 meta-analyses yielded identical results and the evidence-based recommendations as well as the expert consensus statements of the 2 guidelines are consistent.

Although early BP lowering below 140 mmHg is supported in selected patients with minor or moderate ICH, important uncertainties remain regarding optimal BP targets across the broader ICH population, supporting the continued inclusion of patients with SBP 150–220 mmHg in ongoing and future clinical trials. Current ongoing clinical trials are: TIME-ICH (NCT06760078), CLUTCH (NCT06402968) and Efficacy and Safety Study of Urapidil Alone or With Esmolol in Treating Acute Hypertensive Intracerebral Haemorrhage (NCT06635707), MAX-ICH Pilot Trial (NCT06648369) and I-CATCHER (NCT06429332).

**Figure 35:**
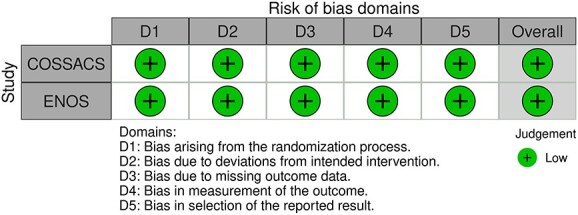
Risk of bias in the RCTs of continuing vs temporarily stopping previous oral blood pressure-lowering therapy in patients with acute ICH.

### PICO 8

In patients with acute ICH, does continuing vs temporarily stopping previous oral antihypertensive therapy improve outcome?

### Analysis of current evidence

Blodd pressure-lowering therapy is a key strategy for the primary and secondary prevention of ICH and other major cardiovascular events. However, it remains unclear if it is better to continue or temporarily stop prior ongoing antihypertensive treatment in the setting of an acute ICH. One theoretical argument in favour of continuing BP-lowering therapy in acute ICH is that more effective BP control may, in turn, limit haematoma expansion. Conversely, continuing oral antihypertensive agents could worsen functional outcome by compromising cerebral blood flow and perihaematomal perfusion; furthermore, it may increase the risk of aspiration pneumonia.

The RCT, ENOS, enrolled 246 patients with acute ICH and elevated SBP (140–220 mmHg) who were receiving antihypertensive therapy before admission. Participants were randomised to continue or stop their BP-lowering treatment for 7 days.^187^ At 90 days, there was no difference in mRS scores between groups (common OR for worse functional outcome, defined by one-point increase across all mRS categories, in the continuation group: 0.92; 95%CI, 0.45–1.89; *P* = .83).^187^ Mortality at 90 days did not differ significantly (16.0% vs 18.3% in the continue and stop groups, respectively), and data on haematoma expansion were not reported.

A meta-analysis of individual patient data from the COSSACS and ENOS trials evaluated the effect of continuing vs temporarily stopping prior BP-lowering therapy on death or dependency in 2860 patients with acute stroke.^153^ In the ICH subgroup, the analysis found no significant association between continuing and temporarily stopping previous BP-lowering therapy with respect to the odds of death or improved functional outcome.^153^

Two trials were included in the meta-analysis comparing continuation with stopping prior BP-lowering therapy.^40^^,^^151^ The MWG’s assessment of risk of bias for each RCT, based on the Cochrane RoB 2 tool, is presented in Figure 35. Overall, the included studies were judged to have a low risk of bias. Table 10 provides the detailed assessment of the quality of evidence for all outcomes evaluated in PICO 8.

**Figure 36:**
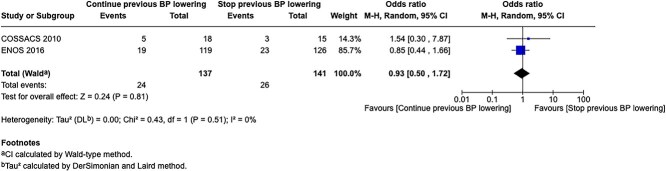
The effect of continuing vs temporarily stopping previous blood pressure-lowering therapy on mortality at 3–6 months following symptom onset in patients with acute ICH.

**Figure 37:**
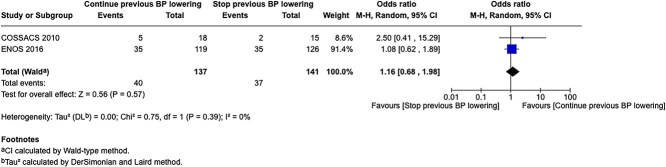
The effect of continuing vs temporarily stopping previous blood pressure-lowering therapy on good functional outcome (defined as mRS scores 0–2) at 3–6 months following symptom onset in patients with acute ICH.

No significant differences were observed between continuing and stopping prior BP-lowering therapy in 3–6 month mortality (OR 0.93; 95%CI, 0.50–1.72; *P* = .81; Figure 36) or in the proportion of patients achieving a good functional outcome (mRS 0–2) at 3–6 months (OR 1.16; 95%CI, 0.68–1.98; *P* = .57; Figure 37).

### Additional information

When deciding whether to continue or to temporarily stop previous antihypertensive agents in patients with ICH, clinicians should consider the individual patient’s BP levels and whether antihypertensive therapy is required to maintan these within the range recommended for aute ICH. In clinical practice, the most common scenario is the need for BP-lowering therapy to achieve target levels, in which case continuation of previous antihypertensive agents may support more stable BP control. However, continuation of oral antihypertensive agents may be challenging in patients with dysphagia or impaired consciousness, where the route of administration depends on swallowing ability and/or level of consciousness.

**Table 10:** In patients with acute ICH, does continuing vs temporarily stopping previous oral antihypertensive therapy improve outcome?

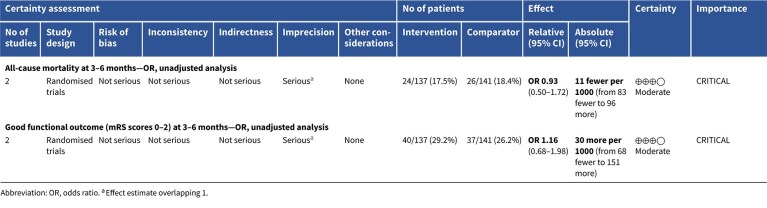

**Table 11:** Synoptic table of all recommendations and expert consensus statements.

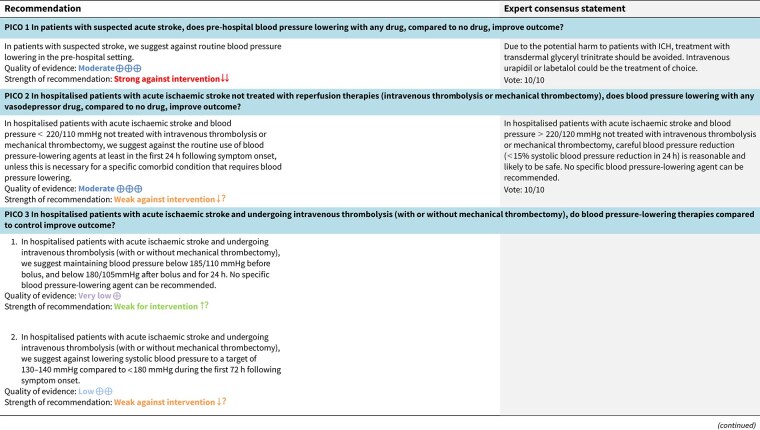

**Table 11:** 

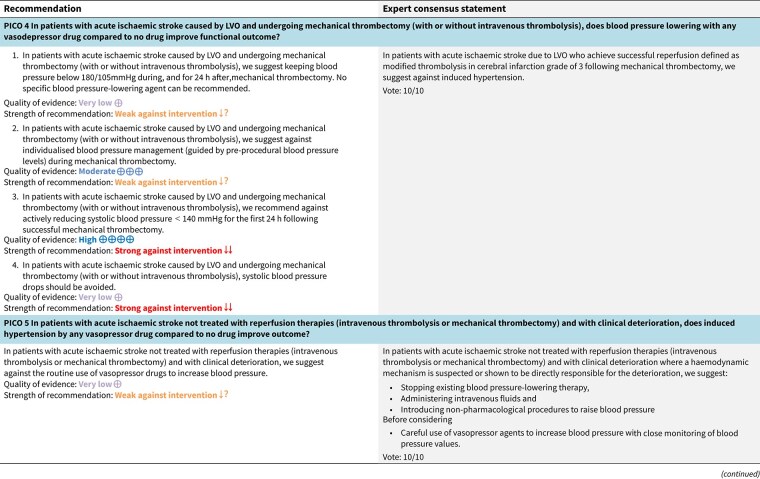

**Table 11:** 

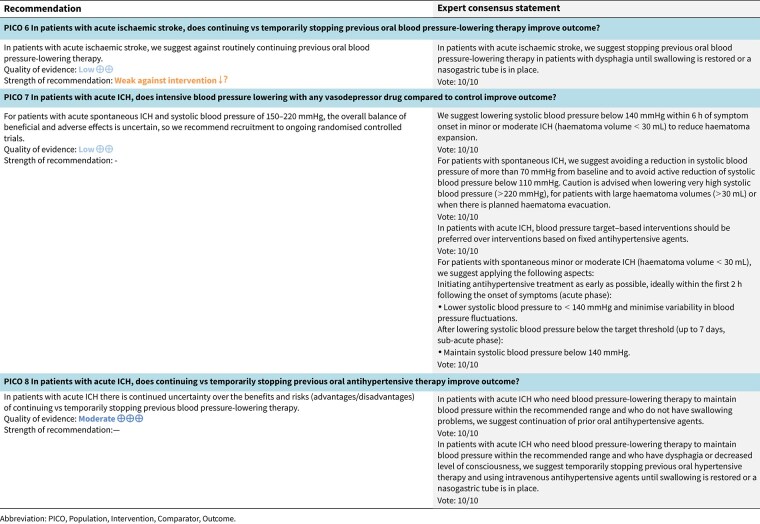

**Table:** 

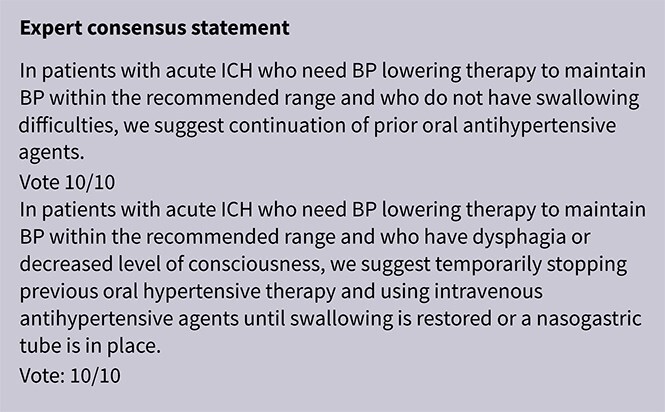

## Discussion

These updated ESO guidelines, developed in accordance with the ESO standard operating procedures and GRADE methodology, address 8 key clinical questions on acute BP management in AIS and ICH. They integrate the latest trial evidence with expert consensus. Overall, the updated recommendations reaffirm the core principles of current practice while refining guidance for specific clinical scenarios. All recommendations and expert consensus statements are summarised in Table 11.

**Table:** 

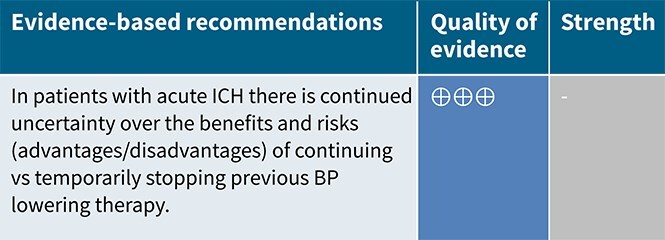

We continue to provide separate recommendations for AIS and ICH, with specific guidance for AIS patients undergoing acute reperfusion therapies (IVT and/or MT). Although many of the trials informing these guidelines enrolled patients with both subtypes, this distinction reflects important differences in pathophysiology and treatment considerations. In AIS, overly rapid or intensive BP reduction may compromise perfusion of the ischaemic penumbra and worsen outcomes, whereas, in ICH, uncontrolled hypertension drives haematoma expansion, making timely BP control critical. Despite new trials, our updated meta-analyses have not demonstrated that BP lowering alters the harmful association between high BP and short- or long-term outcomes observed in observational studies of either AIS or ICH, suggesting that this relationship is likely multifactorial and that BP manipulation alone may be insufficient to improve functional outcome. Notably, this updated guideline recommends against actively reducing SBP < 140 mmHg within the first 24 h after successful MT, based on high-certainty evidence from RCTs published after the 2021 ESO guidelines on acute BP management.

A key strength of this guideline is its systematic literature search and application of the GRADE methodology, providing clinicians with robust, evidence-based guidance. However, the certainty of evidence remains moderate to low or very low, reflecting several limitations, including a scarcity of large, high-quality RCTs and heterogeneity in treatment protocols regarding timing, targets, drug choice and patient selection. Although most trials were judged to have a low risk of bias using the ROB2 tool, several important limitations fall outside the scope of ROB2, including small sample sizes, restricted generalisability and substantial variability in outcomes assessed across studies. These issues contribute to wide CIs and imprecision, and they help explain why many trial results are inconsistent or neutral despite acceptable internal validity assessments. Together, these factors limit the strength and generalisability of the recommendations and highlight the complexity of BP management in acute stroke, where epidemiological findings, pathophysiological insights and randomised trial data often diverge. In recognition of these gaps, the writing group issued 11 expert consensus statements to provide pragmatic guidance where robust evidence is lacking. These statements were formally voted on by the working group, achieving excellent agreement.

Several unanswered questions remain. Future trials in acute stroke BP management should move beyond “one-size-fits-all” approaches to define optimal BP targets, timing and treatments for specific AIS and ICH subgroups, considering premorbid hypertension, baseline BP and relative rather than arbitrary absolute BP reductions. In AIS, trial design should account for stroke subtype, recanalisation status, collateral flow, estimated risk of reperfusion injury and differentiate BP strategies before, during and after reperfusion, exploring personalised, autoregulation-guided thresholds. In ICH, research should focus on ultra-early BP lowering, optimal treatment intensity and duration, effects in patients with large haematomas or imaging signs of ongoing bleeding and ideal antihypertensive agents to reduce BPV. Across both stroke types, comparative studies of antihypertensive agents and integration into comprehensive acute stroke care bundles are needed to guide precise, patient-specific BP management. Future studies should also incorporate neuroimaging markers of brain frailty (eg, white matter hyperintensities, cerebral microbleeds) and test the interactions between these parameters and effects of treatment.

## Supplementary Material

supplementary_tables_aakag004

BP_Management_Search_LH_111125_for_supplement_aakag004
