# Supplementary material for: 2025 update to European Stroke Organisation (ESO) guideline on blood pressure management in acute ischaemic stroke and intracerebral haemorrhage
Source: Eur Stroke J. 2026 May 7;11(5):aakag004. doi: 10.1093/esj/aakag004 (PMC13151662; doi:10.1093/esj/aakag004)
Supplement: supplementary_tables_aakag004 [file supplementary_tables_aakag004.docx]

**Supplementary Tables**

**Table S1:** Complete list of outcomes and their corresponding Delphi ratings for each PICO question.

**Table S2:** Summary of intervention and control treatments in the randomized-controlled clinical trials included in PICO 1–8.

**Table S1:** Complete list of outcomes and their corresponding Delphi ratings for each PICO question.

| **Outcome** | **Mean score** |
| --- | --- |
| **PICO 1:** In patients with suspected acute stroke, does pre-hospital blood pressure lowering with any drug, compared to no drug, improve outcome? | |
| Acute Ischaemic Stroke Subgroup | |
| Functional outcome (90 days or the end of follow-up) | 8.8/9 |
| Mortality (90 days or end of follow-up) | 8.2/9 |
| Symptomatic ICH | 7.5/9 |
| Early neurological deterioration | 7.2/9 |
| Hypotensive episodes requiring intervention | 6.7/9 |
| Quality of life (90 days) | 6.4/9 |
| Acute kidney injury | 6.2/9 |
| Recurrent ischaemic stroke | 5.9/9 |
| Intracerebral Haemorrhage Subgroup | |
| Functional outcome (90 days or the end of follow-up) | 8.8/9 |
| Mortality (90 days or end of follow-up) | 8.5/9 |
| Haematoma expansion | 7.8/9 |
| Recurrent ICH | 6.7/9 |
| Early neurological deterioration | 6.7/9 |
| Hypotensive episodes requiring intervention | 6.3/9 |
| Quality of life (90 days) | 6.1/9 |
| Acute kidney injury | 6.1/9 |
| Incident ischaemic stroke | 6.0/9 |
| **PICO 2:** In hospitalised patients with acute ischaemic stroke not treated with reperfusion therapies (intravenous thrombolysis or mechanical thrombectomy), does blood pressure lowering with any vasodepressor drug, compared to no drug, improve outcome? | |
| Functional outcome (90 days or the end of follow-up) | 8.8/9 |
| Mortality (90 days or end of follow-up) | 8.6/9 |
| Symptomatic ICH | 7.3/9 |
| Early neurological deterioration | 7.0/9 |
| Quality of life (90 days) | 6.6/9 |
| Recurrent ischaemic stroke | 6.4/9 |
| Acute kidney injury | 6.2/9 |
| Hypotensive episodes requiring intervention | 6.1/9 |
| **PICO 3:** In hospitalised patients with acute ischaemic stroke and undergoing intravenous thrombolysis (with or without mechanical thrombectomy), do blood pressure lowering therapies compared to control improve outcome? | |
| Functional outcome (90 days or the end of follow-up) | 8.8/9 |
| Mortality (90 days or end of follow-up) | 8.6/9 |
| Symptomatic ICH | 7.7/9 |
| Early neurological deterioration | 6.9/9 |
| Quality of life (90 days) | 6.4/9 |
| Recurrent ischaemic stroke | 6.4/9 |
| Hypotensive episodes requiring intervention | 6.3/9 |
| Acute kidney injury | 6.0/9 |
| **PICO 4:** In patients with acute ischaemic stroke caused by large vessel occlusion and undergoing mechanical thrombectomy (with or without intravenous thrombolysis), does blood pressure lowering with any vasodepressor drug compared to no drug improve functional outcome? | |
| Functional outcome (90 days or the end of follow-up) | 8.8/9 |
| Mortality (90 days or end of follow-up) | 8.6/9 |
| Symptomatic ICH | 7.5/9 |
| Early neurological deterioration | 6.9/9 |
| Recurrent ischaemic stroke | 6.6/9 |
| Quality of life (90 days) | 6.5/9 |
| Hypotensive episodes requiring intervention | 6.3/9 |
| Acute kidney injury | 6.0/9 |
| **PICO 5:** In patients with acute ischaemic stroke not treated with reperfusion therapies (intravenous thrombolysis or mechanical thrombectomy) and with clinical deterioration, does induced hypertension by any vasopressor drug compared to no drug improve outcome? | |
| Functional outcome (90 days or the end of follow-up) | 8.8/9 |
| Mortality (90 days or end of follow-up) | 8.6/9 |
| Symptomatic ICH | 7.8/9 |
| Early neurological deterioration | 6.8/9 |
| Recurrent ischaemic stroke | 6.7/9 |
| Quality of life (90 days) | 6.3/9 |
| Hypotensive episodes requiring intervention | 6.0/9 |
| Acute kidney injury | 5.9/9 |
| **PICO 6:** In patients with acute ischaemic stroke, does continuing versus temporarily stopping previous oral blood pressure lowering therapy improve outcome? | |
| Functional outcome (90 days or the end of follow-up) | 8.6/9 |
| Mortality (90 days or end of follow-up) | 7.9/9 |
| Recurrent ischaemic stroke | 7.2/9 |
| Symptomatic ICH | 7.0/9 |
| Early neurological deterioration | 6.5/9 |
| Quality of life (90 days) | 6.4/9 |
| Hypotensive episodes requiring intervention | 5.9/9 |
| Acute kidney injury | 5.3/9 |
| **PICO 7:** In patients with acute intracerebral haemorrhage, does intensive blood pressure lowering with any vasodepressor drug compared to control improve outcome? | |
| Functional outcome (90 days or the end of follow-up) | 8.8/9 |
| Mortality (90 days or end of follow-up) | 8.6/9 |
| Haematoma expansion | 7.8/9 |
| Early neurological deterioration | 6.9/9 |
| Quality of life (90 days) | 6.5/9 |
| Recurrent ICH | 6.4/9 |
| Hypotensive episodes requiring intervention | 6.4/9 |
| Acute kidney injury | 6.3/9 |
| Incident ischaemic stroke | 6.0/9 |
| **PICO 8:** In patients with acute intracerebral haemorrhage, does continuing versus temporarily stopping previous oral antihypertensive therapy improve outcome? | |
| Functional outcome (90 days or the end of follow-up) | 8.5/9 |
| Mortality (90 days or end of follow-up) | 8.1/9 |
| Haematoma expansion | 7.4/9 |
| Recurrent ICH | 6.7/9 |
| Early neurological deterioration | 6.6/9 |
| Hypotensive episodes requiring intervention | 6.4/9 |
| Incident ischaemic stroke | 6.3/9 |
| Quality of life (90 days) | 6.3/9 |
| Acute kidney injury | 5.6/9 |

**Table S2:** Summary of intervention and control treatments in the randomized-controlled clinical trials included in PICO 1–8.

| **Study** | **Intervention** | **Control** |
| --- | --- | --- |
| **PICO 1:** In patients with suspected acute stroke, does pre-hospital blood pressure lowering with any drug, compared to no drug, improve outcome? | | |
| RIGHT^1^ | GTN | No GTN |
| RIGHT-2^2^ | GTN | Sham dressing |
| MR ASAP^3^ | GTN | Standard care |
| INTERACT4^4^ | Target SBP: 130-140 mmHg using urapidil | Standard BP management |
| **PICO 2:** In hospitalised patients with acute ischaemic stroke not treated with reperfusion therapies (intravenous thrombolysis or mechanical thrombectomy), does blood pressure lowering with any vasodepressor drug, compared to no drug, improve outcome? | | |
| BEST^5^ | Atenolol or propranolol | Placebo |
| Gelmers 1998^6^ | Nimodipine | Placebo |
| ANSG 1992^7^ | Nimodipine | Placebo |
| INWEST^8^ | Nimodipine | Placebo |
| Kaste 1994^9^ | Nimodipine | Placebo |
| Bath 2001^10^ | GTN | Placebo |
| ACCESS^11^ | Candesartan | Placebo |
| Rashid 2003^12^ | GTN | Placebo |
| Fogelholm 2004^13^ | Nimodipine | Placebo |
| Eveson 2007^14^ | Lisinopril | Placebo |
| CHHIPS^15^ | Labetalol or lisinopril | Placebo |
| PRoFESS^16^ | Telmisartan | Placebo |
| CATIS^17^ | 10-25% SBP reduction within 24 hours using angiotensin inhibitors (first line) | Standard BP management |
| ENOS^18^ | GTN | Placebo |
| SCAST^19^ | Candesartan | Placebo |
| VENTURE^20^ | Valsartan | No treatment |
| Li 2018^21^ | Amlodipine or irbesartan | No antihypertensive drug |
| RIGHT-2^2^ | GTN | Sham dressing |
| CHASE^22^ | 10-15% SBP reduction within 2 hours (no specific agent) | Standard BP management |
| **PICO 3:** In hospitalised patients with acute ischaemic stroke and undergoing intravenous thrombolysis (with or without mechanical thrombectomy), do blood pressure lowering therapies compared to control improve outcome? | | |
| ENCHANTED^23^ | target SBP 130-140 mm Hg within 1 hour (no specific agent) | Standard BP management |
| ENOS^18^ | GTN | Placebo |
| Bath 2016^24^ | GTN | Placebo |
| ENCHANTED2-MT^25^ | target SBP 110-129 mm Hg within 3 hours (no specific agent) | target SBP 130-149 mm Hg within 3 hours (no specific agent) |
| BP-TARGET^26^ | target SBP 100-129 mm Hg within 1 hour (no specific agent) | target SBP 130-185 mm Hg within 1 hour (no specific agent) |
| **PICO 4:** In patients with acute ischaemic stroke caused by large vessel occlusion and undergoing mechanical thrombectomy (with or without intravenous thrombolysis), does blood pressure lowering with any vasodepressor drug compared to no drug improve functional outcome? | | |
| BP-TARGET^26^ | target SBP 100-129 mm Hg within 1 hour (no specific agent) | target SBP 130-185 mm Hg within 1 hour (no specific agent) |
| ENCHANTED2-MT^25^ | target SBP 110-129 mm Hg within 3 hours (no specific agent) | target SBP 130-149 mm Hg within 3 hours (no specific agent) |
| BEST-II^27^ | target SBP <160 mm Hg for 24 hours (no specific agent) | target SBP≤180 mm Hg for 24 hours (no specific agent) |
| DETECT^28^ | target SBP <140 mm Hg within 1 hour (no specific agent) | target SBP <180 mm Hg within 1 hour (no specific agent) |
| OPTIMAL-BP^29^ | target SBP <140 mm Hg for 24 hours (no specific agent) | target SBP 140-180 mm Hg for 24 hours (no specific agent) |
| **PICO 5:** In patients with acute ischaemic stroke not treated with reperfusion therapies (intravenous thrombolysis or mechanical thrombectomy) and with clinical deterioration, does induced hypertension by any vasopressor drug compared to no drug improve outcome? | | |
| SETIN-HYPERTENSION^30^ | Phenylephrine to achieve 20% increase in SBP from baseline | Best medical treatment |
| **PICO 6:** In patients with acute ischaemic stroke, does continuing versus temporarily stopping previous oral blood pressure lowering therapy improve outcome? | | |
| COSSACS^31^ | Continue pre-existing antihypertensive drugs (no specific agent) | Stop pre-existing antihypertensive drugs |
| ENOS^18^ | Continue pre-existing antihypertensive drugs (no specific agent) | Stop pre-existing antihypertensive drugs |
| **PICO 7:** In patients with acute intracerebral haemorrhage, does intensive blood pressure lowering with any vasodepressor drug compared to control improve outcome? | | |
| Koch 2008^32^ | target MAP < 110 mm Hg (no specific agent) | target MAP 110–130 mm Hg (no specific agent) |
| INTERACT^33^ | Target SBP <140 mmHg within 1 hour (most frequently using urapidil) | Target SBP <180 mmHg within 1 hour (most frequently using urapidil) |
| CHHIPS^15^ | Labetalol or lisinopril | Placebo |
| INTERACT2^34^ | Target SBP <140 mmHg within 1 hour (no specific agent) | Target SBP <180 mmHg within 1 hour (no specific agent) |
| ICH-ADAPT^35^ | Target SBP <150 mmHg within 1 hour (using labetalol, hydralazine, enalapril) | Target SBP <180 mmHg within 1 hour (using labetalol, hydralazine, enalapril) |
| SCAST^19^ | Candesartan | Placebo |
| ATACH-2^36^ | Target SBP 110-139 mmHg within 4.5 hours (no specific agent) | Target SBP 140-179 mmHg within 4.5 hours (no specific agent) |
| ENOS^37^ | GTN | No GTN |
| PATICH^38^ | Target SBP 140-160 mmHg within 1 hour (no specific agent) | Target SBP 140-180 mmHg within 1 hour (no specific agent) |
| Gupta 2018^39^ | Antihypertensive initiation when MAP ≥ 115 mmHg (no specific agent) | Antihypertensive initiation when MAP ≥ 130 mmHg (no specific agent) |
| RIGHT-2^2^ | GTN | Sham dressing |
| CHASE^22^ | 10-15% SBP reduction within 2 hours (no specific agent) | Standard BP management |
| MR ASAP^3^ | GTN | Standard care |
| INTERACT4^4^ | Target SBP: 130-140 mmHg using urapidil | Standard BP management |
| Dong 2024^40^ | Remifentanil and dexmedetomidine | Standard BP management |
| ICH ADAPT-2^41^ | Target SBP <140 mmHg using labetalol and hydralazine | Target SBP <180 mmHg using labetalol and hydralazine |
| **PICO 8:** In patients with acute intracerebral haemorrhage, does continuing versus temporarily stopping previous oral antihypertensive therapy improve outcome? | | |
| ENOS^42^ | Continue pre-existing antihypertensive drugs (no specific agent) | Stop pre-existing antihypertensive drugs |
| COSSACS^31^ | Continue pre-existing antihypertensive drugs (no specific agent) | Stop pre-existing antihypertensive drugs |

GTN: Glyceryl Trinitrate; SBP: systolic blood pressure; BP: blood pressure; MAP: mean arterial pressure

**References**

1. Ankolekar S, Fuller M, Cross I, et al. Feasibility of an ambulance-based stroke trial, and safety of glyceryl trinitrate in ultra-acute stroke: the rapid intervention with glyceryl trinitrate in Hypertensive Stroke Trial (RIGHT, ISRCTN66434824). *Stroke* 2013; 44: 3120-3128.

2. Prehospital transdermal glyceryl trinitrate in patients with ultra-acute presumed stroke (RIGHT-2): an ambulance-based, randomised, sham-controlled, blinded, phase 3 trial. *Lancet* 2019; 393: 1009-1020.

3. van den Berg SA, Uniken Venema SM, Reinink H, et al. Prehospital transdermal glyceryl trinitrate in patients with presumed acute stroke (MR ASAP): an ambulance-based, multicentre, randomised, open-label, blinded endpoint, phase 3 trial. *Lancet Neurol* 2022; 21: 971-981.

4. Li G, Lin Y, Yang J, et al. Intensive Ambulance-Delivered Blood-Pressure Reduction in Hyperacute Stroke. *N Engl J Med* 2024; 390: 1862-1872.

5. Barer D, Cruickshank J, Ebrahim S and Mitchell J. Low dose beta blockade in acute stroke (“BEST” trial). *British Medical Journal - BMJ* 1988; 296: 737-741.

6. Gelmers HJ, Gorter K, de Weerdt CJ and Wiezer HJ. A controlled trial of nimodipine in acute ischemic stroke. *N Engl J Med* 1988; 318: 203-207.

7. Clinical trial of nimodipine in acute ischemic stroke. The American Nimodipine Study Group. *Stroke* 1992; 23: 3-8.

8. Wahlgren NG, MacMahon DG, De Keyser J, et al. Intravenous Nimodipine West European Stroke Trial (INWEST) of Nimodipine in the Treatment of Acute Ischaemic Stroke. *Cerebrovascular Diseases* 1994; 4: 204-210.

9. Kaste M, Fogelholm R, Erilä T, et al. A randomized, double-blind, placebo-controlled trial of nimodipine in acute ischemic hemispheric stroke. *Stroke* 1994; 25: 1348-1353.

10. Bath PM, Pathansali R, Iddenden R and Bath FJ. The effect of transdermal glyceryl trinitrate, a nitric oxide donor, on blood pressure and platelet function in acute stroke. *Cerebrovasc Dis* 2001; 11: 265-272.

11. Schrader J, Lüders S, Kulschewski A, et al. The ACCESS Study: evaluation of Acute Candesartan Cilexetil Therapy in Stroke Survivors. *Stroke* 2003; 34: 1699-1703.

12. Rashid P, Weaver C, Leonardi-Bee J, et al. The effects of transdermal glyceryl trinitrate, a nitric oxide donor, on blood pressure, cerebral and cardiac hemodynamics, and plasma nitric oxide levels in acute stroke. *J Stroke Cerebrovasc Dis* 2003; 12: 143-151.

13. Fogelholm R, Palomäki H, Erilä T, et al. Blood pressure, nimodipine, and outcome of ischemic stroke. *Acta Neurol Scand* 2004; 109: 200-204.

14. Eveson DJ, Robinson TG and Potter JF. Lisinopril for the treatment of hypertension within the first 24 hours of acute ischemic stroke and follow-up. *Am J Hypertens* 2007; 20: 270-277.

15. Potter JF, Robinson TG, Ford GA, et al. Controlling hypertension and hypotension immediately post-stroke (CHHIPS): a randomised, placebo-controlled, double-blind pilot trial. *Lancet Neurol* 2009; 8: 48-56.

16. Bath PM, Martin RH, Palesch Y, et al. Effect of telmisartan on functional outcome, recurrence, and blood pressure in patients with acute mild ischemic stroke: a PRoFESS subgroup analysis. *Stroke* 2009; 40: 3541-3546.

17. He J, Zhang Y, Xu T, et al. Effects of immediate blood pressure reduction on death and major disability in patients with acute ischemic stroke: the CATIS randomized clinical trial. *Jama* 2014; 311: 479-489.

18. Efficacy of nitric oxide, with or without continuing antihypertensive treatment, for management of high blood pressure in acute stroke (ENOS): a partial-factorial randomised controlled trial. *Lancet* 2015; 385: 617-628.

19. Sandset EC, Bath PM, Boysen G, et al. The angiotensin-receptor blocker candesartan for treatment of acute stroke (SCAST): a randomised, placebo-controlled, double-blind trial. *Lancet* 2011; 377: 741-750.

20. Oh MS, Yu KH, Hong KS, et al. Modest blood pressure reduction with valsartan in acute ischemic stroke: a prospective, randomized, open-label, blinded-end-point trial. *Int J Stroke* 2015; 10: 745-751.

21. Li Y, Zhong Z, Luo S, et al. Efficacy of Antihypertensive Therapy in the Acute Stage of Cerebral Infarction - A Prospective, Randomized Control Trial. *Acta Cardiol Sin* 2018; 34: 502-510.

22. Yuan F, Yang F, Zhao J, et al. Controlling Hypertension After Severe Cerebrovascular Event (CHASE): A randomized, multicenter, controlled study. *Int J Stroke* 2021; 16: 456-465.

23. Anderson CS, Huang Y, Lindley RI, et al. Intensive blood pressure reduction with intravenous thrombolysis therapy for acute ischaemic stroke (ENCHANTED): an international, randomised, open-label, blinded-endpoint, phase 3 trial. *Lancet* 2019; 393: 877-888.

24. Bath PM, Woodhouse L, Krishnan K, et al. Effect of Treatment Delay, Stroke Type, and Thrombolysis on the Effect of Glyceryl Trinitrate, a Nitric Oxide Donor, on Outcome after Acute Stroke: A Systematic Review and Meta-Analysis of Individual Patient from Randomised Trials. *Stroke Res Treat* 2016; 2016: 9706720.

25. Yang P, Song L, Zhang Y, et al. Intensive blood pressure control after endovascular thrombectomy for acute ischaemic stroke (ENCHANTED2/MT): a multicentre, open-label, blinded-endpoint, randomised controlled trial. *Lancet* 2022; 400: 1585-1596.

26. Mazighi M, Richard S, Lapergue B, et al. Safety and efficacy of intensive blood pressure lowering after successful endovascular therapy in acute ischaemic stroke (BP-TARGET): a multicentre, open-label, randomised controlled trial. *Lancet Neurol* 2021; 20: 265-274.

27. Mistry EA, Hart KW, Davis LT, et al. Blood Pressure Management After Endovascular Therapy for Acute Ischemic Stroke: The BEST-II Randomized Clinical Trial. *Jama* 2023; 330: 821-831.

28. Katsanos AH, Catanese L, Sahlas DJ, et al. Blood Pressure Management Following Endovascular Stroke Treatment: A Feasibility Trial and Meta‐Analysis of Outcomes. *Stroke: Vascular and Interventional Neurology* 2024; 4: e001287.

29. Nam HS, Kim YD, Heo J, et al. Intensive vs Conventional Blood Pressure Lowering After Endovascular Thrombectomy in Acute Ischemic Stroke: The OPTIMAL-BP Randomized Clinical Trial. *Jama* 2023; 330: 832-842.

30. Bang OY, Chung JW, Kim SK, et al. Therapeutic-induced hypertension in patients with noncardioembolic acute stroke. *Neurology* 2019; 93: e1955-e1963.

31. Robinson TG, Potter JF, Ford GA, et al. Effects of antihypertensive treatment after acute stroke in the Continue or Stop Post-Stroke Antihypertensives Collaborative Study (COSSACS): a prospective, randomised, open, blinded-endpoint trial. *Lancet Neurol* 2010; 9: 767-775.

32. Koch S, Romano JG, Forteza AM, et al. Rapid blood pressure reduction in acute intracerebral hemorrhage: feasibility and safety. *Neurocrit Care* 2008; 8: 316-321.

33. Anderson CS, Huang Y, Wang JG, et al. Intensive blood pressure reduction in acute cerebral haemorrhage trial (INTERACT): a randomised pilot trial. *Lancet Neurol* 2008; 7: 391-399.

34. Anderson CS, Heeley E, Huang Y, et al. Rapid blood-pressure lowering in patients with acute intracerebral hemorrhage. *N Engl J Med* 2013; 368: 2355-2365.

35. Butcher KS, Jeerakathil T, Hill M, et al. The Intracerebral Hemorrhage Acutely Decreasing Arterial Pressure Trial. *Stroke* 2013; 44: 620-626.

36. Qureshi AI, Palesch YY, Barsan WG, et al. Intensive Blood-Pressure Lowering in Patients with Acute Cerebral Hemorrhage. *N Engl J Med* 2016; 375: 1033-1043.

37. Krishnan K, Scutt P, Woodhouse L, et al. Glyceryl Trinitrate for Acute Intracerebral Hemorrhage: Results From the Efficacy of Nitric Oxide in Stroke (ENOS) Trial, a Subgroup Analysis. *Stroke* 2016; 47: 44-52.

38. Zheng J, Li H, Lin S, et al. Perioperative Antihypertensive Treatment in Patients With Spontaneous Intracerebral Hemorrhage. *Stroke* 2017; 48: 216-218.

39. Gupta S, Abbot AK, Srinath R, et al. Randomized trial to assess safety and clinical efficacy of intensive blood pressure reduction in acute spontaneous intracerebral haemorrhage. *Med J Armed Forces India* 2018; 74: 120-125.

40. Dong R, Li F, Li B, et al. Effects of an Early Intensive Blood Pressure-lowering Strategy Using Remifentanil and Dexmedetomidine in Patients with Spontaneous Intracerebral Hemorrhage: A Multicenter, Prospective, Superiority, Randomized Controlled Trial. *Anesthesiology* 2024; 141: 100-115.

41. Butcher KS, Buck B, Dowlatshahi D, et al. Acute Blood Pressure Lowering and Risk of Ischemic Lesions on MRI After Intracerebral Hemorrhage. *JAMA Neurol* 2025; 82: 543-550.

42. Krishnan K, Scutt P, Woodhouse L, et al. Continuing versus Stopping Prestroke Antihypertensive Therapy in Acute Intracerebral Hemorrhage: A Subgroup Analysis of the Efficacy of Nitric Oxide in Stroke Trial. *J Stroke Cerebrovasc Dis* 2016; 25: 1017-1026.
