# Supplementary material for: 2025 update to European Stroke Organisation (ESO) guideline on blood pressure management in acute ischaemic stroke and intracerebral haemorrhage
Source: Eur Stroke J. 2026 May 7;11(5):aakag004. doi: 10.1093/esj/aakag004 (PMC13151662; doi:10.1093/esj/aakag004)
Supplement: BP_Management_Search_LH_111125_for_supplement_aakag004 [file bp_management_search_lh_111125_for_supplement_aakag004.docx]

Search strategies for the PICO questions

[PICO 1. In patients with suspected acute stroke, does pre-hospital blood pressure lowering with any vasodepressor drug compared to no drug improve outcome? 2](#_Toc182226220)

[PICO 2. In hospitalised patients with acute ischaemic stroke not treated with reperfusion therapies (intravenous thrombolysis or mechanical thrombectomy), does blood pressure lowering with any vasodepressor drug compared to no drug improve outcome? 9](#_Toc182226221)

[PICO 3. In hospitalised patients with acute ischaemic stroke and undergoing intravenous thrombolysis (with or without mechanical thrombectomy), does blood lowering therapies compared to control improve outcome? 9](#_Toc182226222)

[PICO 4. In patients with acute ischaemic stroke caused by large vessel occlusion and undergoing mechanical thrombectomy (with or without intravenous thrombolysis), does blood pressure lowering with any vasodepressor drug compared to no drug improve outcome? 9](#_Toc182226223)

[PICO 5. In patients with acute ischaemic stroke not treated with reperfusion therapies (intravenous thrombolysis or mechanical thrombectomy) and with clinical deterioration, does induced hypertension by any vasopressor drug compared to no drug improve outcome? 17](#_Toc182226224)

[PICO 6. In patients with acute ischaemic stroke, does continuing versus temporarily stopping previous oral blood pressure lowering therapy improve outcome? 21](#_Toc182226225)

[PICO 7. In patients with acute intracerebral haemorrhage, does intensive blood pressure lowering with any vasodepressor drug compared to control improve outcome? 28](#_Toc182226226)

[PICO 8. In patients with acute intracerebral haemorrhage, does continuing versus temporarily stopping previous oral antihypertensive therapy improve outcome? 34](#_Toc182226227)

# PICO 1. In patients with suspected acute stroke, does pre-hospital blood pressure lowering with any vasodepressor drug compared to no drug improve outcome?

Medline (Ovid)

| # | Query |
| --- | --- |
| 1 | exp cerebrovascular disorders/ or exp basal ganglia cerebrovascular disease/ or exp brain ischemia/ or exp carotid artery diseases/ or exp cerebrovascular trauma/ or exp intracranial arterial diseases/ or exp intracranial arteriovenous malformations/ or exp intracranial embolism/ or exp intracranial thrombosis/ or exp intracranial hemorrhages/ or exp stroke/ or exp brain infarction/ or vasospasm, intracranial/ or vertebral artery dissection/ or exp transient ischemic attack/ |
| 2 | (stroke or cerebrovasc* or brain vasc* or cerebral vasc* or cva* or apoplex* or large vessel occlusion or transient isch?emic attack or ICH or hemiparesis or hemiplegia or intracerebral arteriosclerosis).tw. |
| 3 | ((brain* or cerebr* or cerebell* or vertebrobasilar or hemispher* or intracran* or intracerebral or infratentorial or supratentorial or mca or anterior circulation or posterior circulation or basal ganglia) adj5 (isch?emi* or infarct* or thrombo* or emboli*)).tw. |
| 4 | or/1-3 |
| 5 | exp antihypertensive agents/ or exp vasodilator agents/ or exp adrenergic agonists/ or exp diuretics/ or exp thiazides/ or exp sodium chloride symporter inhibitors/ or exp sodium potassium chloride symporter inhibitors/ |
| 6 | exp angiotensin-converting enzyme inhibitors/ or exp angiotensin II type 1 receptor blockers/ or exp calcium channel blockers/ or exp adrenergic beta-antagonists/ or exp adrenergic alpha antagonists/ |
| 7 | exp enalapril/ or exp losartan/ or exp hydralazine/ |
| 8 | exp hypertension/ or exp blood pressure/ |
| 9 | (antihyperten* or anti-hypertens*).tw. |
| 10 | ((Blood pressure or hypertens*) adj5 (lower* or reduc* or decreas*)).tw. |
| 11 | (angiotensin adj3 convert* adj3 enzyme adj3 (inhibit* or antagonist? or block*)).tw. |
| 12 | (((ace or renin) adj3 inhibit*) or ACEI).tw. |
| 13 | (angiotensin adj3 receptor? adj3 (inhibit* or antagonist? or block*)).tw. |
| 14 | (calcium adj2 (inhibit* or antagonist? or block*)).tw. |
| 15 | (adrenergic adj3 beta* adj3 (inhibit* or antagonist? or block*)).tw. |
| 16 | (adrenergic adj3 alpha* adj3 (inhibit* or antagonist? or block*)).tw. |
| 17 | ((loop or ceiling) adj diuretic?).tw. |
| 18 | (amiloride or benzothiadiazine or bendroflumethiazide or bumetanide or chlorothiazide or cyclopenthiazide or furosemide or hydrochlorothiazide or hydroflumethiazide or methyclothiazide or metolazone or polythiazide or trichlormethiazide or veratide or thiazide?).mp. |
| 19 | (chlorthalidone or chlortalidone or phthalamudine or chlorphthalidolone or oxodoline or thalitone or hygroton or indapamide or metindamide or s-1520 or s1520 or se-1520 or se1520).mp. |
| 20 | (alacepril or altiopril or benazepril or captopril or ceronapril or cilazapril or delapril or enalapril or fosinopril or idapril or imidapril or lisinopril or moexipril or moveltipril or pentopril or perindopril or quinapril or ramipril or spirapril or temocapril or trandolapril or zofenopril or aliskiren or remikiren).mp. |
| 21 | (KT3-671 or candesartan or eprosartan or irbesartan or losartan or olmesartan or tasosartan or telmisartan or valsartan).mp. |
| 22 | (amlodipine or amrinone or bencyclane or bepridil or cinnarizine or conotoxins or diltiazem or felodipine or fendiline or flunarizine or gallopamil or isradipine or lidoflazine or magnesium sulfate or mibefradil or nicardipine or nifedipine or nimodipine or nisoldipine or nitrendipine or perhexiline or prenylamine or verapamil or omega-agatoxin iva or omega-conotoxin gvia or omega-conotoxins).mp. |
| 23 | (methyldopa or alphamethyldopa or amodopa or dopamet or dopegyt or dopegit or dopegite or emdopa or hyperpax or hyperpaxa or methylpropionic acid or dopergit or meldopa or methyldopate or medopa or medomet or sembrina or aldomet or aldometil or aldomin or hydopa or methyldihydroxyphenylalanine or methyl dopa or mulfasin or presinol or presolisin or sedometil or sembrina or taquinil or dihydroxyphenylalanine or methylphenylalanine or methylalanine or alpha methyl dopa).mp. |
| 24 | (reserpine or serpentina or rauwolfia or serpasil).mp. |
| 25 | (clonidine or adesipress or arkamin or caprysin or catapres* or catasan or chlofazolin or chlophazolin or clinidine or clofelin* or clofenil or clomidine or clondine or clonistada or clonnirit or clophelin* or dichlorophenylaminoimidazoline or dixarit or duraclon or gemiton or haemiton or hemiton or imidazoline or isoglaucon or klofelin or klofenil or m-5041t or normopresan or paracefan or st-155 or st 155 or tesno timelets).mp. |
| 26 | (hydralazin* or hydrallazin* or hydralizine or hydrazinophtalazine or hydrazinophthalazine or hydrazinophtalizine or dralzine or hydralacin or hydrolazine or hypophthalin or hypoftalin or hydrazinophthalazine or idralazina or 1-hydrazinophthalazine or apressin or nepresol or apressoline or apresoline or apresolin or alphapress or alazine or idralazina or lopress or plethorit or praeparat).mp. |
| 27 | (acebutolol or adimolol or afurolol or alprenolol or amosulalol or arotinolol or atenolol or befunolol or betaxolol or bevantolol or bisoprolol or bopindolol or bornaprolol or brefonalol or bucindolol or bucumolol or bufetolol or bufuralol or bunitrolol or bunolol or bupranolol or butofilolol or butoxamine or carazolol or carteolol or carvedilol or celiprolol or cetamolol or chlortalidone cloranolol or cyanoiodopindolol or cyanopindolol or deacetylmetipranolol or diacetolol or dihydroalprenolol or dilevalol or epanolol or esmolol or exaprolol or falintolol or flestolol or flusoxolol or hydroxybenzylpinodolol or hydroxycarteolol or hydroxymetoprolol or indenolol or iodocyanopindolol or iodopindolol or iprocrolol or isoxaprolol or labetalol or landiolol or levobunolol or levomoprolol or medroxalol or mepindolol or methylthiopropranolol or metipranolol or metoprolol or moprolol or nadolol or oxprenolol or penbutolol or pindolol or nadolol or nebivolol or nifenalol or nipradilol or oxprenolol or pafenolol or pamatolol or penbutolol or pindolol or practolol or primidolol or prizidilol or procinolol or pronetalol or propranolol or proxodolol or ridazolol or salcardolol or soquinolol or sotalol or spirendolol or talinolol or tertatolol or tienoxolol or tilisolol or timolol or tolamolol or toliprolol or tribendilol or xibenolol).mp. |
| 28 | (alfuzosin or bunazosin or doxazosin or metazosin or neldazosin or prazosin or silodosin or tamsulosin or terazosin or tiodazosin or trimazosin).mp. |
| 29 | or/5-28 |
| 30 | exp ambulance/ or exp air ambulances/ or exp Emergency Medical Services/ or exp Emergency Medical Technicians/ or exp Emergency Service, Hospital/ or exp Emergency Medicine/ or exp "Transportation of Patients"/ or exp early medical intervention/ |
| 31 | (helicopter emergency medical service* or HEMS).tw. |
| 32 | (paramedic* or prehospital or pre-hospital or ambulance or emergency service? or emergency medical service? or emergency technician? or early treatment or early intervention).tw. |
| 33 | or/30-32 |
| 34 | 4 or 29 or 33 |
| 35 | exp randomized controlled trial/ |
| 36 | controlled clinical trial.pt. |
| 37 | randomized.ab. |
| 38 | placebo.ab. |
| 39 | drug therapy.fs. |
| 40 | randomly.ab. |
| 41 | trial.ab. |
| 42 | groups.ab. |
| 43 | or/35-42 |
| 44 | 34 and 43 |
| 45 | exp animals/ not humans.sh. |
| 46 | (cardi* or myocard* or heart or coronary).tw. |
| 47 | exp child/ or adolescent/ or exp infant/ or exp Pediatrics/ or child*.mp. or p?ediat*.mp. or neonat*.mp. or newborn*.mp. or infant*.mp. or baby*.mp. or babies.mp. or toddler*.mp. or minors*.mp. or adolesc*.mp. or preteen*.mp. or teen*.mp. or juvenil*.mp. or youth*.mp. or preschool*.mp. or school*.mp. or kindergarten*.mp. or kid.mp. or kids.mp. |
| 48 | or/45-47 |
| 49 | 44 not 48 |

Embase (Ovid)

| # | Query |
| --- | --- |
| 1 | exp cerebrovascular disorders/ or exp basal ganglia cerebrovascular disease/ or exp brain ischemia/ or exp carotid artery diseases/ or exp cerebrovascular trauma/ or exp intracranial arterial diseases/ or exp intracranial arteriovenous malformations/ or exp intracranial embolism/ or exp intracranial thrombosis/ or exp intracranial hemorrhages/ or exp stroke/ or exp brain infarction/ or vasospasm, intracranial/ or vertebral artery dissection/ or exp transient ischemic attack/ |
| 2 | (stroke or cerebrovasc* or brain vasc* or cerebral vasc* or cva* or apoplex* or large vessel occlusion or transient isch?emic attack or ICH or hemiparesis or hemiplegia or intracerebral arteriosclerosis).tw. |
| 3 | ((brain* or cerebr* or cerebell* or vertebrobasilar or hemispher* or intracran* or intracerebral or infratentorial or supratentorial or mca or anterior circulation or posterior circulation or basal ganglia) adj5 (isch?emi* or infarct* or thrombo* or emboli*)).tw. |
| 4 | or/1-3 |
| 5 | (acute or sudden or spontaneous).mp. |
| 6 | 4 and 5 |
| 7 | exp antihypertensive agents/ or exp vasodilator agents/ or exp adrenergic agonists/ or exp diuretics/ or exp thiazides/ or exp sodium chloride symporter inhibitors/ or exp sodium potassium chloride symporter inhibitors/ |
| 8 | exp angiotensin-converting enzyme inhibitors/ or exp angiotensin II type 1 receptor blockers/ or exp calcium channel blockers/ or exp adrenergic beta-antagonists/ or exp adrenergic alpha antagonists/ |
| 9 | exp enalapril/ or exp losartan/ or exp hydralazine/ |
| 10 | hypertension/ae, de, dt, pc or blood pressure/de, pd |
| 11 | (antihyperten* or anti-hypertens*).tw. |
| 12 | ((Blood pressure or hypertens*) adj5 (lower* or reduc* or decreas*)).tw. |
| 13 | (angiotensin adj3 convert* adj3 enzyme adj3 (inhibit* or antagonist? or block*)).tw. |
| 14 | (((ace or renin) adj3 inhibit*) or ACEI).tw. |
| 15 | (angiotensin adj3 receptor? adj3 (inhibit* or antagonist? or block*)).tw. |
| 16 | (calcium adj2 (inhibit* or antagonist? or block*)).tw. |
| 17 | (adrenergic adj3 beta* adj3 (inhibit* or antagonist? or block*)).tw. |
| 18 | (adrenergic adj3 alpha* adj3 (inhibit* or antagonist? or block*)).tw. |
| 19 | ((loop or ceiling) adj diuretic?).tw. |
| 20 | (amiloride or benzothiadiazine or bendroflumethiazide or bumetanide or chlorothiazide or cyclopenthiazide or furosemide or hydrochlorothiazide or hydroflumethiazide or methyclothiazide or metolazone or polythiazide or trichlormethiazide or veratide or thiazide?).mp. |
| 21 | (chlorthalidone or chlortalidone or phthalamudine or chlorphthalidolone or oxodoline or thalitone or hygroton or indapamide or metindamide or s-1520 or s1520 or se-1520 or se1520).mp. |
| 22 | (alacepril or altiopril or benazepril or captopril or ceronapril or cilazapril or delapril or enalapril or fosinopril or idapril or imidapril or lisinopril or moexipril or moveltipril or pentopril or perindopril or quinapril or ramipril or spirapril or temocapril or trandolapril or zofenopril or aliskiren or remikiren).mp. |
| 23 | (KT3-671 or candesartan or eprosartan or irbesartan or losartan or olmesartan or tasosartan or telmisartan or valsartan).mp. |
| 24 | (amlodipine or amrinone or bencyclane or bepridil or cinnarizine or conotoxins or diltiazem or felodipine or fendiline or flunarizine or gallopamil or isradipine or lidoflazine or magnesium sulfate or mibefradil or nicardipine or nifedipine or nimodipine or nisoldipine or nitrendipine or perhexiline or prenylamine or verapamil or omega-agatoxin iva or omega-conotoxin gvia or omega-conotoxins).mp. |
| 25 | (methyldopa or alphamethyldopa or amodopa or dopamet or dopegyt or dopegit or dopegite or emdopa or hyperpax or hyperpaxa or methylpropionic acid or dopergit or meldopa or methyldopate or medopa or medomet or sembrina or aldomet or aldometil or aldomin or hydopa or methyldihydroxyphenylalanine or methyl dopa or mulfasin or presinol or presolisin or sedometil or sembrina or taquinil or dihydroxyphenylalanine or methylphenylalanine or methylalanine or alpha methyl dopa).mp. |
| 26 | (reserpine or serpentina or rauwolfia or serpasil).mp. |
| 27 | (clonidine or adesipress or arkamin or caprysin or catapres* or catasan or chlofazolin or chlophazolin or clinidine or clofelin* or clofenil or clomidine or clondine or clonistada or clonnirit or clophelin* or dichlorophenylaminoimidazoline or dixarit or duraclon or gemiton or haemiton or hemiton or imidazoline or isoglaucon or klofelin or klofenil or m-5041t or normopresan or paracefan or st-155 or st 155 or tesno timelets).mp. |
| 28 | (hydralazin* or hydrallazin* or hydralizine or hydrazinophtalazine or hydrazinophthalazine or hydrazinophtalizine or dralzine or hydralacin or hydrolazine or hypophthalin or hypoftalin or hydrazinophthalazine or idralazina or 1-hydrazinophthalazine or apressin or nepresol or apressoline or apresoline or apresolin or alphapress or alazine or idralazina or lopress or plethorit or praeparat).mp. |
| 29 | (acebutolol or adimolol or afurolol or alprenolol or amosulalol or arotinolol or atenolol or befunolol or betaxolol or bevantolol or bisoprolol or bopindolol or bornaprolol or brefonalol or bucindolol or bucumolol or bufetolol or bufuralol or bunitrolol or bunolol or bupranolol or butofilolol or butoxamine or carazolol or carteolol or carvedilol or celiprolol or cetamolol or chlortalidone cloranolol or cyanoiodopindolol or cyanopindolol or deacetylmetipranolol or diacetolol or dihydroalprenolol or dilevalol or epanolol or esmolol or exaprolol or falintolol or flestolol or flusoxolol or hydroxybenzylpinodolol or hydroxycarteolol or hydroxymetoprolol or indenolol or iodocyanopindolol or iodopindolol or iprocrolol or isoxaprolol or labetalol or landiolol or levobunolol or levomoprolol or medroxalol or mepindolol or methylthiopropranolol or metipranolol or metoprolol or moprolol or nadolol or oxprenolol or penbutolol or pindolol or nadolol or nebivolol or nifenalol or nipradilol or oxprenolol or pafenolol or pamatolol or penbutolol or pindolol or practolol or primidolol or prizidilol or procinolol or pronetalol or propranolol or proxodolol or ridazolol or salcardolol or soquinolol or sotalol or spirendolol or talinolol or tertatolol or tienoxolol or tilisolol or timolol or tolamolol or toliprolol or tribendilol or xibenolol).mp. |
| 30 | (alfuzosin or bunazosin or doxazosin or metazosin or neldazosin or prazosin or silodosin or tamsulosin or terazosin or tiodazosin or trimazosin).mp. |
| 31 | or/7-30 |
| 32 | 6 and 31 |
| 33 | exp ambulance/ or exp air ambulances/ or exp Emergency Medical Services/ or exp Emergency Medical Technicians/ or exp Emergency Service, Hospital/ or exp Emergency Medicine/ or exp "Transportation of Patients"/ or exp early medical intervention/ |
| 34 | (helicopter emergency medical service* or HEMS).mp. |
| 35 | (paramedic* or prehospital or pre-hospital or ambulance or emergency service? or emergency medical service? or emergency technician? or early treatment or early intervention).mp. |
| 36 | or/33-35 |
| 37 | 32 and 36 |
| 38 | exp randomized controlled trial/ |
| 39 | controlled clinical trial/ |
| 40 | random*.ti,ab. |
| 41 | randomization/ |
| 42 | intermethod comparison/ |
| 43 | placebo.ti,ab. |
| 44 | (compare or compared or comparison).ti,ab. |
| 45 | ((evaluated or evaluate or evaluating or assessed or assess) and (compare or compared or comparing or comparison)).ab. |
| 46 | (open adj label).ti,ab. |
| 47 | ((double or single or doubly or singly) adj (blind or blinded or blindly)).ti,ab. |
| 48 | double blind procedure/ |
| 49 | parallel group*1.ti,ab. |
| 50 | (crossover or cross over).ti,ab. |
| 51 | ((assign* or match or matched or allocation) adj5 (alternate or group*1 or intervention*1 or patient*1 or subject*1 or participant*1)).ti,ab. |
| 52 | (assigned or allocated).ti,ab. |
| 53 | (controlled adj7 (study or design or trial)).ti,ab. |
| 54 | (volunteer or volunteers).ti,ab. |
| 55 | human experiment/ |
| 56 | trial.ti. |
| 57 | or/38-56 |
| 58 | (random* adj sampl* adj7 ("cross section*" or questionnaire*1 or survey* or database*1)).ti,ab. not (comparative study/ or controlled study/ or randomi?ed controlled.ti,ab. or randomly assigned.ti,ab.) |
| 59 | cross-sectional study/ not (exp randomized controlled trial/ or controlled clinical trial/ or controlled study/ or randomi?ed controlled.ti,ab. or control group*1.ti,ab.) |
| 60 | (((case adj control*) and random*) not randomi?ed controlled).ti,ab. |
| 61 | systematic review.ti,ab. not (trial or study).ti. |
| 62 | (non random* not random*).ti,ab. |
| 63 | "random field*".ti,ab. |
| 64 | (random cluster adj3 sample*).ti,ab. |
| 65 | (review.ab. and review.pt.) not trial.ti. |
| 66 | "we searched".ab. and (review.ti. or review.pt.) |
| 67 | "update review".ab. |
| 68 | (databases adj4 searched).ab. |
| 69 | (rat or rats or mouse or mice or swine or porcine or murine or sheep or lambs or pigs or piglets or rabbit or rabbits or cat or cats or dog or dogs or cattle or bovine or monkey or monkeys or trout or marmoset*1).ti. and animal experiment/ |
| 70 | animal experiment/ not (human experiment/ or human/) |
| 71 | (cardi* or myocard* or heart or coronary).tw. |
| 72 | exp child/ or adolescent/ or exp infant/ or exp Pediatrics/ or child*.mp. or p?ediat*.mp. or neonat*.mp. or newborn*.mp. or infant*.mp. or baby*.mp. or babies.mp. or toddler*.mp. or minors*.mp. or adolesc*.mp. or preteen*.mp. or teen*.mp. or juvenil*.mp. or youth*.mp. or preschool*.mp. or school*.mp. or kindergarten*.mp. or kid.mp. or kids.mp. |
| 73 | or/58-72 |
| 74 | 57 not 73 |
| 75 | 37 and 74 |

Cochrane Library

| # | Query |
| --- | --- |
| 1 | [mh "cerebrovascular disorders"] or [mh "basal ganglia cerebrovascular disease"] or [mh "brain ischemia"] or [mh "carotid artery diseases"] or [mh "cerebrovascular trauma"] or [mh "intracranial arterial diseases"] or [mh "intracranial arteriovenous malformations"] or [mh "intracranial embolism"] or [mh "intracranial thrombosis"] or [mh "intracranial hemorrhages"] or [mh stroke] or [mh "brain infarction"] or [mh "vasospasm, intracranial"] or [mh "vertebral artery dissection"] |
| 2 | (stroke or cerebrovasc* or brain next vasc* or cerebral next vasc* or cva* or apoplex*):ti,ab,kw |
| 3 | ((brain* or cerebr* or cerebell* or vertebrobasilar or hemispher* or intracran* or intracerebral or infratentorial or supratentorial or mca or anterior next circulation or posterior next circulation or basal next ganglia) near/5 (ischemi* or ischaemi* or infarct* or thrombo* or emboli*)):ti,ab,kw |
| 4 | ((brain* or cerebr* or cerebell* or intracerebral or intracran* or parenchymal or intraventricular or infratentorial or supratentorial or basal next gangli*) near/5 (haemorrhage* or hemorrhage* or haematoma* or hematoma* or bleed*)):ti,ab,kw |
| 5 | {or #1-#4} |
| 6 | [mh "antihypertensive agents"] or [mh "vasodilator agents"] or [mh "adrenergic agonists"] or [mh "diuretics"] or [mh "thiazides"] or [mh "sodium chloride symporter inhibitors"] or [mh "sodium potassium chloride symporter inhibitors"] or [mh "angiotensin-converting enzyme inhibitors"] or [mh "angiotensin ii type 1 receptor blockers"] or [mh "calcium channel blockers"] or [mh "adrenergic beta-antagonists"] or [mh "adrenergic alpha-antagonists"] or [mh "enalapril"] or [mh "losartan"] or [mh "hydralazine"] |
| 7 | MeSH descriptor: [Hypertension] this term only and with qualifier(s): [drug therapy - DT, prevention & control - PC] |
| 8 | MeSH descriptor: [Blood Pressure] this term only and with qualifier(s): [drug effects - DE] |
| 9 | ((antihypertens* or anti-hypertens*)):ti,ab,kw |
| 10 | ((("blood pressure" or hypertens*) near/5 (lower* or reduc* or decreas*))):ti,ab,kw |
| 11 | ((angiotensin near/3 convert* near/3 enzyme near/3 (inhibit* or antagonist* or block*))):ti,ab,kw |
| 12 | ((((ace or renin) near/3 inhibit*) or ACEI)):ti,ab,kw |
| 13 | ((angiotensin near/3 receptor* near/3 (inhibit* or antagonist* or block*))):ti,ab,kw |
| 14 | ((calcium near/2 (inhibit* or antagonist* or block*))):ti,ab,kw |
| 15 | ((adrenergic near/3 beta* near/3 (inhibit* or antagonist* or block*))):ti,ab,kw |
| 16 | ((adrenergic near/3 alpha* near/3 (inhibit* or antagonist* or block*))):ti,ab,kw |
| 17 | (((loop or ceiling) next diuretic*)):ti,ab,kw |
| 18 | ((amiloride or benzothiadiazine or bendroflumethiazide or bumetanide or chlorothiazide or cyclopenthiazide or furosemide or hydrochlorothiazide or hydroflumethiazide or methyclothiazide or metolazone or polythiazide or trichlormethiazide or veratide or thiazide*)):ti,ab,kw |
| 19 | ((chlorthalidone or chlortalidone or phthalamudine or chlorphthalidolone or oxodoline or thalitone or hygroton or indapamide or metindamide or "s-1520" or s1520 or "se-1520" or se1520)):ti,ab,kw |
| 20 | ((alacepril or altiopril or benazepril or captopril or ceronapril or cilazapril or delapril or enalapril or fosinopril or idapril or imidapril or lisinopril or moexipril or moveltipril or pentopril or perindopril or quinapril or ramipril or spirapril or temocapril or trandolapril or zofenopril or aliskiren or remikiren)):ti,ab,kw |
| 21 | (("KT3-671" or candesartan or eprosartan or irbesartan or losartan or olmesartan or tasosartan or telmisartan or valsartan)):ti,ab,kw |
| 22 | ((amlodipine or amrinone or bencyclane or bepridil or cinnarizine or conotoxins or diltiazem or felodipine or fendiline or flunarizine or gallopamil or isradipine or lidoflazine or "magnesium sulphate" or mibefradil or nicardipine or nifedipine or nimodipine or nisoldipine or nitrendipine or perhexiline or prenylamine or verapamil or "omega-agatoxin iva" or "omega-conotoxin gvia" or "omega-conotoxins")):ti,ab,kw |
| 23 | ((methyldopa or alphamethyldopa or amodopa or dopamet or dopegyt or dopegit or dopegite or emdopa or hyperpax or hyperpaxa or "methylpropionic acid" or dopergit or meldopa or methyldopate or medopa or medomet or sembrina or aldomet or aldometil or aldomin or hydopa or methyldihydroxyphenylalanine or "methyl dopa" or mulfasin or presinol or presolisin or sedometil or sembrina or taquinil or dihydroxyphenylalanine or methylphenylalanine or methylalanine or "alpha methyl dopa")):ti,ab,kw |
| 24 | ((reserpine or serpentina or rauwolfia or serpasil)):ti,ab,kw |
| 25 | ((clonidine or adesipress or arkamin or caprysin or catapres* or catasan or chlofazolin or chlophazolin or clinidine or clofelin* or clofenil or clomidine or clondine or clonistada or clonnirit or clophelin* or dichlorophenylaminoimidazoline or dixarit or duraclon or gemiton or haemiton or hemiton or imidazoline or isoglaucon or klofelin or klofenil or "m-5041t" or normopresan or paracefan or "st-155" or "st 155" or "tesno timelets")):ti,ab,kw |
| 26 | ((hydralazin* or hydrallazin* or hydralizine or hydrazinophtalazine or hydrazinophthalazine or hydrazinophtalizine or dralzine or hydralacin or hydrolazine or hypophthalin or hypoftalin or hydrazinophthalazine or idralazina or "1-hydrazinophthalazine" or apressin or nepresol or apressoline or apresoline or apresolin or alphapress or alazine or idralazina or lopress or plethorit or praeparat)):ti,ab,kw |
| 27 | ((acebutolol or adimolol or afurolol or alprenolol or amosulalol or arotinolol or atenolol or befunolol or betaxolol or bevantolol or bisoprolol or bopindolol or bornaprolol or brefonalol or bucindolol or bucumolol or bufetolol or bufuralol or bunitrolol or bunolol or bupranolol or butofilolol or butoxamine or carazolol or carteolol or carvedilol or celiprolol or cetamolol or chlortalidone cloranolol or cyanoiodopindolol or cyanopindolol or deacetylmetipranolol or diacetolol or dihydroalprenolol or dilevalol or epanolol or esmolol or exaprolol or falintolol or flestolol or flusoxolol or hydroxybenzylpinodolol or hydroxycarteolol or hydroxymetoprolol or indenolol or iodocyanopindolol or iodopindolol or iprocrolol or isoxaprolol or labetalol or landiolol or levobunolol or levomoprolol or medroxalol or mepindolol or methylthiopropranolol or metipranolol or metoprolol or moprolol or nadolol or oxprenolol or penbutolol or pindolol or nadolol or nebivolol or nifenalol or nipradilol or oxprenolol or pafenolol or pamatolol or penbutolol or pindolol or practolol or primidolol or prizidilol or procinolol or pronetalol or propranolol or proxodolol or ridazolol or salcardolol or soquinolol or sotalol or spirendolol or talinolol or tertatolol or tienoxolol or tilisolol or timolol or tolamolol or toliprolol or tribendilol or xibenolol)):ti,ab,kw |
| 28 | ((alfuzosin or bunazosin or doxazosin or metazosin or neldazosin or prazosin or silodosin or tamsulosin or terazosin or tiodazosin or trimazosin)):ti,ab,kw |
| 29 | {or #6-#28} |
| 30 | #5 and #29 |
| 31 | [mh "emergency medical services"] or [mh "emergency medical service communication systems"] or [mh "emergency service, hospital"] or [mh "emergency medicine"] or [mh "emergency treatment"] or [mh "early medical intervention"] |
| 32 | [mh "ambulances"] or [mh "air ambulances"] or [mh "emergency responders"] or [mh "allied health personnel"] or [mh "transportation of patients"] |
| 33 | (prehospital* or pre‐hospital* or pre hospital* or ambulance* or helicopter next emergency next medical next service* or HEMS or paramedic* or EMS):ti,ab,kw |
| 34 | (paramedic* or prehospital or pre-hospital or ambulance or emergency service* or emergency medical service* or emergency technician* or early treatment or early intervention):ti,ab,kw |
| 35 | {or #31-#34} |
| 36 | #30 and #35 |
| 37 | [mh "infant, newborn"] or [mh "pediatrics"] or [mh "adolescent"] |
| 38 | (cardi* or myocard* or heart or coronary):ti,ab,kw |
| 39 | (child* or p?ediat* or neonat* or newborn* or infant* or baby* or babies or toddler* or minors* or adolesc* or preteen* or teen* or juvenil* or youth* or preschool* or school* or kindergarten* or kid*):ti,ab,kw |
| 40 | {or #37-#39} |
| 41 | #36 not #40 |

# PICO 2. In hospitalised patients with acute ischaemic stroke not treated with reperfusion therapies (intravenous thrombolysis or mechanical thrombectomy), does blood pressure lowering with any vasodepressor drug compared to no drug improve outcome?

# PICO 3. In hospitalised patients with acute ischaemic stroke and undergoing intravenous thrombolysis (with or without mechanical thrombectomy), does blood lowering therapies compared to control improve outcome?

# PICO 4. In patients with acute ischaemic stroke caused by large vessel occlusion and undergoing mechanical thrombectomy (with or without intravenous thrombolysis), does blood pressure lowering with any vasodepressor drug compared to no drug improve outcome?

Medline (Ovid)

| # | Query |
| --- | --- |
| 1 | exp cerebrovascular disorders/ or exp basal ganglia cerebrovascular disease/ or exp brain ischemia/ or exp carotid artery diseases/ or exp intracranial arterial diseases/ or exp intracranial embolism/ or exp intracranial thrombosis/ or exp stroke/ or exp brain infarction/ or exp transient ischemic attack/ |
| 2 | (stroke or cerebrovasc* or brain vasc* or cerebral vasc* or cva* or apoplex* or large vessel occlusion or transient isch?emic attack or hemiparesis or hemiplegia or intracerebral arteriosclerosis).tw. |
| 3 | ((brain* or cerebr* or cerebell* or vertebrobasilar or hemispher* or intracran* or intracerebral or infratentorial or supratentorial or mca or anterior circulation or posterior circulation or basal ganglia) adj5 (isch?emi* or infarct* or thrombo* or emboli*)).tw. |
| 4 | or/1-3 |
| 5 | exp antihypertensive agents/ or exp vasodilator agents/ or exp adrenergic agonists/ or exp diuretics/ or exp thiazides/ or exp sodium chloride symporter inhibitors/ or exp sodium potassium chloride symporter inhibitors/ |
| 6 | exp angiotensin-converting enzyme inhibitors/ or exp angiotensin II type 1 receptor blockers/ or exp calcium channel blockers/ or exp adrenergic beta-antagonists/ or exp adrenergic alpha antagonists/ |
| 7 | exp enalapril/ or exp losartan/ or exp hydralazine/ |
| 8 | exp hypertension/ or exp blood pressure/ |
| 9 | (antihyperten* or anti-hypertens*).tw. |
| 10 | ((Blood pressure or hypertens*) adj5 (lower* or reduc* or decreas*)).tw. |
| 11 | (angiotensin adj3 convert* adj3 enzyme adj3 (inhibit* or antagonist? or block*)).tw. |
| 12 | (((ace or renin) adj3 inhibit*) or ACEI).tw. |
| 13 | (angiotensin adj3 receptor? adj3 (inhibit* or antagonist? or block*)).tw. |
| 14 | (calcium adj2 (inhibit* or antagonist? or block*)).tw. |
| 15 | (adrenergic adj3 beta* adj3 (inhibit* or antagonist? or block*)).tw. |
| 16 | (adrenergic adj3 alpha* adj3 (inhibit* or antagonist? or block*)).tw. |
| 17 | ((loop or ceiling) adj diuretic?).tw. |
| 18 | (amiloride or benzothiadiazine or bendroflumethiazide or bumetanide or chlorothiazide or cyclopenthiazide or furosemide or hydrochlorothiazide or hydroflumethiazide or methyclothiazide or metolazone or polythiazide or trichlormethiazide or veratide or thiazide?).mp. |
| 19 | (chlorthalidone or chlortalidone or phthalamudine or chlorphthalidolone or oxodoline or thalitone or hygroton or indapamide or metindamide or s-1520 or s1520 or se-1520 or se1520).mp. |
| 20 | (alacepril or altiopril or benazepril or captopril or ceronapril or cilazapril or delapril or enalapril or fosinopril or idapril or imidapril or lisinopril or moexipril or moveltipril or pentopril or perindopril or quinapril or ramipril or spirapril or temocapril or trandolapril or zofenopril or aliskiren or remikiren).mp. |
| 21 | (KT3-671 or candesartan or eprosartan or irbesartan or losartan or olmesartan or tasosartan or telmisartan or valsartan).mp. |
| 22 | (amlodipine or amrinone or bencyclane or bepridil or cinnarizine or conotoxins or diltiazem or felodipine or fendiline or flunarizine or gallopamil or isradipine or lidoflazine or magnesium sulfate or mibefradil or nicardipine or nifedipine or nimodipine or nisoldipine or nitrendipine or perhexiline or prenylamine or verapamil or omega-agatoxin iva or omega-conotoxin gvia or omega-conotoxins).mp. |
| 23 | (methyldopa or alphamethyldopa or amodopa or dopamet or dopegyt or dopegit or dopegite or emdopa or hyperpax or hyperpaxa or methylpropionic acid or dopergit or meldopa or methyldopate or medopa or medomet or sembrina or aldomet or aldometil or aldomin or hydopa or methyldihydroxyphenylalanine or methyl dopa or mulfasin or presinol or presolisin or sedometil or sembrina or taquinil or dihydroxyphenylalanine or methylphenylalanine or methylalanine or alpha methyl dopa).mp. |
| 24 | (reserpine or serpentina or rauwolfia or serpasil).mp. |
| 25 | (clonidine or adesipress or arkamin or caprysin or catapres* or catasan or chlofazolin or chlophazolin or clinidine or clofelin* or clofenil or clomidine or clondine or clonistada or clonnirit or clophelin* or dichlorophenylaminoimidazoline or dixarit or duraclon or gemiton or haemiton or hemiton or imidazoline or isoglaucon or klofelin or klofenil or m-5041t or normopresan or paracefan or st-155 or st 155 or tesno timelets).mp. |
| 26 | (hydralazin* or hydrallazin* or hydralizine or hydrazinophtalazine or hydrazinophthalazine or hydrazinophtalizine or dralzine or hydralacin or hydrolazine or hypophthalin or hypoftalin or hydrazinophthalazine or idralazina or 1-hydrazinophthalazine or apressin or nepresol or apressoline or apresoline or apresolin or alphapress or alazine or idralazina or lopress or plethorit or praeparat).mp. |
| 27 | (acebutolol or adimolol or afurolol or alprenolol or amosulalol or arotinolol or atenolol or befunolol or betaxolol or bevantolol or bisoprolol or bopindolol or bornaprolol or brefonalol or bucindolol or bucumolol or bufetolol or bufuralol or bunitrolol or bunolol or bupranolol or butofilolol or butoxamine or carazolol or carteolol or carvedilol or celiprolol or cetamolol or chlortalidone cloranolol or cyanoiodopindolol or cyanopindolol or deacetylmetipranolol or diacetolol or dihydroalprenolol or dilevalol or epanolol or esmolol or exaprolol or falintolol or flestolol or flusoxolol or hydroxybenzylpinodolol or hydroxycarteolol or hydroxymetoprolol or indenolol or iodocyanopindolol or iodopindolol or iprocrolol or isoxaprolol or labetalol or landiolol or levobunolol or levomoprolol or medroxalol or mepindolol or methylthiopropranolol or metipranolol or metoprolol or moprolol or nadolol or oxprenolol or penbutolol or pindolol or nadolol or nebivolol or nifenalol or nipradilol or oxprenolol or pafenolol or pamatolol or penbutolol or pindolol or practolol or primidolol or prizidilol or procinolol or pronetalol or propranolol or proxodolol or ridazolol or salcardolol or soquinolol or sotalol or spirendolol or talinolol or tertatolol or tienoxolol or tilisolol or timolol or tolamolol or toliprolol or tribendilol or xibenolol).mp. |
| 28 | (alfuzosin or bunazosin or doxazosin or metazosin or neldazosin or prazosin or silodosin or tamsulosin or terazosin or tiodazosin or trimazosin).mp. |
| 29 | or/5-28 |
| 30 | 4 and 29 |
| 31 | thrombolytic therapy/ |
| 32 | fibrinolytic agents/ or fibrinolysin/ or plasminogen/ or tissue plasminogen activator/ or exp plasminogen activators/ or urokinase-type plasminogen activator/ |
| 33 | fibrinolysis/ |
| 34 | (thromboly? or fibrinoly? or recanalis? or recanaliz?).mp. |
| 35 | ((clot? or thrombus) adj5 (lyse or lysis or dissolve? or dissolution)).mp. |
| 36 | (tPA or t-PA or rtPA or rt-PA or plasminogen or plasmin or alteplase or actilyse).mp. |
| 37 | (anistreplase or streptodornase or streptokinase or urokinase or pro?urokinase or rpro?uk or lumbrokinase or duteplase or lanoteplase or pamiteplase or reteplase or saruplase or staphylokinase or streptase or tenecteplase).mp. |
| 38 | or/31-37 |
| 39 | (endovascular adj (therap* or treatment? or procedure? or method? or stent* or adverse effect? thromb* or embol*)).mp. |
| 40 | ((intra-arterial or intraarterial or mechanical or rheolytic) adj (thromb* or embol*)).mp. |
| 41 | (angiojet or stent retriever or trevo retriever or penumbra device or solitaire revasculari?ation or balloon angioplasty).mp. |
| 42 | or/39-41 |
| 43 | 38 or 42 |
| 44 | 30 and 43 |
| 45 | exp randomized controlled trial/ |
| 46 | controlled clinical trial.pt. |
| 47 | randomized.ab. |
| 48 | placebo.ab. |
| 49 | drug therapy.fs. |
| 50 | randomly.ab. |
| 51 | trial.ab. |
| 52 | groups.ab. |
| 53 | or/45-52 |
| 54 | 44 and 53 |
| 55 | exp animals/ not humans.sh. |
| 56 | (cardi* or myocard* or heart or coronary).tw. |
| 57 | exp child/ or adolescent/ or exp infant/ or exp Pediatrics/ or child*.mp. or p?ediat*.mp. or neonat*.mp. or newborn*.mp. or infant*.mp. or baby*.mp. or babies.mp. or toddler*.mp. or minors*.mp. or adolesc*.mp. or preteen*.mp. or teen*.mp. or juvenil*.mp. or youth*.mp. or preschool*.mp. or school*.mp. or kindergarten*.mp. or kid.mp. or kids.mp |
| 58 | or/55-57 |
| 59 | 54 not 58 |

Embase (Ovid)

| # | Query |
| --- | --- |
| 1 | exp cerebrovascular disorders/ or exp basal ganglia cerebrovascular disease/ or exp brain ischemia/ or exp carotid artery diseases/ or exp intracranial arterial diseases/ or exp intracranial embolism/ or exp intracranial thrombosis/ or exp stroke/ or exp brain infarction/ or exp transient ischemic attack/ |
| 2 | (stroke or cerebrovasc* or brain vasc* or cerebral vasc* or cva* or apoplex* or large vessel occlusion or transient isch?emic attack or hemiparesis or hemiplegia or intracerebral arteriosclerosis).tw. |
| 3 | ((brain* or cerebr* or cerebell* or vertebrobasilar or hemispher* or intracran* or intracerebral or infratentorial or supratentorial or mca or anterior circulation or posterior circulation or basal ganglia) adj5 (isch?emi* or infarct* or thrombo* or emboli*)).tw. |
| 4 | or/1-3 |
| 5 | (acute or sudden or spontaneous).mp. |
| 6 | 4 and 5 |
| 7 | exp antihypertensive agents/ or exp vasodilator agents/ or exp adrenergic agonists/ or exp diuretics/ or exp thiazides/ or exp sodium chloride symporter inhibitors/ or exp sodium potassium chloride symporter inhibitors/ |
| 8 | exp angiotensin-converting enzyme inhibitors/ or exp angiotensin II type 1 receptor blockers/ or exp calcium channel blockers/ or exp adrenergic beta-antagonists/ or exp adrenergic alpha antagonists/ |
| 9 | exp enalapril/ or exp losartan/ or exp hydralazine/ |
| 10 | hypertension/ae, de, dt, pc or blood pressure/de, pd |
| 11 | (antihyperten* or anti-hypertens*).tw. |
| 12 | ((Blood pressure or hypertens*) adj5 (lower* or reduc* or decreas*)).tw. |
| 13 | (angiotensin adj3 convert* adj3 enzyme adj3 (inhibit* or antagonist? or block*)).tw. |
| 14 | (((ace or renin) adj3 inhibit*) or ACEI).tw. |
| 15 | (angiotensin adj3 receptor? adj3 (inhibit* or antagonist? or block*)).tw. |
| 16 | (calcium adj2 (inhibit* or antagonist? or block*)).tw. |
| 17 | (adrenergic adj3 beta* adj3 (inhibit* or antagonist? or block*)).tw. |
| 18 | (adrenergic adj3 alpha* adj3 (inhibit* or antagonist? or block*)).tw. |
| 19 | ((loop or ceiling) adj diuretic?).tw. |
| 20 | (amiloride or benzothiadiazine or bendroflumethiazide or bumetanide or chlorothiazide or cyclopenthiazide or furosemide or hydrochlorothiazide or hydroflumethiazide or methyclothiazide or metolazone or polythiazide or trichlormethiazide or veratide or thiazide?).mp. |
| 21 | (chlorthalidone or chlortalidone or phthalamudine or chlorphthalidolone or oxodoline or thalitone or hygroton or indapamide or metindamide or s-1520 or s1520 or se-1520 or se1520).mp. |
| 22 | (alacepril or altiopril or benazepril or captopril or ceronapril or cilazapril or delapril or enalapril or fosinopril or idapril or imidapril or lisinopril or moexipril or moveltipril or pentopril or perindopril or quinapril or ramipril or spirapril or temocapril or trandolapril or zofenopril or aliskiren or remikiren).mp. |
| 23 | (KT3-671 or candesartan or eprosartan or irbesartan or losartan or olmesartan or tasosartan or telmisartan or valsartan).mp. |
| 24 | (amlodipine or amrinone or bencyclane or bepridil or cinnarizine or conotoxins or diltiazem or felodipine or fendiline or flunarizine or gallopamil or isradipine or lidoflazine or magnesium sulfate or mibefradil or nicardipine or nifedipine or nimodipine or nisoldipine or nitrendipine or perhexiline or prenylamine or verapamil or omega-agatoxin iva or omega-conotoxin gvia or omega-conotoxins).mp. |
| 25 | (methyldopa or alphamethyldopa or amodopa or dopamet or dopegyt or dopegit or dopegite or emdopa or hyperpax or hyperpaxa or methylpropionic acid or dopergit or meldopa or methyldopate or medopa or medomet or sembrina or aldomet or aldometil or aldomin or hydopa or methyldihydroxyphenylalanine or methyl dopa or mulfasin or presinol or presolisin or sedometil or sembrina or taquinil or dihydroxyphenylalanine or methylphenylalanine or methylalanine or alpha methyl dopa).mp. |
| 26 | (reserpine or serpentina or rauwolfia or serpasil).mp. |
| 27 | (clonidine or adesipress or arkamin or caprysin or catapres* or catasan or chlofazolin or chlophazolin or clinidine or clofelin* or clofenil or clomidine or clondine or clonistada or clonnirit or clophelin* or dichlorophenylaminoimidazoline or dixarit or duraclon or gemiton or haemiton or hemiton or imidazoline or isoglaucon or klofelin or klofenil or m-5041t or normopresan or paracefan or st-155 or st 155 or tesno timelets).mp. |
| 28 | (hydralazin* or hydrallazin* or hydralizine or hydrazinophtalazine or hydrazinophthalazine or hydrazinophtalizine or dralzine or hydralacin or hydrolazine or hypophthalin or hypoftalin or hydrazinophthalazine or idralazina or 1-hydrazinophthalazine or apressin or nepresol or apressoline or apresoline or apresolin or alphapress or alazine or idralazina or lopress or plethorit or praeparat).mp. |
| 29 | (acebutolol or adimolol or afurolol or alprenolol or amosulalol or arotinolol or atenolol or befunolol or betaxolol or bevantolol or bisoprolol or bopindolol or bornaprolol or brefonalol or bucindolol or bucumolol or bufetolol or bufuralol or bunitrolol or bunolol or bupranolol or butofilolol or butoxamine or carazolol or carteolol or carvedilol or celiprolol or cetamolol or chlortalidone cloranolol or cyanoiodopindolol or cyanopindolol or deacetylmetipranolol or diacetolol or dihydroalprenolol or dilevalol or epanolol or esmolol or exaprolol or falintolol or flestolol or flusoxolol or hydroxybenzylpinodolol or hydroxycarteolol or hydroxymetoprolol or indenolol or iodocyanopindolol or iodopindolol or iprocrolol or isoxaprolol or labetalol or landiolol or levobunolol or levomoprolol or medroxalol or mepindolol or methylthiopropranolol or metipranolol or metoprolol or moprolol or nadolol or oxprenolol or penbutolol or pindolol or nadolol or nebivolol or nifenalol or nipradilol or oxprenolol or pafenolol or pamatolol or penbutolol or pindolol or practolol or primidolol or prizidilol or procinolol or pronetalol or propranolol or proxodolol or ridazolol or salcardolol or soquinolol or sotalol or spirendolol or talinolol or tertatolol or tienoxolol or tilisolol or timolol or tolamolol or toliprolol or tribendilol or xibenolol).mp. |
| 30 | (alfuzosin or bunazosin or doxazosin or metazosin or neldazosin or prazosin or silodosin or tamsulosin or terazosin or tiodazosin or trimazosin).mp. |
| 31 | or/7-30 |
| 32 | 6 and 31 |
| 33 | thrombolytic therapy/ |
| 34 | fibrinolytic agents/ or fibrinolysin/ or plasminogen/ or tissue plasminogen activator/ or exp plasminogen activators/ or urokinase-type plasminogen activator/ |
| 35 | fibrinolysis/ |
| 36 | (thromboly? or fibrinoly? or recanalis? or recanaliz?).tw. |
| 37 | ((clot? or thrombus) adj5 (lyse or lysis or dissolve? or dissolution)).tw. |
| 38 | (tPA or t-PA or rtPA or rt-PA or plasminogen or plasmin or alteplase or actilyse).tw. |
| 39 | (anistreplase or streptodornase or streptokinase or urokinase or pro?urokinase or rpro?uk or lumbrokinase or duteplase or lanoteplase or pamiteplase or reteplase or saruplase or staphylokinase or streptase or tenecteplase).tw. |
| 40 | or/33-39 |
| 41 | (endovascular adj (therap* or treatment? or procedure? or method? or stent* or adverse effect? thromb* or embol*)).tw. |
| 42 | ((intra-arterial or intraarterial or mechanical or rheolytic) adj (thromb* or embol*)).tw. |
| 43 | (angiojet or stent retriever or trevo retriever or penumbra device or solitaire revasculari?ation or balloon angioplasty).tw. |
| 44 | or/41-43 |
| 45 | 40 or 44 |
| 46 | 32 and 45 |
| 47 | exp randomized controlled trial/ |
| 48 | controlled clinical trial/ |
| 49 | random*.ti,ab. |
| 50 | randomization/ |
| 51 | intermethod comparison/ |
| 52 | placebo.ti,ab. |
| 53 | (compare or compared or comparison).ti,ab. |
| 54 | ((evaluated or evaluate or evaluating or assessed or assess) and (compare or compared or comparing or comparison)).ab. |
| 55 | (open adj label).ti,ab. |
| 56 | ((double or single or doubly or singly) adj (blind or blinded or blindly)).ti,ab. |
| 57 | double blind procedure/ |
| 58 | parallel group*1.ti,ab. |
| 59 | (crossover or cross over).ti,ab. |
| 60 | ((assign* or match or matched or allocation) adj5 (alternate or group*1 or intervention*1 or patient*1 or subject*1 or participant*1)).ti,ab. |
| 61 | (assigned or allocated).ti,ab. |
| 62 | (controlled adj7 (study or design or trial)).ti,ab. |
| 63 | (volunteer or volunteers).ti,ab. |
| 64 | human experiment/ |
| 65 | trial.ti. |
| 66 | or/47-65 |
| 67 | (random* adj sampl* adj7 ("cross section*" or questionnaire*1 or survey* or database*1)).ti,ab. not (comparative study/ or controlled study/ or randomi?ed controlled.ti,ab. or randomly assigned.ti,ab.) |
| 68 | cross-sectional study/ not (exp randomized controlled trial/ or controlled clinical trial/ or controlled study/ or randomi?ed controlled.ti,ab. or control group*1.ti,ab.) |
| 69 | (((case adj control*) and random*) not randomi?ed controlled).ti,ab. |
| 70 | systematic review.ti,ab. not (trial or study).ti. |
| 71 | (non random* not random*).ti,ab. |
| 72 | "random field*".ti,ab. |
| 73 | (random cluster adj3 sample*).ti,ab. |
| 74 | (review.ab. and review.pt.) not trial.ti. |
| 75 | "we searched".ab. and (review.ti. or review.pt.) |
| 76 | "update review".ab. |
| 77 | (databases adj4 searched).ab. |
| 78 | (rat or rats or mouse or mice or swine or porcine or murine or sheep or lambs or pigs or piglets or rabbit or rabbits or cat or cats or dog or dogs or cattle or bovine or monkey or monkeys or trout or marmoset*1).ti. and animal experiment/ |
| 79 | animal experiment/ not (human experiment/ or human/) |
| 80 | (cardi* or myocard* or heart or coronary).tw. |
| 81 | exp child/ or adolescent/ or exp infant/ or exp Pediatrics/ or child*.mp. or p?ediat*.mp. or neonat*.mp. or newborn*.mp. or infant*.mp. or baby*.mp. or babies.mp. or toddler*.mp. or minors*.mp. or adolesc*.mp. or preteen*.mp. or teen*.mp. or juvenil*.mp. or youth*.mp. or preschool*.mp. or school*.mp. or kindergarten*.mp. or kid.mp. or kids.mp. |
| 82 | or/67-81 |
| 83 | 66 not 82 |
| 84 | 46 and 83 |

Cochrane Library

| # | Query |
| --- | --- |
| 1 | [mh "cerebrovascular disorders"] or [mh "basal ganglia cerebrovascular disease"] or [mh "brain ischemia"] or [mh "carotid artery diseases"] or [mh "carotid artery thrombosis"] or [mh "carotid artery, internal, dissection"] or [mh "stroke, lacunar"] or [mh "intracranial arterial diseases"] or [mh "cerebral arterial diseases"] or [mh "infarction, anterior cerebral artery"] or [mh "infarction, middle cerebral artery"] or [mh "infarction, posterior cerebral artery"] or [mh "intracranial embolism and thrombosis"] or [mh stroke] or [mh "brain infarction"] or [mh "vertebral artery dissection"] |
| 2 | ((brain or cerebr* or cerebell* or vertebrobasil* or hemispher* or intracran* or intracerebral or infratentorial or supratentorial or middle cerebr* or mca* or anterior circulation) near/5 (isch*emi* or infarct* or thrombo* or emboli* or occlus* or hypoxi*)):ti,ab,kw |
| 3 | (isch*emi* near/6 (stroke* or apoplex* or cerebral vasc* or cerebrovasc* or cva or attack*)):ti,ab,kw |
| 4 | {or #1-#3} |
| 5 | [mh "antihypertensive agents"] or [mh "vasodilator agents"] or [mh "adrenergic agonists"] or [mh "diuretics"] or [mh "thiazides"] or [mh "sodium chloride symporter inhibitors"] or [mh "sodium potassium chloride symporter inhibitors"] or [mh "angiotensin-converting enzyme inhibitors"] or [mh "angiotensin ii type 1 receptor blockers"] or [mh "calcium channel blockers"] or [mh "adrenergic beta-antagonists"] or [mh "adrenergic alpha-antagonists"] or [mh "enalapril"] or [mh "losartan"] or [mh "hydralazine"] |
| 6 | MeSH descriptor: [Hypertension] this term only and with qualifier(s): [drug therapy - DT, prevention & control - PC] |
| 7 | MeSH descriptor: [Blood Pressure] this term only and with qualifier(s): [drug effects - DE] |
| 8 | ((antihypertens* or anti-hypertens*)):ti,ab,kw |
| 9 | ((("blood pressure" or hypertens*) near/5 (lower* or reduc* or decreas*))):ti,ab,kw |
| 10 | ((angiotensin near/3 convert* near/3 enzyme near/3 (inhibit* or antagonist* or block*))):ti,ab,kw |
| 11 | ((((ace or renin) near/3 inhibit*) or ACEI)):ti,ab,kw |
| 12 | ((angiotensin near/3 receptor* near/3 (inhibit* or antagonist* or block*))):ti,ab,kw |
| 13 | ((calcium near/2 (inhibit* or antagonist* or block*))):ti,ab,kw |
| 14 | ((adrenergic near/3 beta* near/3 (inhibit* or antagonist* or block*))):ti,ab,kw |
| 15 | ((adrenergic near/3 alpha* near/3 (inhibit* or antagonist* or block*))):ti,ab,kw |
| 16 | (((loop or ceiling) next diuretic*)):ti,ab,kw |
| 17 | ((amiloride or benzothiadiazine or bendroflumethiazide or bumetanide or chlorothiazide or cyclopenthiazide or furosemide or hydrochlorothiazide or hydroflumethiazide or methyclothiazide or metolazone or polythiazide or trichlormethiazide or veratide or thiazide*)):ti,ab,kw |
| 18 | ((chlorthalidone or chlortalidone or phthalamudine or chlorphthalidolone or oxodoline or thalitone or hygroton or indapamide or metindamide or "s-1520" or s1520 or "se-1520" or se1520)):ti,ab,kw |
| 19 | ((alacepril or altiopril or benazepril or captopril or ceronapril or cilazapril or delapril or enalapril or fosinopril or idapril or imidapril or lisinopril or moexipril or moveltipril or pentopril or perindopril or quinapril or ramipril or spirapril or temocapril or trandolapril or zofenopril or aliskiren or remikiren)):ti,ab,kw |
| 20 | (("KT3-671" or candesartan or eprosartan or irbesartan or losartan or olmesartan or tasosartan or telmisartan or valsartan)):ti,ab,kw |
| 21 | ((amlodipine or amrinone or bencyclane or bepridil or cinnarizine or conotoxins or diltiazem or felodipine or fendiline or flunarizine or gallopamil or isradipine or lidoflazine or "magnesium sulphate" or mibefradil or nicardipine or nifedipine or nimodipine or nisoldipine or nitrendipine or perhexiline or prenylamine or verapamil or "omega-agatoxin iva" or "omega-conotoxin gvia" or "omega-conotoxins")):ti,ab,kw |
| 22 | ((methyldopa or alphamethyldopa or amodopa or dopamet or dopegyt or dopegit or dopegite or emdopa or hyperpax or hyperpaxa or "methylpropionic acid" or dopergit or meldopa or methyldopate or medopa or medomet or sembrina or aldomet or aldometil or aldomin or hydopa or methyldihydroxyphenylalanine or "methyl dopa" or mulfasin or presinol or presolisin or sedometil or sembrina or taquinil or dihydroxyphenylalanine or methylphenylalanine or methylalanine or "alpha methyl dopa")):ti,ab,kw |
| 23 | ((reserpine or serpentina or rauwolfia or serpasil)):ti,ab,kw |
| 24 | ((clonidine or adesipress or arkamin or caprysin or catapres* or catasan or chlofazolin or chlophazolin or clinidine or clofelin* or clofenil or clomidine or clondine or clonistada or clonnirit or clophelin* or dichlorophenylaminoimidazoline or dixarit or duraclon or gemiton or haemiton or hemiton or imidazoline or isoglaucon or klofelin or klofenil or "m-5041t" or normopresan or paracefan or "st-155" or "st 155" or "tesno timelets")):ti,ab,kw |
| 25 | ((hydralazin* or hydrallazin* or hydralizine or hydrazinophtalazine or hydrazinophthalazine or hydrazinophtalizine or dralzine or hydralacin or hydrolazine or hypophthalin or hypoftalin or hydrazinophthalazine or idralazina or "1-hydrazinophthalazine" or apressin or nepresol or apressoline or apresoline or apresolin or alphapress or alazine or idralazina or lopress or plethorit or praeparat)):ti,ab,kw |
| 26 | ((acebutolol or adimolol or afurolol or alprenolol or amosulalol or arotinolol or atenolol or befunolol or betaxolol or bevantolol or bisoprolol or bopindolol or bornaprolol or brefonalol or bucindolol or bucumolol or bufetolol or bufuralol or bunitrolol or bunolol or bupranolol or butofilolol or butoxamine or carazolol or carteolol or carvedilol or celiprolol or cetamolol or chlortalidone cloranolol or cyanoiodopindolol or cyanopindolol or deacetylmetipranolol or diacetolol or dihydroalprenolol or dilevalol or epanolol or esmolol or exaprolol or falintolol or flestolol or flusoxolol or hydroxybenzylpinodolol or hydroxycarteolol or hydroxymetoprolol or indenolol or iodocyanopindolol or iodopindolol or iprocrolol or isoxaprolol or labetalol or landiolol or levobunolol or levomoprolol or medroxalol or mepindolol or methylthiopropranolol or metipranolol or metoprolol or moprolol or nadolol or oxprenolol or penbutolol or pindolol or nadolol or nebivolol or nifenalol or nipradilol or oxprenolol or pafenolol or pamatolol or penbutolol or pindolol or practolol or primidolol or prizidilol or procinolol or pronetalol or propranolol or proxodolol or ridazolol or salcardolol or soquinolol or sotalol or spirendolol or talinolol or tertatolol or tienoxolol or tilisolol or timolol or tolamolol or toliprolol or tribendilol or xibenolol)):ti,ab,kw |
| 27 | ((alfuzosin or bunazosin or doxazosin or metazosin or neldazosin or prazosin or silodosin or tamsulosin or terazosin or tiodazosin or trimazosin)):ti,ab,kw |
| 28 | {or #5-#27} |
| 29 | #4 and #28 |
| 30 | [mh "thrombolytic therapy"] or [mh "fibrinolytic agents"] or [mh "fibrinolysin"] or [mh "plasminogen"] or [mh "tissue plasminogen activator"] or [mh "plasminogen activators"] or [mh "urokinase-type plasminogen activator"] or [mh "fibrinolysis"] |
| 31 | (thromboly* or fibrinoly* or recanalis* or recanaliz*):ti,ab,kw |
| 32 | (((clot* or thrombus) near/5 (lyse or lysis or dissolve* or dissolution))):ti,ab,kw |
| 33 | (tPA or t-PA or rtPA or rt-PA or plasminogen or plasmin or alteplase or actilyse):ti,ab,kw |
| 34 | (anistreplase or streptodornase or streptokinase or urokinase or pro?urokinase or rpro?uk or lumbrokinase or duteplase or lanoteplase or pamiteplase or reteplase or saruplase or staphylokinase or streptase):ti,ab,kw |
| 35 | {or #30-#34} |
| 36 | (endovascular adj (therap* or treatment? or procedure? or method? or stent* or adverse effect? thromb* or embol*)):ti,ab,kw |
| 37 | ((intra-arterial or intraarterial or mechanical or rheolytic) adj (thromb* or embol*)):ti,ab,kw |
| 38 | (angiojet or stent retriever or trevo retriever or penumbra device or solitaire revasculari?ation or balloon angioplasty):ti,ab,kw |
| 39 | {or #36-#38} |
| 40 | #35 or #39 |
| 41 | #29 and #40 |
| 42 | [mh "infant, newborn"] or [mh "pediatrics"] or [mh "adolescent"] |
| 43 | (cardi* or myocard* or heart or coronary):ti,ab,kw |
| 44 | (child* or p?ediat* or neonat* or newborn* or infant* or baby* or babies or toddler* or minors* or adolesc* or preteen* or teen* or juvenil* or youth* or preschool* or school* or kindergarten* or kid*):ti,ab,kw |
| 45 | {or #42-#44} |
| 46 | #41 not #45 |

# PICO 5. In patients with acute ischaemic stroke not treated with reperfusion therapies (intravenous thrombolysis or mechanical thrombectomy) and with clinical deterioration, does induced hypertension by any vasopressor drug compared to no drug improve outcome?

Medline (Ovid)

| # | Query |
| --- | --- |
| 1 | exp cerebrovascular disorders/ or exp basal ganglia cerebrovascular disease/ or exp brain ischemia/ or exp carotid artery diseases/ or exp intracranial arterial diseases/ or exp intracranial embolism/ or exp intracranial thrombosis/ or exp stroke/ or exp brain infarction/ or exp transient ischemic attack/ |
| 2 | (stroke or cerebrovasc* or brain vasc* or cerebral vasc* or cva* or apoplex* or large vessel occlusion or transient isch?emic attack or hemiparesis or hemiplegia or intracerebral arteriosclerosis).tw. |
| 3 | ((brain* or cerebr* or cerebell* or vertebrobasilar or hemispher* or intracran* or intracerebral or infratentorial or supratentorial or mca or anterior circulation or posterior circulation or basal ganglia) adj5 (isch?emi* or infarct* or thrombo* or emboli*)).tw. |
| 4 | or/1-3 |
| 5 | exp vasoconstriction/ or exp vasoconstrictor agents/ |
| 6 | ((blood pressure*) adj5 (increas* or ris* or elevat*)).mp. |
| 7 | exp dopamine/ or exp dobutamine/ or exp norepinephrine/ or exp epinephrine/ or exp isoproterenol/ or exp phenylephrine/ |
| 8 | (dopamine or dobutamine or nor-adrenaline or noradrenaline or nor-epinephrine or norepinephrine or epinephrine or adrenaline or isoproterenol or isoprenaline or phenylephrine).mp. |
| 9 | or/5-8 |
| 10 | 4 and 9 |
| 11 | thrombolytic therapy/ |
| 12 | fibrinolytic agents/ or fibrinolysin/ or plasminogen/ or tissue plasminogen activator/ or exp plasminogen activators/ or urokinase-type plasminogen activator/ |
| 13 | fibrinolysis/ |
| 14 | (thromboly? or fibrinoly? or recanalis? or recanaliz?).mp. |
| 15 | ((clot? or thrombus) adj5 (lyse or lysis or dissolve? or dissolution)).mp. |
| 16 | (tPA or t-PA or rtPA or rt-PA or plasminogen or plasmin or alteplase or actilyse).mp. |
| 17 | (anistreplase or streptodornase or streptokinase or urokinase or pro?urokinase or rpro?uk or lumbrokinase or duteplase or lanoteplase or pamiteplase or reteplase or saruplase or staphylokinase or streptase or tenecteplase).mp. |
| 18 | or/11-17 |
| 19 | (endovascular adj (therap* or treatment? or procedure? or method? or stent* or adverse effect? thromb* or embol*)).mp. |
| 20 | ((intra-arterial or intraarterial or mechanical or rheolytic) adj (thromb* or embol*)).mp. |
| 21 | (angiojet or stent retriever or trevo retriever or penumbra device or solitaire revasculari?ation or balloon angioplasty).mp. |
| 22 | or/19-21 |
| 23 | 18 or 22 |
| 24 | 10 and 23 |
| 25 | exp randomized controlled trial/ |
| 26 | controlled clinical trial.pt. |
| 27 | randomized.ab. |
| 28 | placebo.ab. |
| 29 | drug therapy.fs. |
| 30 | randomly.ab. |
| 31 | trial.ab. |
| 32 | groups.ab. |
| 33 | or/25-32 |
| 34 | 24 and 33 |
| 35 | exp animals/ not humans.sh. |
| 36 | (cardi* or myocard* or heart or coronary).tw. |
| 37 | exp child/ or adolescent/ or exp infant/ or exp Pediatrics/ or child*.mp. or p?ediat*.mp. or neonat*.mp. or newborn*.mp. or infant*.mp. or baby*.mp. or babies.mp. or toddler*.mp. or minors*.mp. or adolesc*.mp. or preteen*.mp. or teen*.mp. or juvenil*.mp. or youth*.mp. or preschool*.mp. or school*.mp. or kindergarten*.mp. or kid.mp. or kids.mp. |
| 38 | or/35-37 |
| 39 | 34 not 38 |

Embase (Ovid)

| # | Query |
| --- | --- |
| 1 | exp cerebrovascular disorders/ or exp basal ganglia cerebrovascular disease/ or exp brain ischemia/ or exp carotid artery diseases/ or exp intracranial arterial diseases/ or exp intracranial embolism/ or exp intracranial thrombosis/ or exp stroke/ or exp brain infarction/ or exp transient ischemic attack/ |
| 2 | (stroke or cerebrovasc* or brain vasc* or cerebral vasc* or cva* or apoplex* or large vessel occlusion or transient isch?emic attack or hemiparesis or hemiplegia or intracerebral arteriosclerosis).tw. |
| 3 | ((brain* or cerebr* or cerebell* or vertebrobasilar or hemispher* or intracran* or intracerebral or infratentorial or supratentorial or mca or anterior circulation or posterior circulation or basal ganglia) adj5 (isch?emi* or infarct* or thrombo* or emboli*)).tw. |
| 4 | or/1-3 |
| 5 | (acute or sudden or spontaneous).mp. |
| 6 | 4 and 5 |
| 7 | exp vasoconstriction/ or exp vasoconstrictor agents/ |
| 8 | ((blood pressure*) adj5 (increas* or ris* or elevat*)).mp. |
| 9 | exp dopamine/ or exp dobutamine/ or exp norepinephrine/ or exp epinephrine/ or exp isoproterenol/ or exp phenylephrine/ |
| 10 | (dopamine or dobutamine or nor-adrenaline or noradrenaline or nor-epinephrine or norepinephrine or epinephrine or adrenaline or isoproterenol or isoprenaline or phenylephrine).mp. |
| 11 | or/7-10 |
| 12 | 6 and 11 |
| 13 | thrombolytic therapy/ |
| 14 | fibrinolytic agents/ or fibrinolysin/ or plasminogen/ or tissue plasminogen activator/ or exp plasminogen activators/ or urokinase-type plasminogen activator/ |
| 15 | fibrinolysis/ |
| 16 | (thromboly? or fibrinoly? or recanalis? or recanaliz?).mp. |
| 17 | ((clot? or thrombus) adj5 (lyse or lysis or dissolve? or dissolution)).mp. |
| 18 | (tPA or t-PA or rtPA or rt-PA or plasminogen or plasmin or alteplase or actilyse).mp. |
| 19 | (anistreplase or streptodornase or streptokinase or urokinase or pro?urokinase or rpro?uk or lumbrokinase or duteplase or lanoteplase or pamiteplase or reteplase or saruplase or staphylokinase or streptase or tenecteplase).mp. |
| 20 | or/13-19 |
| 21 | (endovascular adj (therap* or treatment? or procedure? or method? or stent* or adverse effect? thromb* or embol*)).mp. |
| 22 | ((intra-arterial or intraarterial or mechanical or rheolytic) adj (thromb* or embol*)).mp. |
| 23 | (angiojet or stent retriever or trevo retriever or penumbra device or solitaire revasculari?ation or balloon angioplasty).mp. |
| 24 | or/21-23 |
| 25 | 20 or 24 |
| 26 | 12 and 25 |
| 27 | exp randomized controlled trial/ |
| 28 | controlled clinical trial/ |
| 29 | random*.ti,ab. |
| 30 | randomization/ |
| 31 | intermethod comparison/ |
| 32 | placebo.ti,ab. |
| 33 | (compare or compared or comparison).ti,ab. |
| 34 | ((evaluated or evaluate or evaluating or assessed or assess) and (compare or compared or comparing or comparison)).ab. |
| 35 | (open adj label).ti,ab. |
| 36 | ((double or single or doubly or singly) adj (blind or blinded or blindly)).ti,ab. |
| 37 | double blind procedure/ |
| 38 | parallel group*1.ti,ab. |
| 39 | (crossover or cross over).ti,ab. |
| 40 | ((assign* or match or matched or allocation) adj5 (alternate or group*1 or intervention*1 or patient*1 or subject*1 or participant*1)).ti,ab. |
| 41 | (assigned or allocated).ti,ab. |
| 42 | (controlled adj7 (study or design or trial)).ti,ab. |
| 43 | (volunteer or volunteers).ti,ab. |
| 44 | human experiment/ |
| 45 | trial.ti. |
| 46 | or/27-45 |
| 47 | (random* adj sampl* adj7 ("cross section*" or questionnaire*1 or survey* or database*1)).ti,ab. not (comparative study/ or controlled study/ or randomi?ed controlled.ti,ab. or randomly assigned.ti,ab.) |
| 48 | cross-sectional study/ not (exp randomized controlled trial/ or controlled clinical trial/ or controlled study/ or randomi?ed controlled.ti,ab. or control group*1.ti,ab.) |
| 49 | (((case adj control*) and random*) not randomi?ed controlled).ti,ab. |
| 50 | systematic review.ti,ab. not (trial or study).ti. |
| 51 | (non random* not random*).ti,ab. |
| 52 | "random field*".ti,ab. |
| 53 | (random cluster adj3 sample*).ti,ab. |
| 54 | (review.ab. and review.pt.) not trial.ti. |
| 55 | "we searched".ab. and (review.ti. or review.pt.) |
| 56 | "update review".ab. |
| 57 | (databases adj4 searched).ab. |
| 58 | (rat or rats or mouse or mice or swine or porcine or murine or sheep or lambs or pigs or piglets or rabbit or rabbits or cat or cats or dog or dogs or cattle or bovine or monkey or monkeys or trout or marmoset*1).ti. and animal experiment/ |
| 59 | animal experiment/ not (human experiment/ or human/) |
| 60 | (cardi* or myocard* or heart or coronary).tw. |
| 61 | exp child/ or adolescent/ or exp infant/ or exp Pediatrics/ or child*.mp. or p?ediat*.mp. or neonat*.mp. or newborn*.mp. or infant*.mp. or baby*.mp. or babies.mp. or toddler*.mp. or minors*.mp. or adolesc*.mp. or preteen*.mp. or teen*.mp. or juvenil*.mp. or youth*.mp. or preschool*.mp. or school*.mp. or kindergarten*.mp. or kid.mp. or kids.mp. |
| 62 | or/47-61 |
| 63 | 46 not 62 |
| 64 | 26 and 63 |

Cochrane Library

| # | Query |
| --- | --- |
| 1 | [mh "cerebrovascular disorders"] or [mh "basal ganglia cerebrovascular disease"] or [mh "brain ischemia"] or [mh "carotid artery diseases"] or [mh "carotid artery thrombosis"] or [mh "carotid artery, internal, dissection"] or [mh "stroke, lacunar"] or [mh "intracranial arterial diseases"] or [mh "cerebral arterial diseases"] or [mh "infarction, anterior cerebral artery"] or [mh "infarction, middle cerebral artery"] or [mh "infarction, posterior cerebral artery"] or [mh "intracranial embolism and thrombosis"] or [mh stroke] or [mh "brain infarction"] or [mh "vertebral artery dissection"] |
| 2 | ((brain or cerebr* or cerebell* or vertebrobasil* or hemispher* or intracran* or intracerebral or infratentorial or supratentorial or middle cerebr* or mca* or anterior circulation) near/5 (isch*emi* or infarct* or thrombo* or emboli* or occlus* or hypoxi*)):ti,ab,kw |
| 3 | (isch*emi* near/6 (stroke* or apoplex* or cerebral vasc* or cerebrovasc* or cva or attack*)):ti,ab,kw |
| 4 | {or #1-#3} |
| 5 | [mh "vasoconstriction"] or [mh "vasoconstrictor agents"] |
| 6 | ((blood pressure*) near/5 (increas* or ris* or elevat*)):ti,ab,kw |
| 7 | [mh "dopamine"] or [mh "dobutamine"] or [mh "norepinephrine"] or [mh "epinephrine"] or [mh "isoproterenol"] or [mh "phenylephrine"] |
| 8 | (dopamine or dobutamine or nor-adrenaline or noradrenaline or nor-epinephrine or norepinephrine or epinephrine or adrenaline or isoproterenol or isoprenaline or phenylephrine):ti,ab,kw |
| 9 | {or #5-#8} |
| 10 | #4 and #9 |
| 11 | [mh "thrombolytic therapy"] or [mh "fibrinolytic agents"] or [mh "fibrinolysin"] or [mh "plasminogen"] or [mh "tissue plasminogen activator"] or [mh "plasminogen activators"] or [mh "urokinase-type plasminogen activator"] or [mh "fibrinolysis"] |
| 12 | (thromboly* or fibrinoly* or recanalis* or recanaliz*):ti,ab,kw |
| 13 | (((clot* or thrombus) near/5 (lyse or lysis or dissolve* or dissolution))):ti,ab,kw |
| 14 | (tPA or t-PA or rtPA or rt-PA or plasminogen or plasmin or alteplase or actilyse):ti,ab,kw |
| 15 | (anistreplase or streptodornase or streptokinase or urokinase or pro?urokinase or rpro?uk or lumbrokinase or duteplase or lanoteplase or pamiteplase or reteplase or saruplase or staphylokinase or streptase):ti,ab,kw |
| 16 | {or #11-#15} |
| 17 | (endovascular adj (therap* or treatment? or procedure? or method? or stent* or adverse effect? thromb* or embol*)):ti,ab,kw |
| 18 | ((intra-arterial or intraarterial or mechanical or rheolytic) adj (thromb* or embol*)):ti,ab,kw |
| 19 | (angiojet or stent retriever or trevo retriever or penumbra device or solitaire revasculari?ation or balloon angioplasty):ti,ab,kw |
| 20 | {or #17-#19} |
| 21 | #16 or #20 |
| 22 | #10 and #21 |
| 23 | [mh "infant, newborn"] or [mh "pediatrics"] or [mh "adolescent"] |
| 24 | (cardi* or myocard* or heart or coronary):ti,ab,kw |
| 25 | (child* or p?ediat* or neonat* or newborn* or infant* or baby* or babies or toddler* or minors* or adolesc* or preteen* or teen* or juvenil* or youth* or preschool* or school* or kindergarten* or kid*):ti,ab,kw |
| 26 | {or #23-#25} |
| 27 | #22 not #26 |

# PICO 6. In patients with acute ischaemic stroke, does continuing versus temporarily stopping previous oral blood pressure lowering therapy improve outcome?

Medline (Ovid)

| # | Query |
| --- | --- |
| 1 | exp cerebrovascular disorders/ or exp basal ganglia cerebrovascular disease/ or exp brain ischemia/ or exp carotid artery diseases/ or exp intracranial arterial diseases/ or exp intracranial embolism/ or exp intracranial thrombosis/ or exp stroke/ or exp brain infarction/ or exp transient ischemic attack/ |
| 2 | (stroke or cerebrovasc* or brain vasc* or cerebral vasc* or cva* or apoplex* or large vessel occlusion or transient isch?emic attack or hemiparesis or hemiplegia or intracerebral arteriosclerosis).tw. |
| 3 | ((brain* or cerebr* or cerebell* or vertebrobasilar or hemispher* or intracran* or intracerebral or infratentorial or supratentorial or mca or anterior circulation or posterior circulation or basal ganglia) adj5 (isch?emi* or infarct* or thrombo* or emboli*)).tw. |
| 4 | or/1-3 |
| 5 | exp antihypertensive agents/ or exp vasodilator agents/ or exp adrenergic agonists/ or exp diuretics/ or exp thiazides/ or exp sodium chloride symporter inhibitors/ or exp sodium potassium chloride symporter inhibitors/ |
| 6 | exp angiotensin-converting enzyme inhibitors/ or exp angiotensin II type 1 receptor blockers/ or exp calcium channel blockers/ or exp adrenergic beta-antagonists/ or exp adrenergic alpha antagonists/ |
| 7 | exp enalapril/ or exp losartan/ or exp hydralazine/ |
| 8 | exp hypertension/ or exp blood pressure/ |
| 9 | (antihyperten* or anti-hypertens*).tw. |
| 10 | ((Blood pressure or hypertens*) adj5 (lower* or reduc* or decreas*)).tw. |
| 11 | (angiotensin adj3 convert* adj3 enzyme adj3 (inhibit* or antagonist? or block*)).tw. |
| 12 | (((ace or renin) adj3 inhibit*) or ACEI).tw. |
| 13 | (angiotensin adj3 receptor? adj3 (inhibit* or antagonist? or block*)).tw. |
| 14 | (calcium adj2 (inhibit* or antagonist? or block*)).tw. |
| 15 | (adrenergic adj3 beta* adj3 (inhibit* or antagonist? or block*)).tw. |
| 16 | (adrenergic adj3 alpha* adj3 (inhibit* or antagonist? or block*)).tw. |
| 17 | ((loop or ceiling) adj diuretic?).tw. |
| 18 | (amiloride or benzothiadiazine or bendroflumethiazide or bumetanide or chlorothiazide or cyclopenthiazide or furosemide or hydrochlorothiazide or hydroflumethiazide or methyclothiazide or metolazone or polythiazide or trichlormethiazide or veratide or thiazide?).mp. |
| 19 | (chlorthalidone or chlortalidone or phthalamudine or chlorphthalidolone or oxodoline or thalitone or hygroton or indapamide or metindamide or s-1520 or s1520 or se-1520 or se1520).mp. |
| 20 | (alacepril or altiopril or benazepril or captopril or ceronapril or cilazapril or delapril or enalapril or fosinopril or idapril or imidapril or lisinopril or moexipril or moveltipril or pentopril or perindopril or quinapril or ramipril or spirapril or temocapril or trandolapril or zofenopril or aliskiren or remikiren).mp. |
| 21 | (KT3-671 or candesartan or eprosartan or irbesartan or losartan or olmesartan or tasosartan or telmisartan or valsartan).mp. |
| 22 | (amlodipine or amrinone or bencyclane or bepridil or cinnarizine or conotoxins or diltiazem or felodipine or fendiline or flunarizine or gallopamil or isradipine or lidoflazine or magnesium sulfate or mibefradil or nicardipine or nifedipine or nimodipine or nisoldipine or nitrendipine or perhexiline or prenylamine or verapamil or omega-agatoxin iva or omega-conotoxin gvia or omega-conotoxins).mp. |
| 23 | (methyldopa or alphamethyldopa or amodopa or dopamet or dopegyt or dopegit or dopegite or emdopa or hyperpax or hyperpaxa or methylpropionic acid or dopergit or meldopa or methyldopate or medopa or medomet or sembrina or aldomet or aldometil or aldomin or hydopa or methyldihydroxyphenylalanine or methyl dopa or mulfasin or presinol or presolisin or sedometil or sembrina or taquinil or dihydroxyphenylalanine or methylphenylalanine or methylalanine or alpha methyl dopa).mp. |
| 24 | (reserpine or serpentina or rauwolfia or serpasil).mp. |
| 25 | (clonidine or adesipress or arkamin or caprysin or catapres* or catasan or chlofazolin or chlophazolin or clinidine or clofelin* or clofenil or clomidine or clondine or clonistada or clonnirit or clophelin* or dichlorophenylaminoimidazoline or dixarit or duraclon or gemiton or haemiton or hemiton or imidazoline or isoglaucon or klofelin or klofenil or m-5041t or normopresan or paracefan or st-155 or st 155 or tesno timelets).mp. |
| 26 | (hydralazin* or hydrallazin* or hydralizine or hydrazinophtalazine or hydrazinophthalazine or hydrazinophtalizine or dralzine or hydralacin or hydrolazine or hypophthalin or hypoftalin or hydrazinophthalazine or idralazina or 1-hydrazinophthalazine or apressin or nepresol or apressoline or apresoline or apresolin or alphapress or alazine or idralazina or lopress or plethorit or praeparat).mp. |
| 27 | (acebutolol or adimolol or afurolol or alprenolol or amosulalol or arotinolol or atenolol or befunolol or betaxolol or bevantolol or bisoprolol or bopindolol or bornaprolol or brefonalol or bucindolol or bucumolol or bufetolol or bufuralol or bunitrolol or bunolol or bupranolol or butofilolol or butoxamine or carazolol or carteolol or carvedilol or celiprolol or cetamolol or chlortalidone cloranolol or cyanoiodopindolol or cyanopindolol or deacetylmetipranolol or diacetolol or dihydroalprenolol or dilevalol or epanolol or esmolol or exaprolol or falintolol or flestolol or flusoxolol or hydroxybenzylpinodolol or hydroxycarteolol or hydroxymetoprolol or indenolol or iodocyanopindolol or iodopindolol or iprocrolol or isoxaprolol or labetalol or landiolol or levobunolol or levomoprolol or medroxalol or mepindolol or methylthiopropranolol or metipranolol or metoprolol or moprolol or nadolol or oxprenolol or penbutolol or pindolol or nadolol or nebivolol or nifenalol or nipradilol or oxprenolol or pafenolol or pamatolol or penbutolol or pindolol or practolol or primidolol or prizidilol or procinolol or pronetalol or propranolol or proxodolol or ridazolol or salcardolol or soquinolol or sotalol or spirendolol or talinolol or tertatolol or tienoxolol or tilisolol or timolol or tolamolol or toliprolol or tribendilol or xibenolol).mp. |
| 28 | (alfuzosin or bunazosin or doxazosin or metazosin or neldazosin or prazosin or silodosin or tamsulosin or terazosin or tiodazosin or trimazosin).mp. |
| 29 | or/5-28 |
| 30 | (continu* or discontinu* or temporar*).ti,ab. |
| 31 | 4 and 29 and 30 |
| 32 | exp randomized controlled trial/ |
| 33 | controlled clinical trial.pt. |
| 34 | randomized.ab. |
| 35 | placebo.ab. |
| 36 | drug therapy.fs. |
| 37 | randomly.ab. |
| 38 | trial.ab. |
| 39 | groups.ab. |
| 40 | or/32-39 |
| 41 | 31 and 40 |
| 42 | exp animals/ not humans.sh. |
| 43 | (cardi* or myocard* or heart or coronary).tw. |
| 44 | exp child/ or adolescent/ or exp infant/ or exp Pediatrics/ or child*.mp. or p?ediat*.mp. or neonat*.mp. or newborn*.mp. or infant*.mp. or baby*.mp. or babies.mp. or toddler*.mp. or minors*.mp. or adolesc*.mp. or preteen*.mp. or teen*.mp. or juvenil*.mp. or youth*.mp. or preschool*.mp. or school*.mp. or kindergarten*.mp. or kid.mp. or kids.mp. |
| 45 | or/42-44 |
| 46 | 41 not 45 |

Embase (Ovid)

| # | Query |
| --- | --- |
| 1 | exp cerebrovascular disorders/ or exp basal ganglia cerebrovascular disease/ or exp brain ischemia/ or exp carotid artery diseases/ or exp intracranial arterial diseases/ or exp intracranial embolism/ or exp intracranial thrombosis/ or exp stroke/ or exp brain infarction/ or exp transient ischemic attack/ |
| 2 | (stroke or cerebrovasc* or brain vasc* or cerebral vasc* or cva* or apoplex* or large vessel occlusion or transient isch?emic attack or hemiparesis or hemiplegia or intracerebral arteriosclerosis).tw. |
| 3 | ((brain* or cerebr* or cerebell* or vertebrobasilar or hemispher* or intracran* or intracerebral or infratentorial or supratentorial or mca or anterior circulation or posterior circulation or basal ganglia) adj5 (isch?emi* or infarct* or thrombo* or emboli*)).tw. |
| 4 | or/1-3 |
| 5 | (acute or sudden or spontaneous).mp. |
| 6 | 4 and 5 |
| 7 | exp antihypertensive agents/ or exp vasodilator agents/ or exp adrenergic agonists/ or exp diuretics/ or exp thiazides/ or exp sodium chloride symporter inhibitors/ or exp sodium potassium chloride symporter inhibitors/ |
| 8 | exp angiotensin-converting enzyme inhibitors/ or exp angiotensin II type 1 receptor blockers/ or exp calcium channel blockers/ or exp adrenergic beta-antagonists/ or exp adrenergic alpha antagonists/ |
| 9 | exp enalapril/ or exp losartan/ or exp hydralazine/ |
| 10 | hypertension/ae, de, dt, pc or blood pressure/de, pd |
| 11 | (antihyperten* or anti-hypertens*).tw. |
| 12 | ((Blood pressure or hypertens*) adj5 (lower* or reduc* or decreas*)).tw. |
| 13 | (angiotensin adj3 convert* adj3 enzyme adj3 (inhibit* or antagonist? or block*)).tw. |
| 14 | (((ace or renin) adj3 inhibit*) or ACEI).tw. |
| 15 | (angiotensin adj3 receptor? adj3 (inhibit* or antagonist? or block*)).tw. |
| 16 | (calcium adj2 (inhibit* or antagonist? or block*)).tw. |
| 17 | (adrenergic adj3 beta* adj3 (inhibit* or antagonist? or block*)).tw. |
| 18 | (adrenergic adj3 alpha* adj3 (inhibit* or antagonist? or block*)).tw. |
| 19 | ((loop or ceiling) adj diuretic?).tw. |
| 20 | (amiloride or benzothiadiazine or bendroflumethiazide or bumetanide or chlorothiazide or cyclopenthiazide or furosemide or hydrochlorothiazide or hydroflumethiazide or methyclothiazide or metolazone or polythiazide or trichlormethiazide or veratide or thiazide?).mp. |
| 21 | (chlorthalidone or chlortalidone or phthalamudine or chlorphthalidolone or oxodoline or thalitone or hygroton or indapamide or metindamide or s-1520 or s1520 or se-1520 or se1520).mp. |
| 22 | (alacepril or altiopril or benazepril or captopril or ceronapril or cilazapril or delapril or enalapril or fosinopril or idapril or imidapril or lisinopril or moexipril or moveltipril or pentopril or perindopril or quinapril or ramipril or spirapril or temocapril or trandolapril or zofenopril or aliskiren or remikiren).mp. |
| 23 | (KT3-671 or candesartan or eprosartan or irbesartan or losartan or olmesartan or tasosartan or telmisartan or valsartan).mp. |
| 24 | (amlodipine or amrinone or bencyclane or bepridil or cinnarizine or conotoxins or diltiazem or felodipine or fendiline or flunarizine or gallopamil or isradipine or lidoflazine or magnesium sulfate or mibefradil or nicardipine or nifedipine or nimodipine or nisoldipine or nitrendipine or perhexiline or prenylamine or verapamil or omega-agatoxin iva or omega-conotoxin gvia or omega-conotoxins).mp. |
| 25 | (methyldopa or alphamethyldopa or amodopa or dopamet or dopegyt or dopegit or dopegite or emdopa or hyperpax or hyperpaxa or methylpropionic acid or dopergit or meldopa or methyldopate or medopa or medomet or sembrina or aldomet or aldometil or aldomin or hydopa or methyldihydroxyphenylalanine or methyl dopa or mulfasin or presinol or presolisin or sedometil or sembrina or taquinil or dihydroxyphenylalanine or methylphenylalanine or methylalanine or alpha methyl dopa).mp. |
| 26 | (reserpine or serpentina or rauwolfia or serpasil).mp. |
| 27 | (clonidine or adesipress or arkamin or caprysin or catapres* or catasan or chlofazolin or chlophazolin or clinidine or clofelin* or clofenil or clomidine or clondine or clonistada or clonnirit or clophelin* or dichlorophenylaminoimidazoline or dixarit or duraclon or gemiton or haemiton or hemiton or imidazoline or isoglaucon or klofelin or klofenil or m-5041t or normopresan or paracefan or st-155 or st 155 or tesno timelets).mp. |
| 28 | (hydralazin* or hydrallazin* or hydralizine or hydrazinophtalazine or hydrazinophthalazine or hydrazinophtalizine or dralzine or hydralacin or hydrolazine or hypophthalin or hypoftalin or hydrazinophthalazine or idralazina or 1-hydrazinophthalazine or apressin or nepresol or apressoline or apresoline or apresolin or alphapress or alazine or idralazina or lopress or plethorit or praeparat).mp. |
| 29 | (acebutolol or adimolol or afurolol or alprenolol or amosulalol or arotinolol or atenolol or befunolol or betaxolol or bevantolol or bisoprolol or bopindolol or bornaprolol or brefonalol or bucindolol or bucumolol or bufetolol or bufuralol or bunitrolol or bunolol or bupranolol or butofilolol or butoxamine or carazolol or carteolol or carvedilol or celiprolol or cetamolol or chlortalidone cloranolol or cyanoiodopindolol or cyanopindolol or deacetylmetipranolol or diacetolol or dihydroalprenolol or dilevalol or epanolol or esmolol or exaprolol or falintolol or flestolol or flusoxolol or hydroxybenzylpinodolol or hydroxycarteolol or hydroxymetoprolol or indenolol or iodocyanopindolol or iodopindolol or iprocrolol or isoxaprolol or labetalol or landiolol or levobunolol or levomoprolol or medroxalol or mepindolol or methylthiopropranolol or metipranolol or metoprolol or moprolol or nadolol or oxprenolol or penbutolol or pindolol or nadolol or nebivolol or nifenalol or nipradilol or oxprenolol or pafenolol or pamatolol or penbutolol or pindolol or practolol or primidolol or prizidilol or procinolol or pronetalol or propranolol or proxodolol or ridazolol or salcardolol or soquinolol or sotalol or spirendolol or talinolol or tertatolol or tienoxolol or tilisolol or timolol or tolamolol or toliprolol or tribendilol or xibenolol).mp. |
| 30 | (alfuzosin or bunazosin or doxazosin or metazosin or neldazosin or prazosin or silodosin or tamsulosin or terazosin or tiodazosin or trimazosin).mp. |
| 31 | or/7-30 |
| 32 | (continu* or discontinu* or temporar*).ti,ab. |
| 33 | 6 and 31 and 32 |
| 34 | exp randomized controlled trial/ |
| 35 | controlled clinical trial/ |
| 36 | random*.ti,ab. |
| 37 | randomization/ |
| 38 | intermethod comparison/ |
| 39 | placebo.ti,ab. |
| 40 | (compare or compared or comparison).ti,ab. |
| 41 | ((evaluated or evaluate or evaluating or assessed or assess) and (compare or compared or comparing or comparison)).ab. |
| 42 | (open adj label).ti,ab. |
| 43 | ((double or single or doubly or singly) adj (blind or blinded or blindly)).ti,ab. |
| 44 | double blind procedure/ |
| 45 | parallel group*1.ti,ab. |
| 46 | (crossover or cross over).ti,ab. |
| 47 | ((assign* or match or matched or allocation) adj5 (alternate or group*1 or intervention*1 or patient*1 or subject*1 or participant*1)).ti,ab. |
| 48 | (assigned or allocated).ti,ab. |
| 49 | (controlled adj7 (study or design or trial)).ti,ab. |
| 50 | (volunteer or volunteers).ti,ab. |
| 51 | human experiment/ |
| 52 | trial.ti. |
| 53 | or/34-52 |
| 54 | (random* adj sampl* adj7 ("cross section*" or questionnaire*1 or survey* or database*1)).ti,ab. not (comparative study/ or controlled study/ or randomi?ed controlled.ti,ab. or randomly assigned.ti,ab.) |
| 55 | cross-sectional study/ not (exp randomized controlled trial/ or controlled clinical trial/ or controlled study/ or randomi?ed controlled.ti,ab. or control group*1.ti,ab.) |
| 56 | (((case adj control*) and random*) not randomi?ed controlled).ti,ab. |
| 57 | systematic review.ti,ab. not (trial or study).ti. |
| 58 | (non random* not random*).ti,ab. |
| 59 | "random field*".ti,ab. |
| 60 | (random cluster adj3 sample*).ti,ab. |
| 61 | (review.ab. and review.pt.) not trial.ti. |
| 62 | "we searched".ab. and (review.ti. or review.pt.) |
| 63 | "update review".ab. |
| 64 | (databases adj4 searched).ab. |
| 65 | (rat or rats or mouse or mice or swine or porcine or murine or sheep or lambs or pigs or piglets or rabbit or rabbits or cat or cats or dog or dogs or cattle or bovine or monkey or monkeys or trout or marmoset*1).ti. and animal experiment/ |
| 66 | animal experiment/ not (human experiment/ or human/) |
| 67 | (cardi* or myocard* or heart or coronary).tw. |
| 68 | exp child/ or adolescent/ or exp infant/ or exp Pediatrics/ or child*.mp. or p?ediat*.mp. or neonat*.mp. or newborn*.mp. or infant*.mp. or baby*.mp. or babies.mp. or toddler*.mp. or minors*.mp. or adolesc*.mp. or preteen*.mp. or teen*.mp. or juvenil*.mp. or youth*.mp. or preschool*.mp. or school*.mp. or kindergarten*.mp. or kid.mp. or kids.mp. |
| 69 | or/54-68 |
| 70 | 53 not 69 |
| 71 | 33 and 70 |

Cochrane Library

| # | Query |
| --- | --- |
| 1 | [mh "cerebrovascular disorders"] or [mh "basal ganglia cerebrovascular disease"] or [mh "brain ischemia"] or [mh "carotid artery diseases"] or [mh "carotid artery thrombosis"] or [mh "carotid artery, internal, dissection"] or [mh "stroke, lacunar"] or [mh "intracranial arterial diseases"] or [mh "cerebral arterial diseases"] or [mh "infarction, anterior cerebral artery"] or [mh "infarction, middle cerebral artery"] or [mh "infarction, posterior cerebral artery"] or [mh "intracranial embolism and thrombosis"] or [mh stroke] or [mh "brain infarction"] or [mh "vertebral artery dissection"] |
| 2 | ((brain or cerebr* or cerebell* or vertebrobasil* or hemispher* or intracran* or intracerebral or infratentorial or supratentorial or middle cerebr* or mca* or anterior circulation) near/5 (isch*emi* or infarct* or thrombo* or emboli* or occlus* or hypoxi*)):ti,ab,kw |
| 3 | (isch*emi* near/6 (stroke* or apoplex* or cerebral vasc* or cerebrovasc* or cva or attack*)):ti,ab,kw |
| 4 | {or #1-#3} |
| 5 | [mh "antihypertensive agents"] or [mh "vasodilator agents"] or [mh "adrenergic agonists"] or [mh "diuretics"] or [mh "thiazides"] or [mh "sodium chloride symporter inhibitors"] or [mh "sodium potassium chloride symporter inhibitors"] or [mh "angiotensin-converting enzyme inhibitors"] or [mh "angiotensin ii type 1 receptor blockers"] or [mh "calcium channel blockers"] or [mh "adrenergic beta-antagonists"] or [mh "adrenergic alpha-antagonists"] or [mh "enalapril"] or [mh "losartan"] or [mh "hydralazine"] |
| 6 | MeSH descriptor: [Hypertension] this term only and with qualifier(s): [drug therapy - DT, prevention & control - PC] |
| 7 | MeSH descriptor: [Blood Pressure] this term only and with qualifier(s): [drug effects - DE] |
| 8 | ((antihypertens* or anti-hypertens*)):ti,ab,kw |
| 9 | ((("blood pressure" or hypertens*) near/5 (lower* or reduc* or decreas*))):ti,ab,kw |
| 10 | ((angiotensin near/3 convert* near/3 enzyme near/3 (inhibit* or antagonist* or block*))):ti,ab,kw |
| 11 | ((((ace or renin) near/3 inhibit*) or ACEI)):ti,ab,kw |
| 12 | ((angiotensin near/3 receptor* near/3 (inhibit* or antagonist* or block*))):ti,ab,kw |
| 13 | ((calcium near/2 (inhibit* or antagonist* or block*))):ti,ab,kw |
| 14 | ((adrenergic near/3 beta* near/3 (inhibit* or antagonist* or block*))):ti,ab,kw |
| 15 | ((adrenergic near/3 alpha* near/3 (inhibit* or antagonist* or block*))):ti,ab,kw |
| 16 | (((loop or ceiling) next diuretic*)):ti,ab,kw |
| 17 | ((amiloride or benzothiadiazine or bendroflumethiazide or bumetanide or chlorothiazide or cyclopenthiazide or furosemide or hydrochlorothiazide or hydroflumethiazide or methyclothiazide or metolazone or polythiazide or trichlormethiazide or veratide or thiazide*)):ti,ab,kw |
| 18 | ((chlorthalidone or chlortalidone or phthalamudine or chlorphthalidolone or oxodoline or thalitone or hygroton or indapamide or metindamide or "s-1520" or s1520 or "se-1520" or se1520)):ti,ab,kw |
| 19 | ((alacepril or altiopril or benazepril or captopril or ceronapril or cilazapril or delapril or enalapril or fosinopril or idapril or imidapril or lisinopril or moexipril or moveltipril or pentopril or perindopril or quinapril or ramipril or spirapril or temocapril or trandolapril or zofenopril or aliskiren or remikiren)):ti,ab,kw |
| 20 | (("KT3-671" or candesartan or eprosartan or irbesartan or losartan or olmesartan or tasosartan or telmisartan or valsartan)):ti,ab,kw |
| 21 | ((amlodipine or amrinone or bencyclane or bepridil or cinnarizine or conotoxins or diltiazem or felodipine or fendiline or flunarizine or gallopamil or isradipine or lidoflazine or "magnesium sulphate" or mibefradil or nicardipine or nifedipine or nimodipine or nisoldipine or nitrendipine or perhexiline or prenylamine or verapamil or "omega-agatoxin iva" or "omega-conotoxin gvia" or "omega-conotoxins")):ti,ab,kw |
| 22 | ((methyldopa or alphamethyldopa or amodopa or dopamet or dopegyt or dopegit or dopegite or emdopa or hyperpax or hyperpaxa or "methylpropionic acid" or dopergit or meldopa or methyldopate or medopa or medomet or sembrina or aldomet or aldometil or aldomin or hydopa or methyldihydroxyphenylalanine or "methyl dopa" or mulfasin or presinol or presolisin or sedometil or sembrina or taquinil or dihydroxyphenylalanine or methylphenylalanine or methylalanine or "alpha methyl dopa")):ti,ab,kw |
| 23 | ((reserpine or serpentina or rauwolfia or serpasil)):ti,ab,kw |
| 24 | ((clonidine or adesipress or arkamin or caprysin or catapres* or catasan or chlofazolin or chlophazolin or clinidine or clofelin* or clofenil or clomidine or clondine or clonistada or clonnirit or clophelin* or dichlorophenylaminoimidazoline or dixarit or duraclon or gemiton or haemiton or hemiton or imidazoline or isoglaucon or klofelin or klofenil or "m-5041t" or normopresan or paracefan or "st-155" or "st 155" or "tesno timelets")):ti,ab,kw |
| 25 | ((hydralazin* or hydrallazin* or hydralizine or hydrazinophtalazine or hydrazinophthalazine or hydrazinophtalizine or dralzine or hydralacin or hydrolazine or hypophthalin or hypoftalin or hydrazinophthalazine or idralazina or "1-hydrazinophthalazine" or apressin or nepresol or apressoline or apresoline or apresolin or alphapress or alazine or idralazina or lopress or plethorit or praeparat)):ti,ab,kw |
| 26 | ((acebutolol or adimolol or afurolol or alprenolol or amosulalol or arotinolol or atenolol or befunolol or betaxolol or bevantolol or bisoprolol or bopindolol or bornaprolol or brefonalol or bucindolol or bucumolol or bufetolol or bufuralol or bunitrolol or bunolol or bupranolol or butofilolol or butoxamine or carazolol or carteolol or carvedilol or celiprolol or cetamolol or chlortalidone cloranolol or cyanoiodopindolol or cyanopindolol or deacetylmetipranolol or diacetolol or dihydroalprenolol or dilevalol or epanolol or esmolol or exaprolol or falintolol or flestolol or flusoxolol or hydroxybenzylpinodolol or hydroxycarteolol or hydroxymetoprolol or indenolol or iodocyanopindolol or iodopindolol or iprocrolol or isoxaprolol or labetalol or landiolol or levobunolol or levomoprolol or medroxalol or mepindolol or methylthiopropranolol or metipranolol or metoprolol or moprolol or nadolol or oxprenolol or penbutolol or pindolol or nadolol or nebivolol or nifenalol or nipradilol or oxprenolol or pafenolol or pamatolol or penbutolol or pindolol or practolol or primidolol or prizidilol or procinolol or pronetalol or propranolol or proxodolol or ridazolol or salcardolol or soquinolol or sotalol or spirendolol or talinolol or tertatolol or tienoxolol or tilisolol or timolol or tolamolol or toliprolol or tribendilol or xibenolol)):ti,ab,kw |
| 27 | ((alfuzosin or bunazosin or doxazosin or metazosin or neldazosin or prazosin or silodosin or tamsulosin or terazosin or tiodazosin or trimazosin)):ti,ab,kw |
| 28 | {or #5-#27} |
| 29 | (continu* or discontinu* or temporar*):ti,ab,kw |
| 30 | #4 and #28 and #29 |
| 31 | [mh "infant, newborn"] or [mh "pediatrics"] or [mh "adolescent"] |
| 32 | (cardi* or myocard* or heart or coronary):ti,ab,kw |
| 33 | (child* or p?ediat* or neonat* or newborn* or infant* or baby* or babies or toddler* or minors* or adolesc* or preteen* or teen* or juvenil* or youth* or preschool* or school* or kindergarten* or kid*):ti,ab,kw |
| 34 | {or #31-#33} |
| 35 | #30 not #34 |

# PICO 7. In patients with acute intracerebral haemorrhage, does intensive blood pressure lowering with any vasodepressor drug compared to control improve outcome?

Medline (Ovid)

| # | Query |
| --- | --- |
| 1 | exp intracranial hemorrhages/ or exp cerebral hemorrhage/ or exp basal ganglia hemorrhage/ or exp cerebral intraventricular hemorrhage/ or exp intracranial hemorrhage, hypertensive/ or exp hemorrhagic stroke/ |
| 2 | ((brain* or cerebr* or cerebell* or intracerebral or intracran* or parenchymal or intraparenchymal or intraventricular or infratentorial or supratentorial or basal gangli* or putaminal or putamen or posterior fossa or hemispher* or stroke or apoplex*) adj5 (h?emorrhag* or h?ematoma* or bleed*)).tw. |
| 3 | 1 or 2 or (ICH or ICHs).tw. |
| 4 | (acute or sudden or spontaneous).mp. |
| 5 | 3 and 4 |
| 6 | exp antihypertensive agents/ or exp vasodilator agents/ or exp adrenergic agonists/ or exp diuretics/ or exp thiazides/ or exp sodium chloride symporter inhibitors/ or exp sodium potassium chloride symporter inhibitors/ |
| 7 | exp angiotensin-converting enzyme inhibitors/ or exp angiotensin II type 1 receptor blockers/ or exp calcium channel blockers/ or exp adrenergic beta-antagonists/ or exp adrenergic alpha antagonists/ |
| 8 | exp enalapril/ or exp losartan/ or exp hydralazine/ |
| 9 | exp hypertension/ or exp blood pressure/ |
| 10 | (antihyperten* or anti-hypertens*).tw. |
| 11 | ((Blood pressure or hypertens*) adj5 (lower* or reduc* or decreas*)).tw. |
| 12 | (angiotensin adj3 convert* adj3 enzyme adj3 (inhibit* or antagonist? or block*)).tw. |
| 13 | (((ace or renin) adj3 inhibit*) or ACEI).tw. |
| 14 | (angiotensin adj3 receptor? adj3 (inhibit* or antagonist? or block*)).tw. |
| 15 | (calcium adj2 (inhibit* or antagonist? or block*)).tw. |
| 16 | (adrenergic adj3 beta* adj3 (inhibit* or antagonist? or block*)).tw. |
| 17 | (adrenergic adj3 alpha* adj3 (inhibit* or antagonist? or block*)).tw. |
| 18 | ((loop or ceiling) adj diuretic?).tw. |
| 19 | (amiloride or benzothiadiazine or bendroflumethiazide or bumetanide or chlorothiazide or cyclopenthiazide or furosemide or hydrochlorothiazide or hydroflumethiazide or methyclothiazide or metolazone or polythiazide or trichlormethiazide or veratide or thiazide?).mp. |
| 20 | (chlorthalidone or chlortalidone or phthalamudine or chlorphthalidolone or oxodoline or thalitone or hygroton or indapamide or metindamide or s-1520 or s1520 or se-1520 or se1520).mp. |
| 21 | (alacepril or altiopril or benazepril or captopril or ceronapril or cilazapril or delapril or enalapril or fosinopril or idapril or imidapril or lisinopril or moexipril or moveltipril or pentopril or perindopril or quinapril or ramipril or spirapril or temocapril or trandolapril or zofenopril or aliskiren or remikiren).mp. |
| 22 | (KT3-671 or candesartan or eprosartan or irbesartan or losartan or olmesartan or tasosartan or telmisartan or valsartan).mp. |
| 23 | (amlodipine or amrinone or bencyclane or bepridil or cinnarizine or conotoxins or diltiazem or felodipine or fendiline or flunarizine or gallopamil or isradipine or lidoflazine or magnesium sulfate or mibefradil or nicardipine or nifedipine or nimodipine or nisoldipine or nitrendipine or perhexiline or prenylamine or verapamil or omega-agatoxin iva or omega-conotoxin gvia or omega-conotoxins).mp. |
| 24 | (methyldopa or alphamethyldopa or amodopa or dopamet or dopegyt or dopegit or dopegite or emdopa or hyperpax or hyperpaxa or methylpropionic acid or dopergit or meldopa or methyldopate or medopa or medomet or sembrina or aldomet or aldometil or aldomin or hydopa or methyldihydroxyphenylalanine or methyl dopa or mulfasin or presinol or presolisin or sedometil or sembrina or taquinil or dihydroxyphenylalanine or methylphenylalanine or methylalanine or alpha methyl dopa).mp. |
| 25 | (reserpine or serpentina or rauwolfia or serpasil).mp. |
| 26 | (clonidine or adesipress or arkamin or caprysin or catapres* or catasan or chlofazolin or chlophazolin or clinidine or clofelin* or clofenil or clomidine or clondine or clonistada or clonnirit or clophelin* or dichlorophenylaminoimidazoline or dixarit or duraclon or gemiton or haemiton or hemiton or imidazoline or isoglaucon or klofelin or klofenil or m-5041t or normopresan or paracefan or st-155 or st 155 or tesno timelets).mp. |
| 27 | (hydralazin* or hydrallazin* or hydralizine or hydrazinophtalazine or hydrazinophthalazine or hydrazinophtalizine or dralzine or hydralacin or hydrolazine or hypophthalin or hypoftalin or hydrazinophthalazine or idralazina or 1-hydrazinophthalazine or apressin or nepresol or apressoline or apresoline or apresolin or alphapress or alazine or idralazina or lopress or plethorit or praeparat).mp. |
| 28 | (acebutolol or adimolol or afurolol or alprenolol or amosulalol or arotinolol or atenolol or befunolol or betaxolol or bevantolol or bisoprolol or bopindolol or bornaprolol or brefonalol or bucindolol or bucumolol or bufetolol or bufuralol or bunitrolol or bunolol or bupranolol or butofilolol or butoxamine or carazolol or carteolol or carvedilol or celiprolol or cetamolol or chlortalidone cloranolol or cyanoiodopindolol or cyanopindolol or deacetylmetipranolol or diacetolol or dihydroalprenolol or dilevalol or epanolol or esmolol or exaprolol or falintolol or flestolol or flusoxolol or hydroxybenzylpinodolol or hydroxycarteolol or hydroxymetoprolol or indenolol or iodocyanopindolol or iodopindolol or iprocrolol or isoxaprolol or labetalol or landiolol or levobunolol or levomoprolol or medroxalol or mepindolol or methylthiopropranolol or metipranolol or metoprolol or moprolol or nadolol or oxprenolol or penbutolol or pindolol or nadolol or nebivolol or nifenalol or nipradilol or oxprenolol or pafenolol or pamatolol or penbutolol or pindolol or practolol or primidolol or prizidilol or procinolol or pronetalol or propranolol or proxodolol or ridazolol or salcardolol or soquinolol or sotalol or spirendolol or talinolol or tertatolol or tienoxolol or tilisolol or timolol or tolamolol or toliprolol or tribendilol or xibenolol).mp. |
| 29 | (alfuzosin or bunazosin or doxazosin or metazosin or neldazosin or prazosin or silodosin or tamsulosin or terazosin or tiodazosin or trimazosin).mp. |
| 30 | or/6-29 |
| 31 | 5 and 30 |
| 32 | exp randomized controlled trial/ |
| 33 | controlled clinical trial.pt. |
| 34 | randomized.ab. |
| 35 | placebo.ab. |
| 36 | drug therapy.fs. |
| 37 | randomly.ab. |
| 38 | trial.ab. |
| 39 | groups.ab. |
| 40 | or/32-39 |
| 41 | 31 and 40 |
| 42 | exp animals/ not humans.sh. |
| 43 | (cardi* or myocard* or heart or coronary).tw. |
| 44 | exp child/ or adolescent/ or exp infant/ or exp Pediatrics/ or child*.mp. or p?ediat*.mp. or neonat*.mp. or newborn*.mp. or infant*.mp. or baby*.mp. or babies.mp. or toddler*.mp. or minors*.mp. or adolesc*.mp. or preteen*.mp. or teen*.mp. or juvenil*.mp. or youth*.mp. or preschool*.mp. or school*.mp. or kindergarten*.mp. or kid.mp. or kids.mp. |
| 45 | or/42-44 |
| 46 | 41 not 45 |

Embase (Ovid)

| # | Query |
| --- | --- |
| 1 | exp intracranial hemorrhages/ or exp cerebral hemorrhage/ or exp basal ganglia hemorrhage/ or exp cerebral intraventricular hemorrhage/ or exp intracranial hemorrhage, hypertensive/ or exp hemorrhagic stroke/ |
| 2 | ((brain* or cerebr* or cerebell* or intracerebral or intracran* or parenchymal or intraparenchymal or intraventricular or infratentorial or supratentorial or basal gangli* or putaminal or putamen or posterior fossa or hemispher* or stroke or apoplex*) adj5 (h?emorrhag* or h?ematoma* or bleed*)).tw. |
| 3 | 1 or 2 or (ICH or ICHs).tw. |
| 4 | (acute or sudden or spontaneous).mp. |
| 5 | 3 and 4 |
| 6 | exp antihypertensive agents/ or exp vasodilator agents/ or exp adrenergic agonists/ or exp diuretics/ or exp thiazides/ or exp sodium chloride symporter inhibitors/ or exp sodium potassium chloride symporter inhibitors/ |
| 7 | exp angiotensin-converting enzyme inhibitors/ or exp angiotensin II type 1 receptor blockers/ or exp calcium channel blockers/ or exp adrenergic beta-antagonists/ or exp adrenergic alpha antagonists/ |
| 8 | exp enalapril/ or exp losartan/ or exp hydralazine/ |
| 9 | hypertension/ae, de, dt, pc or blood pressure/de, pd |
| 10 | (antihyperten* or anti-hypertens*).tw. |
| 11 | ((Blood pressure or hypertens*) adj5 (lower* or reduc* or decreas*)).tw. |
| 12 | (angiotensin adj3 convert* adj3 enzyme adj3 (inhibit* or antagonist? or block*)).tw. |
| 13 | (((ace or renin) adj3 inhibit*) or ACEI).tw. |
| 14 | (angiotensin adj3 receptor? adj3 (inhibit* or antagonist? or block*)).tw. |
| 15 | (calcium adj2 (inhibit* or antagonist? or block*)).tw. |
| 16 | (adrenergic adj3 beta* adj3 (inhibit* or antagonist? or block*)).tw. |
| 17 | (adrenergic adj3 alpha* adj3 (inhibit* or antagonist? or block*)).tw. |
| 18 | ((loop or ceiling) adj diuretic?).tw. |
| 19 | (amiloride or benzothiadiazine or bendroflumethiazide or bumetanide or chlorothiazide or cyclopenthiazide or furosemide or hydrochlorothiazide or hydroflumethiazide or methyclothiazide or metolazone or polythiazide or trichlormethiazide or veratide or thiazide?).mp. |
| 20 | (chlorthalidone or chlortalidone or phthalamudine or chlorphthalidolone or oxodoline or thalitone or hygroton or indapamide or metindamide or s-1520 or s1520 or se-1520 or se1520).mp. |
| 21 | (alacepril or altiopril or benazepril or captopril or ceronapril or cilazapril or delapril or enalapril or fosinopril or idapril or imidapril or lisinopril or moexipril or moveltipril or pentopril or perindopril or quinapril or ramipril or spirapril or temocapril or trandolapril or zofenopril or aliskiren or remikiren).mp. |
| 22 | (KT3-671 or candesartan or eprosartan or irbesartan or losartan or olmesartan or tasosartan or telmisartan or valsartan).mp. |
| 23 | (amlodipine or amrinone or bencyclane or bepridil or cinnarizine or conotoxins or diltiazem or felodipine or fendiline or flunarizine or gallopamil or isradipine or lidoflazine or magnesium sulfate or mibefradil or nicardipine or nifedipine or nimodipine or nisoldipine or nitrendipine or perhexiline or prenylamine or verapamil or omega-agatoxin iva or omega-conotoxin gvia or omega-conotoxins).mp. |
| 24 | (methyldopa or alphamethyldopa or amodopa or dopamet or dopegyt or dopegit or dopegite or emdopa or hyperpax or hyperpaxa or methylpropionic acid or dopergit or meldopa or methyldopate or medopa or medomet or sembrina or aldomet or aldometil or aldomin or hydopa or methyldihydroxyphenylalanine or methyl dopa or mulfasin or presinol or presolisin or sedometil or sembrina or taquinil or dihydroxyphenylalanine or methylphenylalanine or methylalanine or alpha methyl dopa).mp. |
| 25 | (reserpine or serpentina or rauwolfia or serpasil).mp. |
| 26 | (clonidine or adesipress or arkamin or caprysin or catapres* or catasan or chlofazolin or chlophazolin or clinidine or clofelin* or clofenil or clomidine or clondine or clonistada or clonnirit or clophelin* or dichlorophenylaminoimidazoline or dixarit or duraclon or gemiton or haemiton or hemiton or imidazoline or isoglaucon or klofelin or klofenil or m-5041t or normopresan or paracefan or st-155 or st 155 or tesno timelets).mp. |
| 27 | (hydralazin* or hydrallazin* or hydralizine or hydrazinophtalazine or hydrazinophthalazine or hydrazinophtalizine or dralzine or hydralacin or hydrolazine or hypophthalin or hypoftalin or hydrazinophthalazine or idralazina or 1-hydrazinophthalazine or apressin or nepresol or apressoline or apresoline or apresolin or alphapress or alazine or idralazina or lopress or plethorit or praeparat).mp. |
| 28 | (acebutolol or adimolol or afurolol or alprenolol or amosulalol or arotinolol or atenolol or befunolol or betaxolol or bevantolol or bisoprolol or bopindolol or bornaprolol or brefonalol or bucindolol or bucumolol or bufetolol or bufuralol or bunitrolol or bunolol or bupranolol or butofilolol or butoxamine or carazolol or carteolol or carvedilol or celiprolol or cetamolol or chlortalidone cloranolol or cyanoiodopindolol or cyanopindolol or deacetylmetipranolol or diacetolol or dihydroalprenolol or dilevalol or epanolol or esmolol or exaprolol or falintolol or flestolol or flusoxolol or hydroxybenzylpinodolol or hydroxycarteolol or hydroxymetoprolol or indenolol or iodocyanopindolol or iodopindolol or iprocrolol or isoxaprolol or labetalol or landiolol or levobunolol or levomoprolol or medroxalol or mepindolol or methylthiopropranolol or metipranolol or metoprolol or moprolol or nadolol or oxprenolol or penbutolol or pindolol or nadolol or nebivolol or nifenalol or nipradilol or oxprenolol or pafenolol or pamatolol or penbutolol or pindolol or practolol or primidolol or prizidilol or procinolol or pronetalol or propranolol or proxodolol or ridazolol or salcardolol or soquinolol or sotalol or spirendolol or talinolol or tertatolol or tienoxolol or tilisolol or timolol or tolamolol or toliprolol or tribendilol or xibenolol).mp. |
| 29 | (alfuzosin or bunazosin or doxazosin or metazosin or neldazosin or prazosin or silodosin or tamsulosin or terazosin or tiodazosin or trimazosin).mp. |
| 30 | or/6-29 |
| 31 | exp randomized controlled trial/ |
| 32 | controlled clinical trial/ |
| 33 | random*.ti,ab. |
| 34 | randomization/ |
| 35 | intermethod comparison/ |
| 36 | placebo.ti,ab. |
| 37 | (compare or compared or comparison).ti,ab. |
| 38 | ((evaluated or evaluate or evaluating or assessed or assess) and (compare or compared or comparing or comparison)).ab. |
| 39 | (open adj label).ti,ab. |
| 40 | ((double or single or doubly or singly) adj (blind or blinded or blindly)).ti,ab. |
| 41 | double blind procedure/ |
| 42 | parallel group*1.ti,ab. |
| 43 | (crossover or cross over).ti,ab. |
| 44 | ((assign* or match or matched or allocation) adj5 (alternate or group*1 or intervention*1 or patient*1 or subject*1 or participant*1)).ti,ab. |
| 45 | (assigned or allocated).ti,ab. |
| 46 | (controlled adj7 (study or design or trial)).ti,ab. |
| 47 | (volunteer or volunteers).ti,ab. |
| 48 | human experiment/ |
| 49 | trial.ti. |
| 50 | or/31-49 |
| 51 | (random* adj sampl* adj7 ("cross section*" or questionnaire*1 or survey* or database*1)).ti,ab. not (comparative study/ or controlled study/ or randomi?ed controlled.ti,ab. or randomly assigned.ti,ab.) |
| 52 | cross-sectional study/ not (exp randomized controlled trial/ or controlled clinical trial/ or controlled study/ or randomi?ed controlled.ti,ab. or control group*1.ti,ab.) |
| 53 | (((case adj control*) and random*) not randomi?ed controlled).ti,ab. |
| 54 | systematic review.ti,ab. not (trial or study).ti. |
| 55 | (non random* not random*).ti,ab. |
| 56 | "random field*".ti,ab. |
| 57 | (random cluster adj3 sample*).ti,ab. |
| 58 | (review.ab. and review.pt.) not trial.ti. |
| 59 | "we searched".ab. and (review.ti. or review.pt.) |
| 60 | "update review".ab. |
| 61 | (databases adj4 searched).ab. |
| 62 | (rat or rats or mouse or mice or swine or porcine or murine or sheep or lambs or pigs or piglets or rabbit or rabbits or cat or cats or dog or dogs or cattle or bovine or monkey or monkeys or trout or marmoset*1).ti. and animal experiment/ |
| 63 | animal experiment/ not (human experiment/ or human/) |
| 64 | (cardi* or myocard* or heart or coronary).tw. |
| 65 | exp child/ or adolescent/ or exp infant/ or exp Pediatrics/ or child*.mp. or p?ediat*.mp. or neonat*.mp. or newborn*.mp. or infant*.mp. or baby*.mp. or babies.mp. or toddler*.mp. or minors*.mp. or adolesc*.mp. or preteen*.mp. or teen*.mp. or juvenil*.mp. or youth*.mp. or preschool*.mp. or school*.mp. or kindergarten*.mp. or kid.mp. or kids.mp. |
| 66 | or/51-65 |
| 67 | 50 not 66 |
| 69 | 5 and 30 and 67 |

Cochrane Library

| # | Query |
| --- | --- |
| 1 | [mh "intracranial hemorrhages"] or [mh "cerebral hemorrhage"] or [mh "basal ganglia hemorrhage"] or [mh "cerebral intraventricular hemorrhage"] or [mh "intracranial hemorrhage, hypertensive"] or [mh "hemorrhagic stroke"] |
| 2 | (((supratentorial or telencephalon or cerebrum or intracerebral or deep or lobar or non-lobar or putaminal or basal ganglia* or thalamus or thalamic or claustrum or striatum or internal capsule or external capsule or globus pallidus or neostriatum or caudate nucleus or putamen or nucleus accumbens or ((cerebral or frontal or prefrontal or parietal or temporal or occipital) near/3 (cortex or lobe or area or centre or center)) or small vessel disease or amyloid angiopathy or hypertensive) near/3 (h?ematoma* or h?emorrhag* or bleed* or stroke*))):ti,ab,kw |
| 3 | (ICH or ICHs):ti,ab,kw |
| 4 | {or #1-#3} |
| 5 | (acute or sudden or spontaneous):ti,ab,kw |
| 6 | #4 and #5 |
| 7 | [mh "antihypertensive agents"] or [mh "vasodilator agents"] or [mh "adrenergic agonists"] or [mh "diuretics"] or [mh "thiazides"] or [mh "sodium chloride symporter inhibitors"] or [mh "sodium potassium chloride symporter inhibitors"] or [mh "angiotensin-converting enzyme inhibitors"] or [mh "angiotensin ii type 1 receptor blockers"] or [mh "calcium channel blockers"] or [mh "adrenergic beta-antagonists"] or [mh "adrenergic alpha-antagonists"] or [mh "enalapril"] or [mh "losartan"] or [mh "hydralazine"] |
| 8 | MeSH descriptor: [Hypertension] this term only and with qualifier(s): [drug therapy - DT, prevention & control - PC] |
| 9 | MeSH descriptor: [Blood Pressure] this term only and with qualifier(s): [drug effects - DE] |
| 10 | ((antihypertens* or anti-hypertens*)):ti,ab,kw |
| 11 | ((("blood pressure" or hypertens*) near/5 (lower* or reduc* or decreas*))):ti,ab,kw |
| 12 | ((angiotensin near/3 convert* near/3 enzyme near/3 (inhibit* or antagonist* or block*))):ti,ab,kw |
| 13 | ((((ace or renin) near/3 inhibit*) or ACEI)):ti,ab,kw |
| 14 | ((angiotensin near/3 receptor* near/3 (inhibit* or antagonist* or block*))):ti,ab,kw |
| 15 | ((calcium near/2 (inhibit* or antagonist* or block*))):ti,ab,kw |
| 16 | ((adrenergic near/3 beta* near/3 (inhibit* or antagonist* or block*))):ti,ab,kw |
| 17 | ((adrenergic near/3 alpha* near/3 (inhibit* or antagonist* or block*))):ti,ab,kw |
| 18 | (((loop or ceiling) next diuretic*)):ti,ab,kw |
| 19 | ((amiloride or benzothiadiazine or bendroflumethiazide or bumetanide or chlorothiazide or cyclopenthiazide or furosemide or hydrochlorothiazide or hydroflumethiazide or methyclothiazide or metolazone or polythiazide or trichlormethiazide or veratide or thiazide*)):ti,ab,kw |
| 20 | ((chlorthalidone or chlortalidone or phthalamudine or chlorphthalidolone or oxodoline or thalitone or hygroton or indapamide or metindamide or "s-1520" or s1520 or "se-1520" or se1520)):ti,ab,kw |
| 21 | ((alacepril or altiopril or benazepril or captopril or ceronapril or cilazapril or delapril or enalapril or fosinopril or idapril or imidapril or lisinopril or moexipril or moveltipril or pentopril or perindopril or quinapril or ramipril or spirapril or temocapril or trandolapril or zofenopril or aliskiren or remikiren)):ti,ab,kw |
| 22 | (("KT3-671" or candesartan or eprosartan or irbesartan or losartan or olmesartan or tasosartan or telmisartan or valsartan)):ti,ab,kw |
| 23 | ((amlodipine or amrinone or bencyclane or bepridil or cinnarizine or conotoxins or diltiazem or felodipine or fendiline or flunarizine or gallopamil or isradipine or lidoflazine or "magnesium sulphate" or mibefradil or nicardipine or nifedipine or nimodipine or nisoldipine or nitrendipine or perhexiline or prenylamine or verapamil or "omega-agatoxin iva" or "omega-conotoxin gvia" or "omega-conotoxins")):ti,ab,kw |
| 24 | ((methyldopa or alphamethyldopa or amodopa or dopamet or dopegyt or dopegit or dopegite or emdopa or hyperpax or hyperpaxa or "methylpropionic acid" or dopergit or meldopa or methyldopate or medopa or medomet or sembrina or aldomet or aldometil or aldomin or hydopa or methyldihydroxyphenylalanine or "methyl dopa" or mulfasin or presinol or presolisin or sedometil or sembrina or taquinil or dihydroxyphenylalanine or methylphenylalanine or methylalanine or "alpha methyl dopa")):ti,ab,kw |
| 25 | ((reserpine or serpentina or rauwolfia or serpasil)):ti,ab,kw |
| 26 | ((clonidine or adesipress or arkamin or caprysin or catapres* or catasan or chlofazolin or chlophazolin or clinidine or clofelin* or clofenil or clomidine or clondine or clonistada or clonnirit or clophelin* or dichlorophenylaminoimidazoline or dixarit or duraclon or gemiton or haemiton or hemiton or imidazoline or isoglaucon or klofelin or klofenil or "m-5041t" or normopresan or paracefan or "st-155" or "st 155" or "tesno timelets")):ti,ab,kw |
| 27 | ((hydralazin* or hydrallazin* or hydralizine or hydrazinophtalazine or hydrazinophthalazine or hydrazinophtalizine or dralzine or hydralacin or hydrolazine or hypophthalin or hypoftalin or hydrazinophthalazine or idralazina or "1-hydrazinophthalazine" or apressin or nepresol or apressoline or apresoline or apresolin or alphapress or alazine or idralazina or lopress or plethorit or praeparat)):ti,ab,kw |
| 28 | ((acebutolol or adimolol or afurolol or alprenolol or amosulalol or arotinolol or atenolol or befunolol or betaxolol or bevantolol or bisoprolol or bopindolol or bornaprolol or brefonalol or bucindolol or bucumolol or bufetolol or bufuralol or bunitrolol or bunolol or bupranolol or butofilolol or butoxamine or carazolol or carteolol or carvedilol or celiprolol or cetamolol or chlortalidone cloranolol or cyanoiodopindolol or cyanopindolol or deacetylmetipranolol or diacetolol or dihydroalprenolol or dilevalol or epanolol or esmolol or exaprolol or falintolol or flestolol or flusoxolol or hydroxybenzylpinodolol or hydroxycarteolol or hydroxymetoprolol or indenolol or iodocyanopindolol or iodopindolol or iprocrolol or isoxaprolol or labetalol or landiolol or levobunolol or levomoprolol or medroxalol or mepindolol or methylthiopropranolol or metipranolol or metoprolol or moprolol or nadolol or oxprenolol or penbutolol or pindolol or nadolol or nebivolol or nifenalol or nipradilol or oxprenolol or pafenolol or pamatolol or penbutolol or pindolol or practolol or primidolol or prizidilol or procinolol or pronetalol or propranolol or proxodolol or ridazolol or salcardolol or soquinolol or sotalol or spirendolol or talinolol or tertatolol or tienoxolol or tilisolol or timolol or tolamolol or toliprolol or tribendilol or xibenolol)):ti,ab,kw |
| 29 | ((alfuzosin or bunazosin or doxazosin or metazosin or neldazosin or prazosin or silodosin or tamsulosin or terazosin or tiodazosin or trimazosin)):ti,ab,kw |
| 30 | {or #7-#29} |
| 31 | #6 and #30 |
| 32 | [mh "infant, newborn"] or [mh "pediatrics"] or [mh "adolescent"] |
| 33 | (cardi* or myocard* or heart or coronary):ti,ab,kw |
| 34 | (child* or p?ediat* or neonat* or newborn* or infant* or baby* or babies or toddler* or minors* or adolesc* or preteen* or teen* or juvenil* or youth* or preschool* or school* or kindergarten* or kid*):ti,ab,kw |
| 33 | {or #32-#34} |
| 34 | #31 and #33 |

# PICO 8. In patients with acute intracerebral haemorrhage, does continuing versus temporarily stopping previous oral antihypertensive therapy improve outcome?

Medline (Ovid)

| # | Query |
| --- | --- |
| 1 | exp intracranial hemorrhages/ or exp cerebral hemorrhage/ or exp basal ganglia hemorrhage/ or exp cerebral intraventricular hemorrhage/ or exp intracranial hemorrhage, hypertensive/ or exp hemorrhagic stroke/ |
| 2 | ((brain* or cerebr* or cerebell* or intracerebral or intracran* or parenchymal or intraparenchymal or intraventricular or infratentorial or supratentorial or basal gangli* or putaminal or putamen or posterior fossa or hemispher* or stroke or apoplex*) adj5 (h?emorrhag* or h?ematoma* or bleed*)).tw. |
| 3 | 1 or 2 or (ICH or ICHs).tw. |
| 4 | exp antihypertensive agents/ or exp vasodilator agents/ or exp adrenergic agonists/ or exp diuretics/ or exp thiazides/ or exp sodium chloride symporter inhibitors/ or exp sodium potassium chloride symporter inhibitors/ |
| 5 | exp angiotensin-converting enzyme inhibitors/ or exp angiotensin II type 1 receptor blockers/ or exp calcium channel blockers/ or exp adrenergic beta-antagonists/ or exp adrenergic alpha antagonists/ |
| 6 | exp enalapril/ or exp losartan/ or exp hydralazine/ |
| 7 | exp hypertension/ or exp blood pressure/ |
| 8 | (antihyperten* or anti-hypertens*).tw. |
| 9 | ((Blood pressure or hypertens*) adj5 (lower* or reduc* or decreas*)).tw. |
| 10 | (angiotensin adj3 convert* adj3 enzyme adj3 (inhibit* or antagonist? or block*)).tw. |
| 11 | (((ace or renin) adj3 inhibit*) or ACEI).tw. |
| 12 | (angiotensin adj3 receptor? adj3 (inhibit* or antagonist? or block*)).tw. |
| 13 | (calcium adj2 (inhibit* or antagonist? or block*)).tw. |
| 14 | (adrenergic adj3 beta* adj3 (inhibit* or antagonist? or block*)).tw. |
| 15 | (adrenergic adj3 alpha* adj3 (inhibit* or antagonist? or block*)).tw. |
| 16 | ((loop or ceiling) adj diuretic?).tw. |
| 17 | (amiloride or benzothiadiazine or bendroflumethiazide or bumetanide or chlorothiazide or cyclopenthiazide or furosemide or hydrochlorothiazide or hydroflumethiazide or methyclothiazide or metolazone or polythiazide or trichlormethiazide or veratide or thiazide?).mp. |
| 18 | (chlorthalidone or chlortalidone or phthalamudine or chlorphthalidolone or oxodoline or thalitone or hygroton or indapamide or metindamide or s-1520 or s1520 or se-1520 or se1520).mp. |
| 19 | (alacepril or altiopril or benazepril or captopril or ceronapril or cilazapril or delapril or enalapril or fosinopril or idapril or imidapril or lisinopril or moexipril or moveltipril or pentopril or perindopril or quinapril or ramipril or spirapril or temocapril or trandolapril or zofenopril or aliskiren or remikiren).mp. |
| 20 | (KT3-671 or candesartan or eprosartan or irbesartan or losartan or olmesartan or tasosartan or telmisartan or valsartan).mp. |
| 21 | (amlodipine or amrinone or bencyclane or bepridil or cinnarizine or conotoxins or diltiazem or felodipine or fendiline or flunarizine or gallopamil or isradipine or lidoflazine or magnesium sulfate or mibefradil or nicardipine or nifedipine or nimodipine or nisoldipine or nitrendipine or perhexiline or prenylamine or verapamil or omega-agatoxin iva or omega-conotoxin gvia or omega-conotoxins).mp. |
| 22 | (methyldopa or alphamethyldopa or amodopa or dopamet or dopegyt or dopegit or dopegite or emdopa or hyperpax or hyperpaxa or methylpropionic acid or dopergit or meldopa or methyldopate or medopa or medomet or sembrina or aldomet or aldometil or aldomin or hydopa or methyldihydroxyphenylalanine or methyl dopa or mulfasin or presinol or presolisin or sedometil or sembrina or taquinil or dihydroxyphenylalanine or methylphenylalanine or methylalanine or alpha methyl dopa).mp. |
| 23 | (reserpine or serpentina or rauwolfia or serpasil).mp. |
| 24 | (clonidine or adesipress or arkamin or caprysin or catapres* or catasan or chlofazolin or chlophazolin or clinidine or clofelin* or clofenil or clomidine or clondine or clonistada or clonnirit or clophelin* or dichlorophenylaminoimidazoline or dixarit or duraclon or gemiton or haemiton or hemiton or imidazoline or isoglaucon or klofelin or klofenil or m-5041t or normopresan or paracefan or st-155 or st 155 or tesno timelets).mp. |
| 25 | (hydralazin* or hydrallazin* or hydralizine or hydrazinophtalazine or hydrazinophthalazine or hydrazinophtalizine or dralzine or hydralacin or hydrolazine or hypophthalin or hypoftalin or hydrazinophthalazine or idralazina or 1-hydrazinophthalazine or apressin or nepresol or apressoline or apresoline or apresolin or alphapress or alazine or idralazina or lopress or plethorit or praeparat).mp. |
| 26 | (acebutolol or adimolol or afurolol or alprenolol or amosulalol or arotinolol or atenolol or befunolol or betaxolol or bevantolol or bisoprolol or bopindolol or bornaprolol or brefonalol or bucindolol or bucumolol or bufetolol or bufuralol or bunitrolol or bunolol or bupranolol or butofilolol or butoxamine or carazolol or carteolol or carvedilol or celiprolol or cetamolol or chlortalidone cloranolol or cyanoiodopindolol or cyanopindolol or deacetylmetipranolol or diacetolol or dihydroalprenolol or dilevalol or epanolol or esmolol or exaprolol or falintolol or flestolol or flusoxolol or hydroxybenzylpinodolol or hydroxycarteolol or hydroxymetoprolol or indenolol or iodocyanopindolol or iodopindolol or iprocrolol or isoxaprolol or labetalol or landiolol or levobunolol or levomoprolol or medroxalol or mepindolol or methylthiopropranolol or metipranolol or metoprolol or moprolol or nadolol or oxprenolol or penbutolol or pindolol or nadolol or nebivolol or nifenalol or nipradilol or oxprenolol or pafenolol or pamatolol or penbutolol or pindolol or practolol or primidolol or prizidilol or procinolol or pronetalol or propranolol or proxodolol or ridazolol or salcardolol or soquinolol or sotalol or spirendolol or talinolol or tertatolol or tienoxolol or tilisolol or timolol or tolamolol or toliprolol or tribendilol or xibenolol).mp. |
| 27 | (alfuzosin or bunazosin or doxazosin or metazosin or neldazosin or prazosin or silodosin or tamsulosin or terazosin or tiodazosin or trimazosin).mp. |
| 28 | or/4-27 |
| 29 | (continu* or discontinu* or temporar* or short-term or shortterm).mp. |
| 30 | 4 and 28 and 29 |
| 31 | exp randomized controlled trial/ |
| 32 | controlled clinical trial.pt. |
| 33 | randomized.ab. |
| 34 | placebo.ab. |
| 35 | drug therapy.fs. |
| 36 | randomly.ab. |
| 37 | trial.ab. |
| 38 | groups.ab. |
| 39 | or/31-38 |
| 40 | 30 and 39 |
| 41 | exp animals/ not humans.sh. |
| 42 | (cardi* or myocard* or heart or coronary).tw. |
| 43 | exp child/ or adolescent/ or exp infant/ or exp Pediatrics/ or child*.mp. or p?ediat*.mp. or neonat*.mp. or newborn*.mp. or infant*.mp. or baby*.mp. or babies.mp. or toddler*.mp. or minors*.mp. or adolesc*.mp. or preteen*.mp. or teen*.mp. or juvenil*.mp. or youth*.mp. or preschool*.mp. or school*.mp. or kindergarten*.mp. or kid.mp. or kids.mp. |
| 44 | or/41-43 |
| 45 | 40 not 44 |

Embase (Ovid)

| # | Query |
| --- | --- |
| 1 | exp intracranial hemorrhages/ or exp cerebral hemorrhage/ or exp basal ganglia hemorrhage/ or exp cerebral intraventricular hemorrhage/ or exp intracranial hemorrhage, hypertensive/ or exp hemorrhagic stroke/ |
| 2 | ((brain* or cerebr* or cerebell* or intracerebral or intracran* or parenchymal or intraparenchymal or intraventricular or infratentorial or supratentorial or basal gangli* or putaminal or putamen or posterior fossa or hemispher* or stroke or apoplex*) adj5 (h?emorrhag* or h?ematoma* or bleed*)).tw. |
| 3 | 1 or 2 or (ICH or ICHs).tw. |
| 4 | (acute or sudden or spontaneous).mp. |
| 5 | 3 and 4 |
| 6 | exp antihypertensive agents/ or exp vasodilator agents/ or exp adrenergic agonists/ or exp diuretics/ or exp thiazides/ or exp sodium chloride symporter inhibitors/ or exp sodium potassium chloride symporter inhibitors/ |
| 7 | exp angiotensin-converting enzyme inhibitors/ or exp angiotensin II type 1 receptor blockers/ or exp calcium channel blockers/ or exp adrenergic beta-antagonists/ or exp adrenergic alpha antagonists/ |
| 8 | exp enalapril/ or exp losartan/ or exp hydralazine/ |
| 9 | hypertension/ae, de, dt, pc or blood pressure/de, pd |
| 10 | (antihyperten* or anti-hypertens*).tw. |
| 11 | ((Blood pressure or hypertens*) adj5 (lower* or reduc* or decreas*)).tw. |
| 12 | (angiotensin adj3 convert* adj3 enzyme adj3 (inhibit* or antagonist? or block*)).tw. |
| 13 | (((ace or renin) adj3 inhibit*) or ACEI).tw. |
| 14 | (angiotensin adj3 receptor? adj3 (inhibit* or antagonist? or block*)).tw. |
| 15 | (calcium adj2 (inhibit* or antagonist? or block*)).tw. |
| 16 | (adrenergic adj3 beta* adj3 (inhibit* or antagonist? or block*)).tw. |
| 17 | (adrenergic adj3 alpha* adj3 (inhibit* or antagonist? or block*)).tw. |
| 18 | ((loop or ceiling) adj diuretic?).tw. |
| 19 | (amiloride or benzothiadiazine or bendroflumethiazide or bumetanide or chlorothiazide or cyclopenthiazide or furosemide or hydrochlorothiazide or hydroflumethiazide or methyclothiazide or metolazone or polythiazide or trichlormethiazide or veratide or thiazide?).mp. |
| 20 | (chlorthalidone or chlortalidone or phthalamudine or chlorphthalidolone or oxodoline or thalitone or hygroton or indapamide or metindamide or s-1520 or s1520 or se-1520 or se1520).mp. |
| 21 | (alacepril or altiopril or benazepril or captopril or ceronapril or cilazapril or delapril or enalapril or fosinopril or idapril or imidapril or lisinopril or moexipril or moveltipril or pentopril or perindopril or quinapril or ramipril or spirapril or temocapril or trandolapril or zofenopril or aliskiren or remikiren).mp. |
| 22 | (KT3-671 or candesartan or eprosartan or irbesartan or losartan or olmesartan or tasosartan or telmisartan or valsartan).mp. |
| 23 | (amlodipine or amrinone or bencyclane or bepridil or cinnarizine or conotoxins or diltiazem or felodipine or fendiline or flunarizine or gallopamil or isradipine or lidoflazine or magnesium sulfate or mibefradil or nicardipine or nifedipine or nimodipine or nisoldipine or nitrendipine or perhexiline or prenylamine or verapamil or omega-agatoxin iva or omega-conotoxin gvia or omega-conotoxins).mp. |
| 24 | (methyldopa or alphamethyldopa or amodopa or dopamet or dopegyt or dopegit or dopegite or emdopa or hyperpax or hyperpaxa or methylpropionic acid or dopergit or meldopa or methyldopate or medopa or medomet or sembrina or aldomet or aldometil or aldomin or hydopa or methyldihydroxyphenylalanine or methyl dopa or mulfasin or presinol or presolisin or sedometil or sembrina or taquinil or dihydroxyphenylalanine or methylphenylalanine or methylalanine or alpha methyl dopa).mp. |
| 25 | (reserpine or serpentina or rauwolfia or serpasil).mp. |
| 26 | (clonidine or adesipress or arkamin or caprysin or catapres* or catasan or chlofazolin or chlophazolin or clinidine or clofelin* or clofenil or clomidine or clondine or clonistada or clonnirit or clophelin* or dichlorophenylaminoimidazoline or dixarit or duraclon or gemiton or haemiton or hemiton or imidazoline or isoglaucon or klofelin or klofenil or m-5041t or normopresan or paracefan or st-155 or st 155 or tesno timelets).mp. |
| 27 | (hydralazin* or hydrallazin* or hydralizine or hydrazinophtalazine or hydrazinophthalazine or hydrazinophtalizine or dralzine or hydralacin or hydrolazine or hypophthalin or hypoftalin or hydrazinophthalazine or idralazina or 1-hydrazinophthalazine or apressin or nepresol or apressoline or apresoline or apresolin or alphapress or alazine or idralazina or lopress or plethorit or praeparat).mp. |
| 28 | (acebutolol or adimolol or afurolol or alprenolol or amosulalol or arotinolol or atenolol or befunolol or betaxolol or bevantolol or bisoprolol or bopindolol or bornaprolol or brefonalol or bucindolol or bucumolol or bufetolol or bufuralol or bunitrolol or bunolol or bupranolol or butofilolol or butoxamine or carazolol or carteolol or carvedilol or celiprolol or cetamolol or chlortalidone cloranolol or cyanoiodopindolol or cyanopindolol or deacetylmetipranolol or diacetolol or dihydroalprenolol or dilevalol or epanolol or esmolol or exaprolol or falintolol or flestolol or flusoxolol or hydroxybenzylpinodolol or hydroxycarteolol or hydroxymetoprolol or indenolol or iodocyanopindolol or iodopindolol or iprocrolol or isoxaprolol or labetalol or landiolol or levobunolol or levomoprolol or medroxalol or mepindolol or methylthiopropranolol or metipranolol or metoprolol or moprolol or nadolol or oxprenolol or penbutolol or pindolol or nadolol or nebivolol or nifenalol or nipradilol or oxprenolol or pafenolol or pamatolol or penbutolol or pindolol or practolol or primidolol or prizidilol or procinolol or pronetalol or propranolol or proxodolol or ridazolol or salcardolol or soquinolol or sotalol or spirendolol or talinolol or tertatolol or tienoxolol or tilisolol or timolol or tolamolol or toliprolol or tribendilol or xibenolol).mp. |
| 29 | (alfuzosin or bunazosin or doxazosin or metazosin or neldazosin or prazosin or silodosin or tamsulosin or terazosin or tiodazosin or trimazosin).mp. |
| 30 | or/6-29 |
| 31 | (continu* or discontinu* or temporar*).ti,ab. |
| 32 | 5 and 30 and 31 |
| 33 | exp randomized controlled trial/ |
| 34 | controlled clinical trial/ |
| 35 | random*.ti,ab. |
| 36 | randomization/ |
| 37 | intermethod comparison/ |
| 38 | placebo.ti,ab. |
| 39 | (compare or compared or comparison).ti,ab. |
| 40 | ((evaluated or evaluate or evaluating or assessed or assess) and (compare or compared or comparing or comparison)).ab. |
| 41 | (open adj label).ti,ab. |
| 42 | ((double or single or doubly or singly) adj (blind or blinded or blindly)).ti,ab. |
| 43 | double blind procedure/ |
| 44 | parallel group*1.ti,ab. |
| 45 | (crossover or cross over).ti,ab. |
| 46 | ((assign* or match or matched or allocation) adj5 (alternate or group*1 or intervention*1 or patient*1 or subject*1 or participant*1)).ti,ab. |
| 47 | (assigned or allocated).ti,ab. |
| 48 | (controlled adj7 (study or design or trial)).ti,ab. |
| 49 | (volunteer or volunteers).ti,ab. |
| 50 | human experiment/ |
| 51 | trial.ti. |
| 52 | or/33-51 |
| 53 | (random* adj sampl* adj7 ("cross section*" or questionnaire*1 or survey* or database*1)).ti,ab. not (comparative study/ or controlled study/ or randomi?ed controlled.ti,ab. or randomly assigned.ti,ab.) |
| 54 | cross-sectional study/ not (exp randomized controlled trial/ or controlled clinical trial/ or controlled study/ or randomi?ed controlled.ti,ab. or control group*1.ti,ab.) |
| 55 | (((case adj control*) and random*) not randomi?ed controlled).ti,ab. |
| 56 | systematic review.ti,ab. not (trial or study).ti. |
| 57 | (non random* not random*).ti,ab. |
| 58 | "random field*".ti,ab. |
| 59 | (random cluster adj3 sample*).ti,ab. |
| 60 | (review.ab. and review.pt.) not trial.ti. |
| 61 | "we searched".ab. and (review.ti. or review.pt.) |
| 62 | "update review".ab. |
| 63 | (databases adj4 searched).ab. |
| 64 | (rat or rats or mouse or mice or swine or porcine or murine or sheep or lambs or pigs or piglets or rabbit or rabbits or cat or cats or dog or dogs or cattle or bovine or monkey or monkeys or trout or marmoset*1).ti. and animal experiment/ |
| 65 | animal experiment/ not (human experiment/ or human/) |
| 66 | (cardi* or myocard* or heart or coronary).tw. |
| 67 | exp child/ or adolescent/ or exp infant/ or exp Pediatrics/ or child*.mp. or p?ediat*.mp. or neonat*.mp. or newborn*.mp. or infant*.mp. or baby*.mp. or babies.mp. or toddler*.mp. or minors*.mp. or adolesc*.mp. or preteen*.mp. or teen*.mp. or juvenil*.mp. or youth*.mp. or preschool*.mp. or school*.mp. or kindergarten*.mp. or kid.mp. or kids.mp. |
| 68 | or/53-67 |
| 69 | 52 not 68 |
| 70 | 32 and 69 |

Cochrane Library

| # | Query |
| --- | --- |
| 1 | [mh "intracranial hemorrhages"] or [mh "cerebral hemorrhage"] or [mh "basal ganglia hemorrhage"] or [mh "cerebral intraventricular hemorrhage"] or [mh "intracranial hemorrhage, hypertensive"] or [mh "hemorrhagic stroke"] |
| 2 | (((supratentorial or telencephalon or cerebrum or intracerebral or deep or lobar or non-lobar or putaminal or basal ganglia* or thalamus or thalamic or claustrum or striatum or internal capsule or external capsule or globus pallidus or neostriatum or caudate nucleus or putamen or nucleus accumbens or ((cerebral or frontal or prefrontal or parietal or temporal or occipital) near/3 (cortex or lobe or area or centre or center)) or small vessel disease or amyloid angiopathy or hypertensive) near/3 (h?ematoma* or h?emorrhag* or bleed* or stroke*))):ti,ab,kw |
| 3 | (ICH or ICHs):ti,ab,kw |
| 4 | {or #1-#3} |
| 5 | [mh "antihypertensive agents"] or [mh "vasodilator agents"] or [mh "adrenergic agonists"] or [mh "diuretics"] or [mh "thiazides"] or [mh "sodium chloride symporter inhibitors"] or [mh "sodium potassium chloride symporter inhibitors"] or [mh "angiotensin-converting enzyme inhibitors"] or [mh "angiotensin ii type 1 receptor blockers"] or [mh "calcium channel blockers"] or [mh "adrenergic beta-antagonists"] or [mh "adrenergic alpha-antagonists"] or [mh "enalapril"] or [mh "losartan"] or [mh "hydralazine"] |
| 6 | MeSH descriptor: [Hypertension] this term only and with qualifier(s): [drug therapy - DT, prevention & control - PC] |
| 7 | MeSH descriptor: [Blood Pressure] this term only and with qualifier(s): [drug effects - DE] |
| 8 | ((antihypertens* or anti-hypertens*)):ti,ab,kw |
| 9 | ((("blood pressure" or hypertens*) near/5 (lower* or reduc* or decreas*))):ti,ab,kw |
| 10 | ((angiotensin near/3 convert* near/3 enzyme near/3 (inhibit* or antagonist* or block*))):ti,ab,kw |
| 11 | ((((ace or renin) near/3 inhibit*) or ACEI)):ti,ab,kw |
| 12 | ((angiotensin near/3 receptor* near/3 (inhibit* or antagonist* or block*))):ti,ab,kw |
| 13 | ((calcium near/2 (inhibit* or antagonist* or block*))):ti,ab,kw |
| 14 | ((adrenergic near/3 beta* near/3 (inhibit* or antagonist* or block*))):ti,ab,kw |
| 15 | ((adrenergic near/3 alpha* near/3 (inhibit* or antagonist* or block*))):ti,ab,kw |
| 16 | (((loop or ceiling) next diuretic*)):ti,ab,kw |
| 17 | ((amiloride or benzothiadiazine or bendroflumethiazide or bumetanide or chlorothiazide or cyclopenthiazide or furosemide or hydrochlorothiazide or hydroflumethiazide or methyclothiazide or metolazone or polythiazide or trichlormethiazide or veratide or thiazide*)):ti,ab,kw |
| 18 | ((chlorthalidone or chlortalidone or phthalamudine or chlorphthalidolone or oxodoline or thalitone or hygroton or indapamide or metindamide or "s-1520" or s1520 or "se-1520" or se1520)):ti,ab,kw |
| 19 | ((alacepril or altiopril or benazepril or captopril or ceronapril or cilazapril or delapril or enalapril or fosinopril or idapril or imidapril or lisinopril or moexipril or moveltipril or pentopril or perindopril or quinapril or ramipril or spirapril or temocapril or trandolapril or zofenopril or aliskiren or remikiren)):ti,ab,kw |
| 20 | (("KT3-671" or candesartan or eprosartan or irbesartan or losartan or olmesartan or tasosartan or telmisartan or valsartan)):ti,ab,kw |
| 21 | ((amlodipine or amrinone or bencyclane or bepridil or cinnarizine or conotoxins or diltiazem or felodipine or fendiline or flunarizine or gallopamil or isradipine or lidoflazine or "magnesium sulphate" or mibefradil or nicardipine or nifedipine or nimodipine or nisoldipine or nitrendipine or perhexiline or prenylamine or verapamil or "omega-agatoxin iva" or "omega-conotoxin gvia" or "omega-conotoxins")):ti,ab,kw |
| 22 | ((methyldopa or alphamethyldopa or amodopa or dopamet or dopegyt or dopegit or dopegite or emdopa or hyperpax or hyperpaxa or "methylpropionic acid" or dopergit or meldopa or methyldopate or medopa or medomet or sembrina or aldomet or aldometil or aldomin or hydopa or methyldihydroxyphenylalanine or "methyl dopa" or mulfasin or presinol or presolisin or sedometil or sembrina or taquinil or dihydroxyphenylalanine or methylphenylalanine or methylalanine or "alpha methyl dopa")):ti,ab,kw |
| 23 | ((reserpine or serpentina or rauwolfia or serpasil)):ti,ab,kw |
| 24 | ((clonidine or adesipress or arkamin or caprysin or catapres* or catasan or chlofazolin or chlophazolin or clinidine or clofelin* or clofenil or clomidine or clondine or clonistada or clonnirit or clophelin* or dichlorophenylaminoimidazoline or dixarit or duraclon or gemiton or haemiton or hemiton or imidazoline or isoglaucon or klofelin or klofenil or "m-5041t" or normopresan or paracefan or "st-155" or "st 155" or "tesno timelets")):ti,ab,kw |
| 25 | ((hydralazin* or hydrallazin* or hydralizine or hydrazinophtalazine or hydrazinophthalazine or hydrazinophtalizine or dralzine or hydralacin or hydrolazine or hypophthalin or hypoftalin or hydrazinophthalazine or idralazina or "1-hydrazinophthalazine" or apressin or nepresol or apressoline or apresoline or apresolin or alphapress or alazine or idralazina or lopress or plethorit or praeparat)):ti,ab,kw |
| 26 | ((acebutolol or adimolol or afurolol or alprenolol or amosulalol or arotinolol or atenolol or befunolol or betaxolol or bevantolol or bisoprolol or bopindolol or bornaprolol or brefonalol or bucindolol or bucumolol or bufetolol or bufuralol or bunitrolol or bunolol or bupranolol or butofilolol or butoxamine or carazolol or carteolol or carvedilol or celiprolol or cetamolol or chlortalidone cloranolol or cyanoiodopindolol or cyanopindolol or deacetylmetipranolol or diacetolol or dihydroalprenolol or dilevalol or epanolol or esmolol or exaprolol or falintolol or flestolol or flusoxolol or hydroxybenzylpinodolol or hydroxycarteolol or hydroxymetoprolol or indenolol or iodocyanopindolol or iodopindolol or iprocrolol or isoxaprolol or labetalol or landiolol or levobunolol or levomoprolol or medroxalol or mepindolol or methylthiopropranolol or metipranolol or metoprolol or moprolol or nadolol or oxprenolol or penbutolol or pindolol or nadolol or nebivolol or nifenalol or nipradilol or oxprenolol or pafenolol or pamatolol or penbutolol or pindolol or practolol or primidolol or prizidilol or procinolol or pronetalol or propranolol or proxodolol or ridazolol or salcardolol or soquinolol or sotalol or spirendolol or talinolol or tertatolol or tienoxolol or tilisolol or timolol or tolamolol or toliprolol or tribendilol or xibenolol)):ti,ab,kw |
| 27 | ((alfuzosin or bunazosin or doxazosin or metazosin or neldazosin or prazosin or silodosin or tamsulosin or terazosin or tiodazosin or trimazosin)):ti,ab,kw |
| 28 | {or #5-#27} |
| 29 | (continu* or discontinu* or temporar*):ti,ab,kw |
| 30 | #4 and #28 and #29 |
| 31 | [mh "infant, newborn"] or [mh "pediatrics"] or [mh "adolescent"] |
| 32 | (cardi* or myocard* or heart or coronary):ti,ab,kw |
| 33 | (child* or p?ediat* or neonat* or newborn* or infant* or baby* or babies or toddler* or minors* or adolesc* or preteen* or teen* or juvenil* or youth* or preschool* or school* or kindergarten* or kid*):ti,ab,kw |
| 34 | {or #31-#33} |
| 35 | #30 not #34 |
